# Distinct type 2-high inflammation associated molecular signatures of chronic rhinosinusitis with nasal polyps with comorbid asthma

**DOI:** 10.1186/s13601-020-00332-z

**Published:** 2020-07-03

**Authors:** Ming Wang, Xiangting Bu, Ge Luan, Liqing Lin, Yang Wang, Jianmin Jin, Luo Zhang, Chengshuo Wang

**Affiliations:** 1 Department of Otolaryngology, Head and Neck Surgery, Beijing TongRen Hospital, Capital Medical University, No. 1, DongJiaoMinXiang, DongCheng District, Beijing, 100730 China; 2 Beijing Key Laboratory of Nasal Diseases, Beijing Institute of Otolaryngology, Beijing, 100005 China; 3 Department of Allergy, Beijing TongRen Hospital, Capital Medical University, Beijing, 100730 China; 4 Department of Respiratory Medicine, Beijing Tongren Hospital, Capital Medical University, Beijing, 100730 China

**Keywords:** Chronic rhinosinusitis with nasal polyps, Asthma, Type 2 inflammation, Molecular endotype, Transcriptome sequencing

## Abstract

**Background:**

Patients with chronic rhinosinusitis with nasal polyps (CRSwNP) and comorbid asthma have more severe disease and are difficult to treat. However, the molecular endotypes associated with CRSwNP with comorbid asthma (CRSwNP + AS) are not clear. This study aimed to investigate the characteristics of type 2 inflammation and the molecular signatures associated with CRSwNP + AS.

**Methods:**

A total of 195 subjects; including 65 CRSwNP + AS patients, 99 CRSwNP-alone patients, and 31 healthy control subjects; were enrolled in the study. Nasal tissues from patients with CRSwNP + AS, CRSwNP-alone and control subjects were assessed for infiltration of inflammatory cells and concentrations of total IgE. Whole-transcriptome sequencing was performed and differentially expressed (DE) mRNAs and long non-coding RNAs (lncRNAs) and their associated pathways were analyzed. The correlations between type 2 cytokines and local eosinophils, tissue IgE, and transcriptome signatures were evaluated.

**Results:**

Significantly higher local eosinophil infiltration and higher levels of total IgE were found in nasal tissues from CRSwNP + AS patients than in nasal tissues from CRSwNP-alone patients. Furthermore, atopy and recurrence were significantly more frequent in patients with CRSwNP + AS than in patients with CRSwNP-alone (62.5% vs 28.6% and 66.7% vs 26.9%, respectively). RNA sequencing analysis identified 1988 common DE-mRNAs, and 176 common DE-lncRNAs shared by CRSwNP + AS versus control and CRSwNP-alone versus control. Weighted gene coexpression network analysis (WGCNA) identified LINC01146 as hub lncRNA dysregulated in both subtypes of CRSwNP. Overall, 968 DE-mRNAs and 312 DE-lncRNAs were identified between CRSwNP + AS and CRSwNP-alone. Both pathway enrichment analysis and WGCNA indicated that the phenotypic traits of CRSwNP + AS were mainly associated with higher activities of arachidonic acid metabolism, type 2 cytokines related pathway and fibrinolysis pathway, and lower activity of IL-17 signalling pathway. Furthermore, the expression of type 2 cytokines; IL5 and IL13, was positively correlated with local eosinophil infiltration, tissue IgE level, and the expression of DE-mRNAs that related to arachidonic acid metabolism. Moreover, WGCNA identified HK3-006 as hub lncRNA in yellow module that most positively correlated with phenotypic traits of CRSwNP + AS.

**Conclusions:**

Patients with CRSwNP + AS have distinct type 2-high inflammation-associated molecular signatures in nasal tissues compared to patients with CRSwNP-alone.

## Background

Chronic rhinosinusitis (CRS), a disease characterized by chronic inflammation of the sinonasal tissue, affects 5.5–28% of the general population [1]. CRS with nasal polyps (CRSwNP) accounts for approximately 20% of all CRS and has greater severity of clinical disease [2]. Asthma is one of the most common chronic inflammatory disorders of the lower airway worldwide with increasing morbidity. Studies have reported that up to 60% of CRSwNP patients have comorbid asthma (CRSwNP + AS), which is one of the most challenging CRS subtypes to treat [3]. Patients with CRSwNP + AS have greater disease severity, higher recurrence rates of nasal polyps after surgery, poorer asthma control and higher costs [4–6].

There is evidence that eosinophilic CRSwNP tends to have comorbid asthma more frequently [7]. Formation of IgE, which is independent of the presence of allergy in nasal polyp tissue, is also associated with asthmatic condition in patients with CRSwNP [8]. However, there is still no clear explanation for the association between CRSwNP and asthma, and likewise the pathogenic mechanisms leading to CRSwNP and asthma are uncertain. The united airway concept suggests that the upper and lower airway inflammation share common pathogenic mechanisms and influence each other [9, 10]. There is evidence that several features of inflammatory pattern, disrupted epithelial barrier and airway remodelling are similar in CRSwNP and asthma [11–13]. Thus, understanding the molecular relationship between CRSwNP and comorbid asthma may help to reveal the mechanisms that underlie airway chronic inflammation.

CRSwNP is a heterogeneous inflammatory condition with different endotypes [14]. The majority of white patients with CRSwNP in western countries have a type 2 pattern of inflammation characterized by pronounced eosinophilia and high levels of interleukin-4 (IL-4), IL-5 and IL-13 cytokines [15]. In contrast, Chinese patients with CRSwNP have lower type 2 inflammation and show higher degree of type 1/type 3 inflammation [16, 17]. Furthermore, evidence from previous studies on epidemiology and clinical characteristics, suggests that CRSwNP + AS may be considered a subtype of CRSwNP [18–20]. Thus, this study aimed to investigate the characteristics of type 2 inflammation and molecular endotypes associated with CRSwNP + AS by whole-transcriptome sequencing. Distinct type 2-high inflammation and its associated transcriptome signatures, indicated by coding mRNAs and long non-coding RNAs (lncRNAs), were found in patients with CRSwNP + AS compared to patients with CRSwNP-alone.

## Materials and methods

### Subjects

A total of 195 subjects, including 65 CRSwNP patients with comorbid asthma (CRSwNP + AS), 99 patients with CRSwNP-alone and 31 healthy control subjects were enrolled in series from June 2017 to March 2018 in the Rhinology Department of Beijing TongRen Hospital. Patients with CRSwNP were diagnosed according to the European Position Paper on Rhinosinusitis and Nasal Polyps 2020 guidelines [1]. The diagnosis of comorbid asthma was based on the Global Initiative for Asthma 2019 guidelines. The diagnosis of allergic rhinitis was according to Allergic Rhinitis and Its Impact on Asthma 2016 guidelines. Atopy was confirmed based on positive test for serum antigen-specific IgE (cut-off value, 0.35kUA/L), measured by Immuno-CAP 100 system (Pharmacia, Uppsala, Sweden). Patients undergoing septoplasty because of anatomic variations and without other sinonasal diseases were recruited as control subjects. All subjects were aged 18 to 70 years. Subjects with immunodeficiency, fungal sinusitis, coagulation disorder, neoplasia, pregnancy and aspirin-exacerbated respiratory disease (AERD) were excluded. None of the patients had been treated with corticosteroids, antibiotics or biologics within the 4-week period before surgery, and no patient had symptoms of infection at the time of sampling, apart from symptoms of chronic rhinosinusitis or asthma. Recurrence was defined as the presence of nasal polyps observed under nasal endoscopy, together with at least one symptom (nasal obstruction, rhinorrhea, headache/facial pain, reduction or loss of smell, sleep disturbance/fatigue) lasting at least 1 week, despite appropriate intranasal corticosteroid treatment. The postoperative follow-up period was 18 months for assessment of recurrence/non-recurrence of nasal polyps [1, 21]. All patients were followed-up after weeks 1, 2, 4, and 12, and then once every 3 months for up to 18 months by the same surgeon, who was blinded to all laboratory data [21]. Nasal tissue samples were collected from the inferior turbinate of control subjects and the nasal polyps of CRSwNP patients during surgery. The tissue samples were processed for staining with haematoxylin and eosin, RNA sequencing and ELISA as previously described [22].The Ethics Committee of Beijing Tongren Hospital approved this study, and all subjects signed informed consent forms prior to enrolment in the study.

### Histological evaluation of polyp tissue

Nasal polyp tissues were immediately formalin fixed after surgery, and then dehydrated and embedded in paraffin. Paraffin sections were stained with haematoxylin and eosin (H&E) and processed for histological evaluation. All sections were examined by optical microscopy at × 400 magnification. The absolute numbers and percentages of infiltrating inflammatory cells; including eosinophils, neutrophils, plasma cells, and lymphocytes; were recorded as mean of six non-overlapping regions in each section by two independent pathologists, who were blinded to the study design and clinical background of the patients.

### Assessment of total IgE in nasal tissues

Concentrations of total IgE in nasal tissues were assayed using the Human IgE ELISA Kit (Arigo Biolaboratories Corporation, Taiwan). Briefly, fresh nasal tissues were placed into RIPA lysis buffer with 1% protease inhibitor cocktail (Thermo Fisher Scientific) and homogenized using a standard bench-top homogenizer (Qiagen, Valencia, CA). The tissue homogenates were centrifuged and supernatants were collected for IgE analysis, according to the manufacturer’s instructions. All samples were tested in duplicate.

### RNA isolation and RNA sequencing

Nasal tissue samples of CRSwNP + AS (n = 10), CRSwNP-alone (n = 10), and control (n = 9) were randomly selected for whole-transcriptome sequencing. The nasal tissue samples collected after surgery were freshly preserved in RNAlater solution (Qiagen, Hilden, Germany) and processed for extraction and purification of total RNA using the RNeasy Kit (Qiagen, Hilden, Germany), according to the manufacturer’s instructions. The quantity and quality of the isolated RNA was determined with NanoDrop 2000 Spectrophotometer (Thermo Fischer Scientific) and 2100 TapeStation Automated Electrophoresis System (Agilent Technologies), and samples with an RNA integrity number of greater than 8.0 were chosen for sequencing. Ribosomal RNA was removed and sequencing libraries were prepared using the rRNA-depleted RNA by NEBNext UltraTM Directional RNA Library Prep Kit (New England Biolabs, USA), following the manufacturer’s instructions. RNA sequencing was performed on the Illumina Hiseq platform and 150 bp paired-end reads were generated by Novogene Bioinformatics Technology Cooperation (Beijing, China).

### RNA sequencing data analysis

Adapters and low-quality tail were trimmed from reads prior to read alignment. Clean sequence reads were aligned to the human genome with Hisat2 (v2.0.5). Cufflinks (v2.2.1) was used to assemble transcripts, estimate the abundance of these transcripts, and detect differential expression among samples. For mRNA analyses, the reference genome build GRCh37 was chosen as the annotation references. For lncRNA analyses, the GENCODE v19 database was chosen as the annotation references. Fragments per kilo-base of exon per million fragments mapped (FPKM) of both lncRNAs and mRNAs in each sample was calculated based on the length of the fragments and reads count mapped to this fragment. Differential expression analysis was performed using Cuffdiff software (v2.2.1). An adjusted *P *< 0.05 plus fold change > 2 was used as the cut-off for significantly differentially expressed (DE) mRNAs (DE-mRNAs) and lncRNAs (DE-lncRNAs).

### Pathway analysis of DE-mRNAs and function prediction of DE-lncRNAs

DE-mRNAs were loaded into Enrichr (https://amp.pharm.mssm.edu/Enrichr/) [23] for pathway enrichment analysis, and the top significantly enriched (*P *< 0.05) KEGG and Biocarta pathways with high Combined Score, provided by Enrichr, were determined.

In order to predict DE-lncRNA functions, we applied weighted gene coexpression network analysis (WGCNA) [24] to construct a coexpression network between DE-lncRNAs and their highly correlated DE-mRNAs based on the Pearson correlation coefficient between their normalized expression levels. Briefly, network construction and module detection were performed using the “blockwise Modules” function in the WGCNA package. A coexpression similarity matrix was calculated by computing Pearson correlations between all gene pairs, and then transformed into an adjacency matrix using a soft threshold power (β) equal to 18. A dynamic tree cut algorithm was used to detect groups of highly correlated genes. Modules were defined as the branches cut-off of the tree and each module was labelled in unique colours, of which grey colour contains probes not assigned to any module. The module eigengenes were utilized to represent each module, which was calculated via the first principal component. Using the module eigengenes, the relationship between module and tissue type (CRSwNP-alone and CRSwNP + AS) was estimated.

Enrichr was used to perform the functional enrichment analysis for each module, and the top significantly enriched pathways were determined. In interesting modules that related to the disease condition, top hub genes with high connectivity and edges with weight above a threshold of 0.1 were identified and visualized using the cytoscape network.

### Statistical analysis

All data are presented as medians and interquartile range (IQR) except for age, which is presented as mean ± SD. Data analysis was performed using GraphPad Prism Version 7.0 (GraphPad Software, La Jolla, Calif). All parametric variants were analyzed using Student t tests, and nonparametric variants were analyzed by using Mann–Whitney *U* tests. The χ^2^ or Fisher exact test was used for qualitative data. Relationships between variables were evaluated using Spearman correlation analysis. Differences were considered significant at *P* value < 0.05.

## Results

### Demographic and clinical characteristics of the subjects

**Fig. 1:**
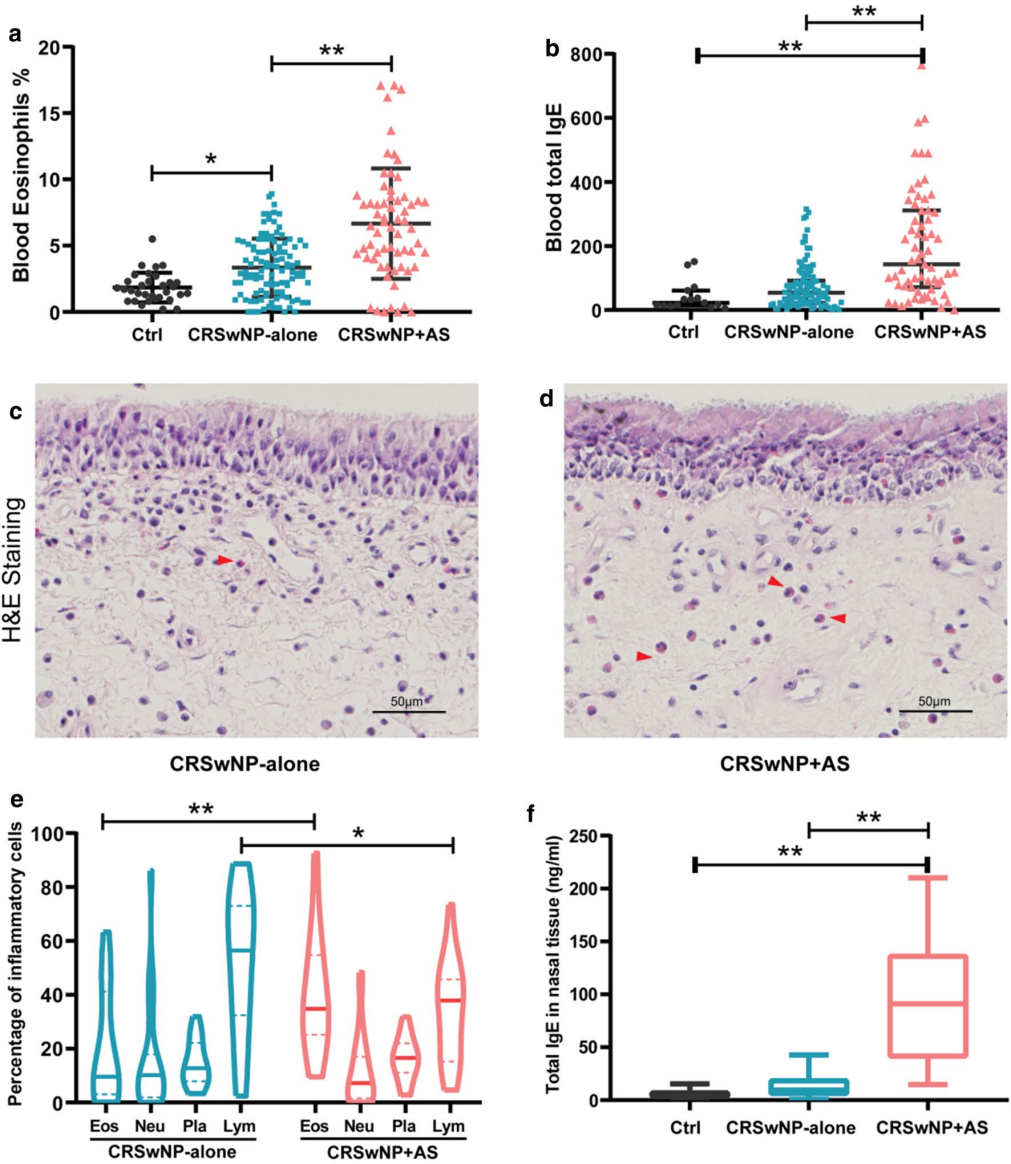
Detection of eosinophils and total IgE in blood and nasal tissues from patients with CRSwNP. **a**, **b** Blood eosinophils and total IgE was detected in CRSwNP patients with asthma (CRSwNP + AS), CRSwNP-alone and control (Ctrl) subjects. **c**, **d** Representative images of haematoxylin and eosin-stained nasal polyp tissues from patients with CRSwNP-alone and CRSwNP + AS. Red arrows point out eosinophils. **e** The percentage of infiltrating eosinophils, neutrophils, plasma cells and lymphocytes were assessed in nasal tissues of CRSwNP-alone (n = 24) and CRSwNP + AS (n = 25). **f** Concentrations of total IgE in nasal tissues of Ctrl (n = 10), CRSwNP-alone (n = 14) and CRSwNP + AS (n = 17) were assayed using Human IgE ELISA Kit. Data are presented as medians and interquartile range (IQR). Data shown in **a** adjusted for smoking, and in **b** and **f** adjusted for atopy. *P < 0.05, **P < 0.01, Mann–Whitney *U* tests. *CRSwNP* chronic rhinosinusitis with nasal polyps, *AS* asthma

Demographic and clinic characteristics of all participants enrolled in this study are presented in the Additional file 1: Table S1. There was no significant difference with regard to age, gender and smoker status between the 3 groups. Peripheral blood eosinophils and total IgE were increased in both subtypes of CRSwNP patients compared to control subjects. Assessment for the significance of differences between CRSwNP + AS group and CRSwNP-alone group indicated that atopy and recurrence were significantly more frequent in patients with CRSwNP + AS than in patients with CRSwNP-alone (62.5% vs 28.6% and 66.7% vs 26.9%, respectively). Patients with CRSwNP + AS also had a significantly decreased forced exhalation volume in one second (FEV1)/forced vital capacity (FVC) ratio and an increased fractional exhaled nitric oxide (FeNO) compared to patients with CRSwNP-alone. Similarly, CRSwNP + AS patients had a significantly higher percentage of eosinophils (6.45%) and total IgE (143.00 kU/l) in peripheral blood compared to CRSwNP-alone patients (3.00% and 53.90 kU/l, respectively; Fig. 1a, b).

### Local features of nasal tissue in different patient group

To identify the local inflammatory patterns of nasal tissue in CRSwNP patients with and without comorbid asthma, the infiltrating eosinophils, neutrophils, plasma cells, and lymphocytes were assessed. Tissue sections stained with H&E demonstrated that patients with CRSwNP + AS had significantly more eosinophils in nasal polyp tissues than patients with CRSwNP-alone (Fig. 1c–e).

Given the relationship between IgE and Type 2 immune response, we further examined the total IgE levels in nasal tissues and found that IgE levels in nasal tissues from CRSwNP + AS patients were significantly higher compared to IgE levels in nasal tissues of CRSwNP-alone patients and controls (Fig. 1f). Moreover, Spearman correlation test showed that the percentage of local eosinophils was positively correlated with the concentration of tissue IgE (Additional file 1: Figure S1).

### Whole transcriptome profiling of nasal tissues from CRSwNP + AS and CRSwNP-alone

To identify the gene expression profiles of CRSwNP with and without comorbid asthma, RNA sequencing was performed on nasal tissue samples from control, CRSwNP-alone and CRSwNP + AS patients. Analysis of DE-mRNAs and DE-lncRNAs demonstrated that there were 5218 DE-mRNAs and 2949 DE-lncRNAs between CRSwNP-alone and control, and 2512 DE-mRNAs and 464 DE-lncRNAs between CRSwNP + AS and control (Additional file 1: Figure S2). Additionally, 968 DE-mRNAs and 312 DE-lncRNAs were identified between CRSwNP + AS and CRSwNP-alone. Hierarchical clustering of top 500 differentially expressed genes was shown in Additional file 1: Figure S3.

### Common dysregulated genes shared by CRSwNP + AS and CRSwNP-alone

**Fig. 2:**
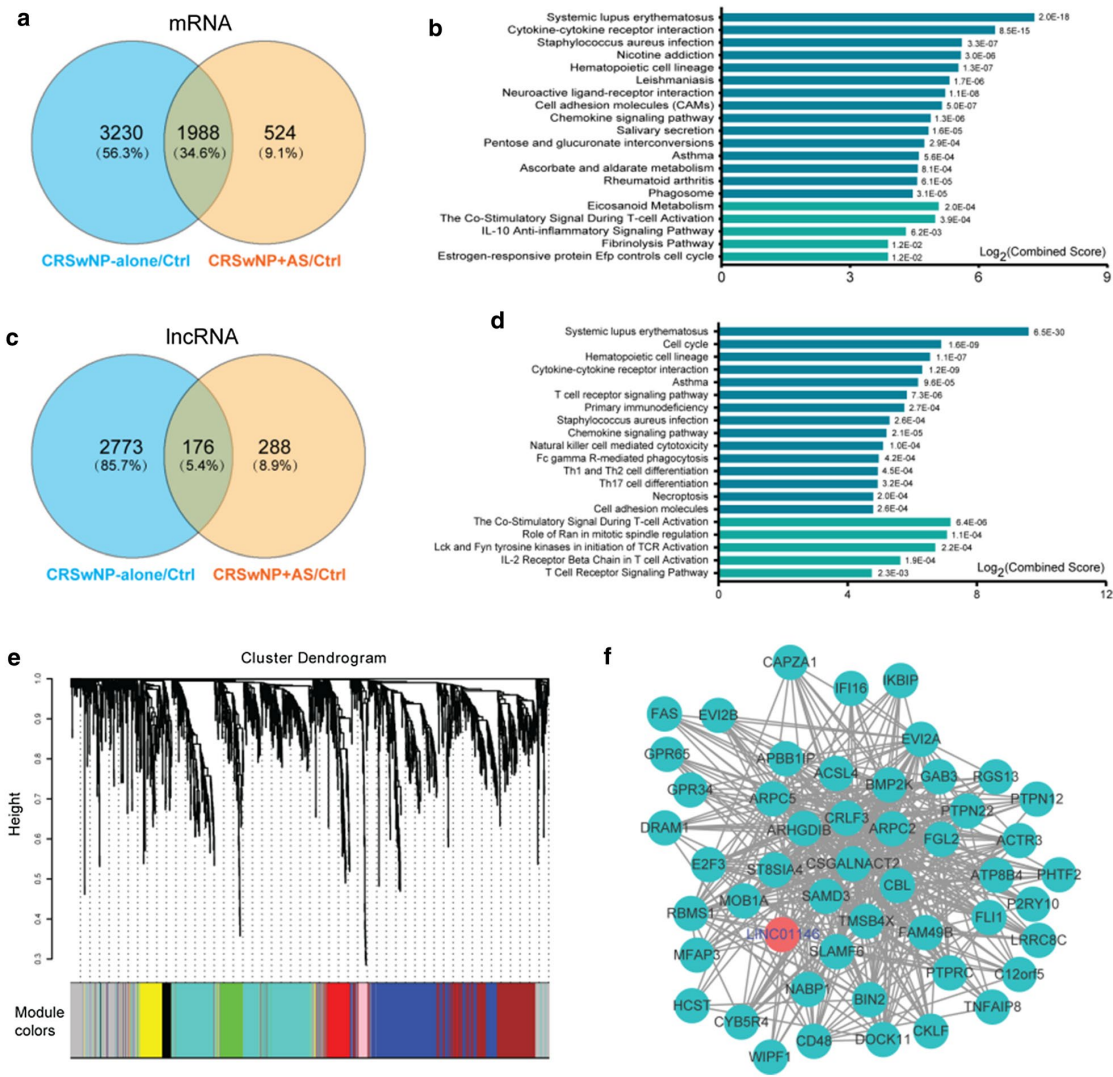
Common dysregulated genes shared by CRSwNP + AS and CRSwNP-alone. Nasal tissue samples of CRSwNP + AS (n = 10), CRSwNP-alone (n = 10), and control (n = 9) were analysed by whole-transcriptome sequencing. **a** Venn diagrams depicting DE-mRNAs of CRSwNP + AS versus control and CRSwNP-alone versus control. The number of DE-mRNAs is marked in the corresponding areas. **b** The 1988 common DE-mRNAs shared by CRSwNP + AS versus control and CRSwNP-alone versus control were assessed by pathway enrichment analyses using Enrichr. Top 15 significantly enriched KEGG pathways (blue columns) and top 5 significantly enriched BioCarta pathways (turquoise columns) are depicted. *P *< 0.05 were considered statistically significant. **c** Venn diagrams depicting DE-lncRNAs of CRSwNP + AS versus control and CRSwNP-alone versus control. **d, e** The 176 common DE-lncRNAs shared by CRSwNP + AS versus control and CRSwNP-alone versus control were assessed for expression based modules identified by weighted gene coexpression network analysis (WGCNA) and for their potential functions, based on a coexpression network. **e** Branches of the dendrogram obtained by hierarchical clustering of adjacency based similarity show 9 modules, labelled with a distinct colour, and **d** Top 15 significantly enriched KEGG pathways (blue column) and top 5 significantly enriched BioCarta pathways (turquoise column) by genes in the largest turquoise module. **f** Top 50 hub genes of turquoise module visualized by cytoscape network. mRNAs or lncRNAs with high connectivity and edges with weight above a threshold of 0.1 were identified as hub genes. The red nodes denote lncRNAs, and the green nodes denote mRNAs. *CRSwNP* chronic rhinosinusitis with nasal polyps, *AS* asthma, *lncRNA* long non-coding RNA, *DE* differentially expressed, *KEGG* Kyoto Encyclopedia of Genes and Genomes

A total of 1988 common DE-mRNAs were shared by CRSwNP-alone and CRSwNP + AS respectively compared to control (Fig. 2a and Additional file 1: Table S2). Assessment of these common DE-mRNAs by pathway enrichment analysis using Enrichr Demonstrated that the top enriched KEGG pathways were associated with cytokine–cytokine receptor interaction, chemokine signalling pathway, staphylococcus aureus infection, asthma, and cell adhesion molecules (Fig. 2b). The top enriched BioCarta pathways were related to eicosanoid metabolism, the co-stimulatory signal during T cell activation and IL-10 anti-inflammatory signalling pathway (Fig. 2b). Likewise, gene ontology enrichment analysis showed that common DE-mRNAs were mainly associated with regulation of immune system process, immune response, response to stimulus and cell–cell signalling (Additional file 1: Figure S4).

Similarly, a total of 176 common DE-lncRNAs were shared by CRSwNP + AS versus control and CRSwNP-alone versus control (Fig. 2c and Additional file 1: Table S2). WGCNA was applied to explore the potential functions of common DE-lncRNAs, and construction of a hierarchical clustering tree demonstrated 9 modules, each of which was labelled with a distinct colour (Fig. 2e). The size of these modules ranged from 41 to 539 genes; with the largest turquoise coloured module (Additional file 1: Figure S5) comprising 45 lncRNAs and 494 mRNAs, which were highly coexpressed. Pathway enrichment analysis showed that the genes of this turquoise module were mainly associated with cytokine–cytokine receptor interaction, asthma, T cell receptor signalling pathway, staphylococcus aureus infection, chemokine signalling pathway, and Th1, Th2, Th17 cell differentiation (Fig. 2d).

Figure 2f shows the top 50 hub genes with high connectivity and edge weigh identified and visualized by cytoscape network. LINC01146, the only one lncRNA of these top hub genes, was significantly up-regulated in both CRSwNP-alone and CRSwNP + AS. Pathway analysis of the coexpressed mRNAs of LINC01146 demonstrated that LINC01146 was mostly associated with T cell receptor signalling pathway, natural killer cell mediated cytotoxicity, Fc gamma R-mediated phagocytosis, and Th1 and Th2 cell differentiation (Additional file 1: Figure S6).

### Distinct transcriptome signatures in nasal tissue of CRSwNP + AS

**Fig. 3:**
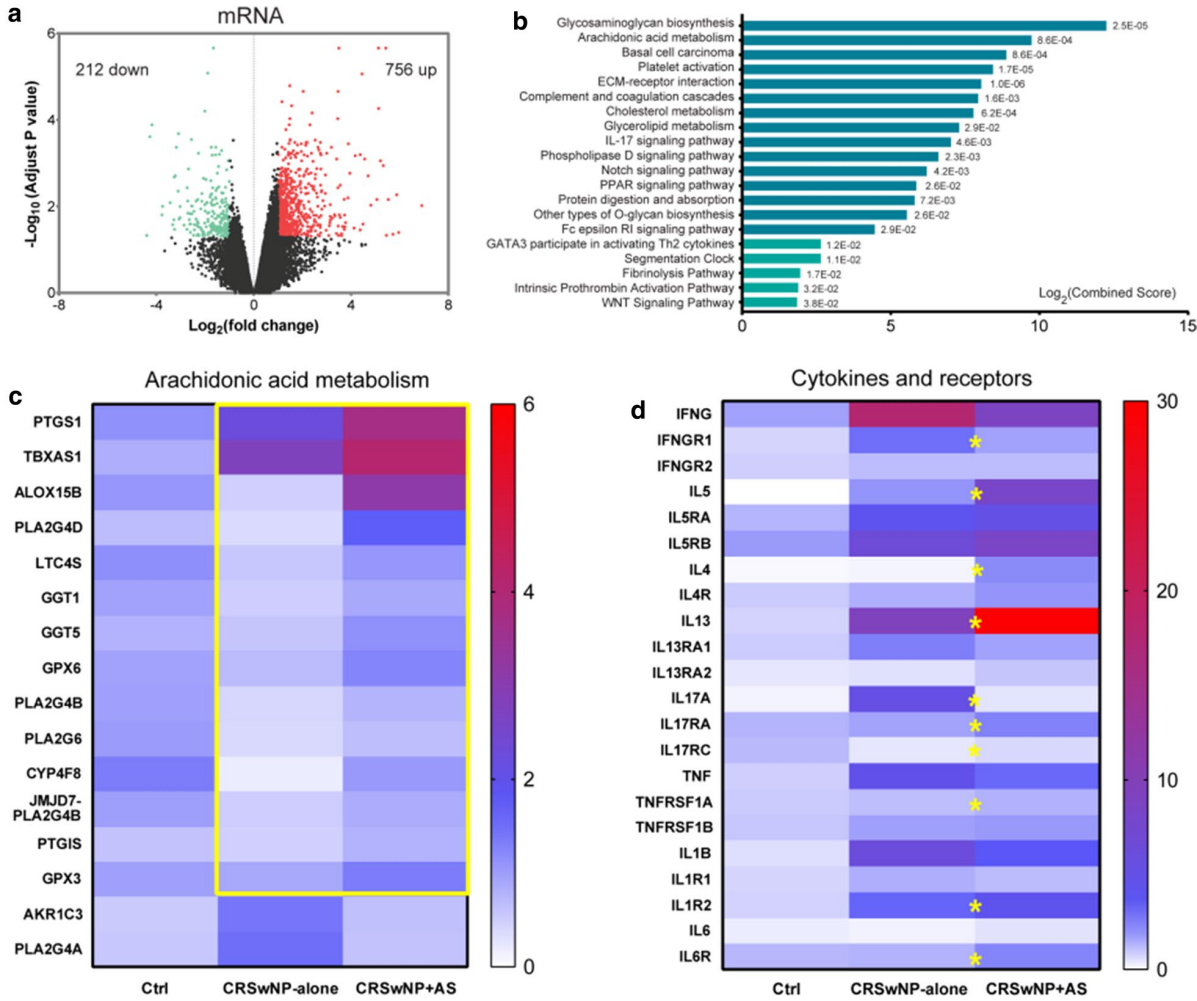
Differentially expressed genes and pathways between CRSwNP + AS and CRSwNP-alone. **a** Volcano plots illustrating DE-mRNAs of CRSwNP + AS versus CRSwNP-alone identified by RNA sequencing. **b** Top 15 KEGG pathways (blue column) and top 5 BioCarta pathways (turquoise column) significantly enriched by DE-mRNAs. **c** The expression of arachidonic acid metabolism-related DE-mRNAs between CRSwNP + AS and CRSwNP-alone. The colour coding of heat maps represents the gene expression level normalized to Control group, calculated based on fragments per kilo-base of exon per million fragments mapped (FPKM). Yellow box indicates the up-regulated genes in CRSwNP + AS group. **d** The expression of critical cytokines and their receptors that indicated the activity of different inflammatory endotypes. Yellow stars represent significantly differentially expressed genes between CRSwNP + AS and CRSwNP-alone. *P *< 0.05 were considered statistically significant. *CRSwNP* chronic rhinosinusitis with nasal polyps, *AS* asthma, *DE* differentially expressed, *KEGG* Kyoto Encyclopedia of Genes and Genomes

Overall, 212 mRNAs were down-regulated and 756 mRNAs up-regulated in nasal tissues of CRSwNP + AS patients, compared to nasal tissues of CRSwNP-alone patients (Fig. 3a). The 50 most significant DE-mRNAs are shown in Additional file 1: Table S3. Enrichr pathway analysis showed that arachidonic acid metabolism, ECM-receptor interaction, IL-17 signalling pathway, GATA3 participate in activating Th2 cytokine genes, and fibrinolysis pathway were the top significant pathways enriched by DE-mRNAs (Fig. 3b).

Detailed examination of the expression of genes involved in arachidonic acid metabolism demonstrated that 14 of 16 DE-mRNAs related to arachidonic acid metabolism; including PTGS1, TBXAS1, ALOX15B, PLA2G4D, LTC4S, GGT1, GGT5, GPX6, PLA2G4B, PLA2G6, CYP4F8, JMJD7-PLA2G4B, PTGIS and GPX3; were up-regulated in CRSwNP + AS compared to CRSwNP-alone (Fig. 3c).

### Severe type 2 inflammation in nasal tissues of CRSwNP + AS

As indicated by above transcriptome data, type 1, type 2 and type 3 related signalling pathways might be differentiated between CRSwNP-alone and CRSwNP + AS. Thus, we investigated the mRNA expression of critical cytokines and their receptors, which indicate the activities of different inflammatory endotypes; namely (1) IFNG and IFNG receptor (IFNGR1 and IFNGR2) for type 1 inflammation; (2) IL5, IL4, IL13 and their receptors (IL5RA, IL5RB, IL4R, IL13RA1 and IL13RA2) for type 2 inflammation; (3) IL17A and IL17A receptor (IL17RA and IL17RC) for type 3 inflammation; as well as (4) TNF, IL1B, IL6 and their receptors (TNFRSF1A, TNFRSF1B, IL1R1, IL1R2 and IL6R) for proinflammatory activity.

We found that the expression of cytokines IL5 and IL13, and receptors IL5RA and IL5RB, indicating augmented type 2 inflammation, was significantly enhanced in both CRSwNP-alone and CRSwNP + AS compared to control (Fig. 3d and Additional file 1: Table S4). Furthermore, nasal tissue from CRSwNP + AS demonstrated significantly higher expression of IL4, IL5 and IL13; and conversely significantly lower expression of IL17A than nasal tissue of CRSwNP-alone.

**Fig. 4:**
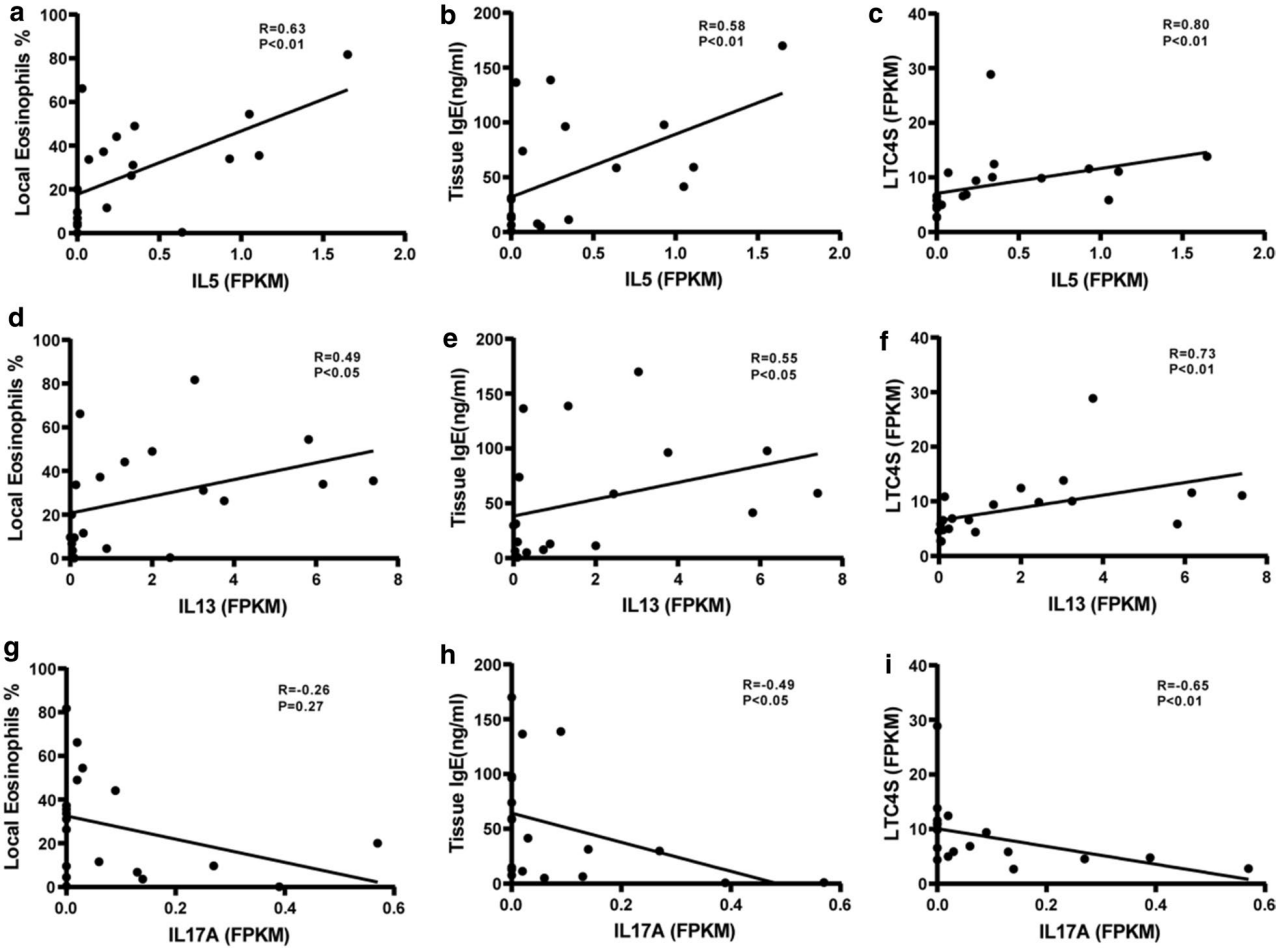
Correlations between cytokines and inflammatory indicators. Spearman correlation analysis was performed between expression of cytokines IL-5, IL-13, and IL-17A and percentage of local eosinophils, concentration of tissue IgE and LTC4S expression. N = 20 for each group. *FPKM* fragments per kilo-base of exon per million fragments mapped, *IL* interleukin-4

Assessment of correlations between the significant cytokines and the other significant inflammatory indicators determined above, demonstrated that the expression of both IL5 and IL13 was positively correlated with the percentage of local eosinophils, concentration of tissue IgE and the expression of LTC4S, which reflected an imbalanced arachidonic acid metabolism (Fig. 4a–f). In contrast, the expression of IL17A was negatively correlated with tissue IgE and the expression of LTC4S (Fig. 4g–i).

### LncRNA signatures in nasal tissue of CRSwNP + AS

**Fig. 5:**
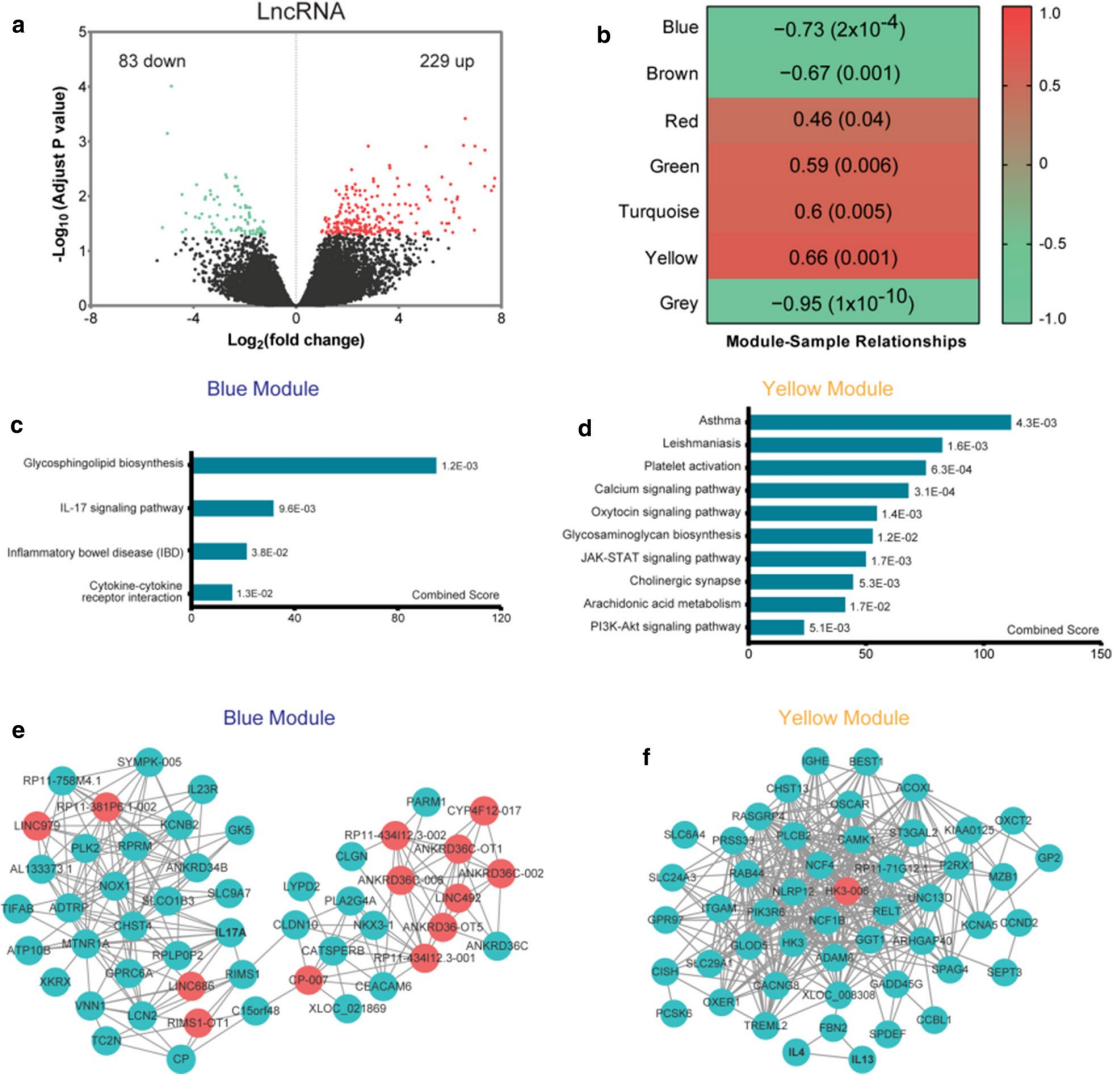
Differentially expressed lncRNAs and pathway analysis between CRSwNP + AS and CRSwNP-alone. **a** Volcano plots illustrating DE-lncRNAs of CRSwNP + AS versus CRSwNP-alone identified by RNA sequencing. **b** The correlation between modules and phenotype of CRSwNP + AS. Seven modules were identified by WGCNA based on expression of DE-mRNAs and DE-lncRNAs of CRSwNP + AS versus CRSwNP-alone. Pearson’s correlation coefficient between each module and phenotype of CRSwNP + AS and their associated *P* values are shown in the corresponding modules. The red and green colours show a strong positive and negative correlation, respectively. **c, d** All or top 10 KEGG pathways significantly enriched by genes in blue module **c** and yellow module (**d**). **e, f** Top 50 hub genes in blue module (**e**) and yellow module (**f**) visualized by cytoscape network. mRNAs or lncRNAs with high connectivity and edges with weight above a threshold of 0.1 were identified as hub genes. The red nodes denote lncRNAs, and the green nodes denote mRNAs. *P *< 0.05 were considered statistically significant. *CRSwNP* chronic rhinosinusitis with nasal polyps; AS: asthma, *lncRNA* long non-coding RNA, *DE* differentially expressed, *KEGG* Kyoto Encyclopedia of Genes and Genomes

We identified 229 up-regulated and 83 down-regulated DE-lncRNAs in nasal tissues of CRSwNP + AS patients compared to nasal tissues of CRSwNP-alone patients (Fig. 5a). The top 50 significant DE-lncRNAs are shown in Additional file 1: Table S5. A coexpression network constructed based on the expression of DE-lncRNAs and DE-mRNAs using WGCNA demonstrated 7 colour-coded modules underneath the cluster tree (Additional file 1: Figure S7). Assessment of the relationship between each module and tissue type (CRSwNP + AS), estimated using the module eigengenes, demonstrated that all 7 modules were significantly correlated with tissue type changes (Fig. 5b). Of these, the blue module was most negatively correlated with the phenotypic traits of CRSwNP + AS (r = − 0.73, *P *= 2 × 10^−4^), and the yellow module the most positively correlated (r = 0.66, *P *= 0.001).

Overall, there were 31 lncRNAs and 94 mRNAs in the blue module, and only one lncRNA (HK3-006) and 63 mRNAs in the yellow module. Pathway enrichment analysis indicated that the blue module was mainly associated with IL-17 signalling pathway and cytokine–cytokine receptor interaction (Fig. 5c), whereas the yellow module was mainly related to asthma, arachidonic acid metabolism, and signalling pathways such as calcium and JAK-STAT signalling pathway (Fig. 5d). Top 50 hub genes identified from the blue and yellow modules, respectively, are shown in Fig. 5 e, f.

## Discussion

Both CRSwNP and asthma are airway inflammatory disorders, which have serious effects on quality of life. A great proportion of CRSwNP patients have comorbid asthma, which makes it one of the most challenging phenotypes to treat [3, 12]. Furthermore, the molecular endotypes of CRSwNP + AS are not clear. In this regard, to our knowledge this is the first study to investigate the molecular endotypes of CRSwNP + AS compared to CRSwNP-alone, by whole-transcriptome RNA sequencing. Our study demonstrated that type 2-high inflammation patterns and their associated transcriptome features were distinct in nasal tissues of CRSwNP + AS patients compared to CRSwNP-alone patients.

Studies have indicated that CRS is a heterogeneous disease with several unclear endotypes, which are mainly characterized by type 1, 2, and 3 inflammatory patterns [15, 25–27]. Furthermore, some recent studies have indicated that the most prevalent endotype in CRSwNP is characterized by type 2 inflammation [28], and that type 2 inflammation in CRSwNP may be differentiated into moderate and severe type 2 inflammatory patterns according to the intensity of inflammation [29]. In accordance with these studies, the findings for the differentially expressed type 1, type 2 and type 3 inflammation-related genes and pathways in the present study suggest that the CRSwNP + AS endotype is associated with a more severe type 2 inflammation, compared to CRSwNP-alone endotype. Furthermore, in accordance with the findings of Tomassen and colleagues [15] that severe type 2 CRSwNP increased asthma prevalence, our study has also demonstrated that in addition to more frequent atopy, more local eosinophil infiltration, and higher level of tissue IgE, the chance of recurrence of CRSwNP was also increased in patients with CRSwNP + AS compared to patients with CRSwNP-alone. Indeed, we have previously shown that high proportion of eosinophils in nasal tissue act as a reliable prognostic indicator for CRSwNP recurrence [30]. Similarly, a more recent study has indicated that asthma in CRS patients was the only factor that increases the chance of recurrence in CRSwNP patients [6]; suggesting that comorbid asthma might also be a strong indicator for CRSwNP recurrence.

Airway type 2 inflammation is mainly mediated by eosinophils, mast cells, Th2 cells, ILC2s and IgE-producing B cells, in combination with increased production of cytokines IL-5, IL-4 and IL-13 [31]. Whilst more than 80% of Western white patients with CRSwNP are characterized by type 2 inflammation, less than 50% of CRSwNP cases in East Asian countries show features of type 2 reactions [32, 33]. Like CRSwNP, asthma is also a complex and heterogeneous disease, and two major endotypes of asthma, type 2-high asthma and type 2-low asthma, have been described, based on underlying airway immune-mediated inflammation [34, 35]. In this regard approximately 50% to 60% of all patients with severe asthma in Europe and the United States account for type 2-high asthma, compared to 38.5% of the severe asthmatic patients with type 2-high asthma in China [36–39]. Thus, whilst it is possible that generally more Western patients with CRSwNP + AS are also likely to be characterised with type 2-high inflammation than Asian patients CRSwNP + AS, this nevertheless needs to be confirmed in well-designed multicenter studies in the future.

Our findings for RNA sequencing provides valuable information for exploring the general molecular mechanisms underlying the pathogenesis of CRSwNP. Similar to the findings of Peng and colleagues [40], our study has demonstrated that regardless of the subtypes of CRSwNP, genes and pathways that most likely contribute to the pathogenesis of CRSwNP, appear to be mainly associated with cytokine and chemokine signalling pathway, staphylococcus aureus infection, eicosanoid metabolism and cell adhesion molecules. In this respect, the well-known genes or biomarkers closely related to CRSwNP; for example CLC, POSTN, CCL18, IL13, TSLP and BPIFA1; were also identified as the top DE-mRNAs in both CRSwNP-alone and CRSwNP + AS groups.

However, the transcriptome signatures of CRSwNP + AS were characterized by distinct groups of differentially expressed genes and their enriched pathways, compared to CRSwNP-alone. In particular, the present study showed that the CRSwNP + AS endotype was mainly associated with higher activities of arachidonic acid metabolism, Th2 signalling pathway and fibrinolysis pathway, and lower activity of IL-17 signalling pathway. Beyond that, the most significant DE-mRNAs between CRSwNP + AS and CRSwNP-alone also provide important information. In line with the high concentration of tissue IgE in CRSwNP + AS, increased mRNA expression of constant region of heavy chain of IgE (IGHE) was also confirmed by RNA sequencing. Some recently identified biomarkers of CRSwNP, for example CST1 [41], were also found to be differentially expressed between CRSwNP + AS and CRSwNP-alone. Importantly, the present study showed that some of the most significant DE-mRNAs; including ITLN1, KCNA3 and CCR10; which were up-regulated in nasal tissue of CRSwNP + AS compared to nasal tissue of CRSwNP-alone (Additional file 1: Figure S8), are also expressed in the bronchial tissue and contribute to the pathogenesis of asthma [42–44]. This suggests that signature genes identified in nasal tissue of CRSwNP + AS might also play important roles in the pathogenesis of asthma. Moreover, consistent with the united airways concept, it is tempting to speculate that the pattern of gene expression in the upper airway may be influenced by the prevailing conditions in the lower airway, and as such prevalence of comorbid asthmatic conditions might exacerbate type 2 inflammation in nasal tissue of CRSwNP patients.

It has been well demonstrated that alterations in the arachidonic acid pathway play an important role in airway inflammatory conditions like rhinosinusitis, nasal polyps, allergic rhinitis, and asthma [45]. We found a generally enhanced activity of arachidonic acid metabolism in CRSwNP + AS, indicated by the up-regulated expression of PLA2 (PLA2G4A, PLA2G4B, PLA2G4D and PLA2G6), which may promotes the release of membrane-bound arachidonic acid [46]. The imbalanced synthesis of eicosanoids characterized by increased synthesis of cysteinyl leukotrienes (CysLTs) is correlated with the inflammatory pattern and severity of the airway inflammation [45, 47]. Consistent with this, we also showed an increased expression of LTC4S, which promotes the biosynthesis of CysLTs and indicates the presence of severe inflammation in the nasal tissues of CRSwNP + AS patients. The increased expression of PTGS1 and PTGIS may accelerate the conversion of arachidonic acid to prostacyclins (PGI2) and prostaglandins (PGD2, PGE2), which are also related to an aggravated airway inflammation and asthmatic conditions [48, 49]. Moreover, the increased expression of ALOX15B in CRSwNP + AS might have an active proinflammatory role [50]. Collectively, these findings suggest that enhanced activity of arachidonic acid metabolism might help to augment type 2 inflammation in CRSwNP + AS [45, 51].

Th2 and Th17 signalling pathways are two major regulatory pathways, which are inversely interrelated in patients with CRSwNP [17]. In accordanc ewith this, general observation, our RNA sequencing data also revealed higher activities of Th2 signalling pathway and lower activities of IL-17 signalling pathway in CRSwNP + AS than in CRSwNP-alone. In this respect IL-4, IL-5 and IL-13 are typical Th2 cytokines, which reflect the severity of type 2 inflammation, whereas IL-17 is a typical Th17 cytokine. Indeed, the present study further showed that the expression of IL5 and IL13 was positively correlated with other indicators of type 2 inflammation (i.e. increased local eosinophils and tissue IgE). Similarly, genes that are related to arachidonic acid metabolism were also correlated with the expression of type 2 cytokines, thus, emphasising the crucial role of type 2 inflammation in CRSwNP + AS patients.

LncRNAs play important roles in various biological processes and are emerging as reliable biomarkers and potential therapeutic targets of human chronic diseases [52, 53]. However, comparatively few studies have reported the involvement of lncRNAs in chronic nasal inflammation. Yue and colleagues [54] found that linc00632 was down-regulated in nasal tissues of allergic rhinitis patients and inhibited IL-13 induced inflammatory cytokine and mucus production. Wang and colleagues [55] showed that lncRNA XLOC_010280 might regulate the expression of CCL18 and eosinophilic inflammation in eosinophilic CRSwNP. In the present study, whole-transcriptome sequencing has revealed several dysregulated lncRNAs in both subtypes of CRSwNP, which will provide a useful pool of candidate lncRNAs in future investigations of CRSwNP endotypes.

WGCNA is an effective method of multigene analysis to construct coexpression network, and has successfully been applied for studying mRNAs and lncRNAs to distinguish dysfunctional regulatory subnetworks, select out potentially key genes, and predict lncRNA functions [56, 57]. We applied WGCNA to predict functions of DE-lncRNAs and identify hub genes in the present study and showed that common dysregulated lncRNAs in CRSwNP-alone and CRSwNP + AS have very similar predictive functions to common DE-mRNAs, as indicated by genes in the maximal module. Furthermore, we identified LINC01146 as the only top hub lncRNA, which might play a key role in the pathogenesis of CRSwNP. Although LINC01146 has also been found to be dysregulated in hepatocellular carcinoma, its precise function in this condition is not clear [58]. Further study is needed to validate the function of common dysregulated lncRNAs in CRSwNP.

The present study also identified HK3-006 as the only top hub lncRNA that was expressed in the module that most positively correlated with phenotypic traits of CRSwNP + AS; and may be related to asthma pathway and arachidonic acid metabolism as predicted by its highly coexpressed mRNAs. Similarly, several top hub lncRNAs were identified in the module that most negatively correlated with phenotypic traits of CRSwNP + AS. Among these lncRNAs, we have identified a new lncRNA, LINC686, which is most likely to be associated with IL-17 signalling pathway. Although an increasing number of studies show that lncRNAs are involved in the regulation of cytokine signalling and inflammation [59, 60], our understanding of the functions of lncRNA is just beginning to develop.

The present study has some limitations. First, this study did not investigate any subgroups of CRSwNP + AS and CRSwNP-alone. This may be of importance as both CRSwNP and asthma are heterogeneous diseases, and one recent study has shown that patients with CRSwNP + AS can be grouped into 3 subtypes with distinct inflammatory status and disease severity [19]. However, the present study was designed to directly compare CRSwNP + AS with CRSwNP-alone. Furthermore, as a substantial number of Asian CRSwNP patients are type 1 and/or type 3 inclined, it would be more appropriate to compare type 2 CRSwNP endotype with type 2 CRSwNP + AS endotype for a better understanding of the mechanisms and pathways operating in either endotype. Second, as the sample size used for RNA sequencing is relatively small, this might decrease the statistical power for gene signature profiling. Third, we did not evaluate lower airway inflammation by assessment of inflammatory cells and cytokines in bronchial biopsy or induced sputum, which might be helpful in understanding the association between CRSwNP and asthma. Fourth, assessment of critical cytokines and their receptors was based only on RNA expression and need to be verified according to the levels of corresponding proteins in the tissue. Fifth, whole tissue samples with multiple cell types were used for RNA sequencing and no single-cell RNA sequencing was used. As single-cell RNA sequencing allows the analysis of transcriptome from individual cells in nasal tissues [61], this might have provided more specific information on the role of different cell types as biomarkers of CRSwNP with or without comorbid asthma. The use of inferior turbinate tissue from controls for comparison with nasal polyp tissues from CRSwNP patients is also as a limitation of the study, particularly as nasal polyps rarely arise from inferior turbinates in healthy control subjects. However, using mucosa tissue from different locations such as middle turbinate and ethmoid tissue as controls may avoid regional variations in gene expression.

## Conclusions

The present study demonstrated that CRSwNP patients with comorbid asthma have distinct type 2-high inflammation-associated transcriptome profiles, as indicated by differential expression profiles of key mRNAs and lncRNAs in nasal tissue, compared to patients with CRSwNP-alone. Differences in molecular mechanisms and type 2 inflammation-related molecules in CRSwNP with comorbid asthma may be useful in better understanding the mechanisms underlying the development of different endotypes of CRS, as well as development of potential biomarkers and targeted gene therapies for CRSwNP in the future.

## Supplementary information

**Additional file 1: Table S1.** Demographic and clinical characteristics of study subjects. **Table S2.** Common DE-mRNAs and DE-lncRNAs shared by CRSwNP-alone versus control and CRSwNP+AS versus control. **Table S3.** Top 50 DE-mRNAs of CRSwNP+AS versus CRSwNP-alone. **Table S4.** Expression of key cytokines and their receptors in nasal tissues from control subjects and CRSwNP-alone and CRSwNP+AS patients. **Table S5.** Top 50 DE-lncRNAs of CRSwNP+AS versus CRSwNP-alone. **Figure S1.** Correlation of infiltrating eosinophils and total IgE in nasal tissues of patients with CRSwNP. **Figure S2.** Differentially expressed mRNAs and differentially expressed lncRNAs in nasal tissues of CRSwNP patients. **Figure S3.** Hierarchical clustering of differentially expressed genes. **Figure S4.** GO biological processes enriched by common dysregulated genes in CRSwNP+AS and CRSwNP-alone. **FigureS5.** Gene number of modules identified by weighted gene co-expression network analysis. **Figure S6.** The expression and potential functions of LINC01146. **Figure S7.** Gene modules identified by WGCNA based on expression of DE-mRNAs and DE-lncRNAs in CRSwNP+AS versus CRSwNP-alone. **Figure S8.** Asthma related genes expressed in nasal tissue of patients with CRSwNP+AS and patients with CRSwNP-alone.

## Data Availability

The complete dataset is included in this manuscript.

## Abbreviations

CRS: Chronic rhinosinusitis
CRSwNP: Chronic rhinosinusitis with nasal polyps
CRSwNP + AS: Chronic Rhinosinusitis with Nasal Polyps with Comorbid Asthma
IL: Interleukin
lncRNA: Long non-coding RNA
DE: Differentially expressed
FPKM: Fragments per kilo-base of exon per million fragments mapped
WGCNA: Weighted gene coexpression network analysis
KEGG: Kyoto Encyclopedia of Genes and Genomes
