# Supplementary material for: Distinct type 2-high inflammation associated molecular signatures of chronic rhinosinusitis with nasal polyps with comorbid asthma
Source: Clin Transl Allergy. 2020 Jul 3;10:26. doi: 10.1186/s13601-020-00332-z (PMC7333405; doi:10.1186/s13601-020-00332-z)
Supplement: Supplementary file 1 — Additional file 1: Table S1. Demographic and clinical characteristics of study subjects. Table S2. Common DE-mRNAs and DE-lncRNAs shared by CRSwNP-alone versus control and CRSwNP+AS versus control. Table S3. Top 50 DE-mRNAs of CRSwNP+AS versus CRSwNP-alone. Table S4. Expression of key cytokines and their receptors in nasal tissues from control subjects and CRSwNP-alone and CRSwNP+AS patients. Table S5. Top 50 DE-lncRNAs of CRSwNP+AS versus CRSwNP-alone. Figure S1. Correlation of infiltrating eosinophils and total IgE in nasal tissues of patients with CRSwNP. Figure S2. Differentially expressed mRNAs and differentially expressed lncRNAs in nasal tissues of CRSwNP patients. Figure S3. Hierarchical clustering of differentially expressed genes. Figure S4. GO biological processes enriched by common dysregulated genes in CRSwNP+AS and CRSwNP-alone. FigureS5. Gene number of modules identified by weighted gene co-expression network analysis. Figure S6. The expression and potential functions of LINC01146. Figure S7. Gene modules identified by WGCNA based on expression of DE-mRNAs and DE-lncRNAs in CRSwNP+AS versus CRSwNP-alone. Figure S8. Asthma related genes expressed in nasal tissue of patients with CRSwNP+AS and patients with CRSwNP-alone. [file 13601_2020_332_MOESM1_ESM.docx]

**Additional file 1**

**Figure S1** Correlation of infiltrating eosinophils and total IgE in nasal tissues of patients with CRSwNP. The percentage of infiltrating eosinophils and concentrations of total IgE were assessed in nasal tissues of CRSwNP (n=17) and correlations were analyzed by Spearman correlation. CRSwNP: chronic rhinosinusitis with nasal polyps.


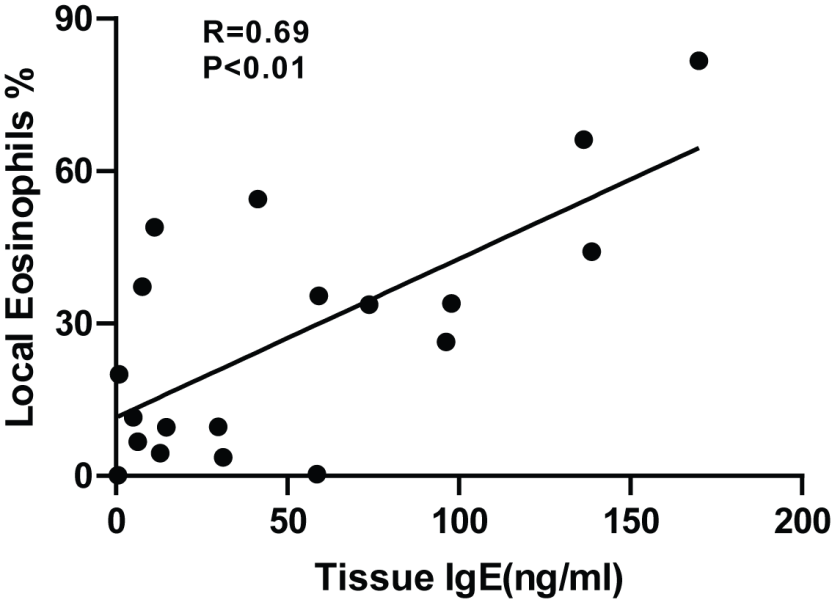


**Figure S2:** Differentially expressed mRNAs (DE-mRNAs) and differentially expressed lncRNAs (DE-lncRNAs) in nasal tissues of CRSwNP patients. (A-B) Venn diagrams depicting significant DE-mRNAs and DE-lncRNAs identified by RNA sequencing in CRSwNP with comorbid asthma (CRSwNP+AS) (n=10), CRSwNP-alone (n=10), and control (Ctrl) (n=9) subjects. The number of DE-mRNAs or DE-lncRNAs is shown in the corresponding areas. CRSwNP: chronic rhinosinusitis with nasal polyps; AS: asthma; lncRNA: long non-coding RNA; DE: differentially expressed.


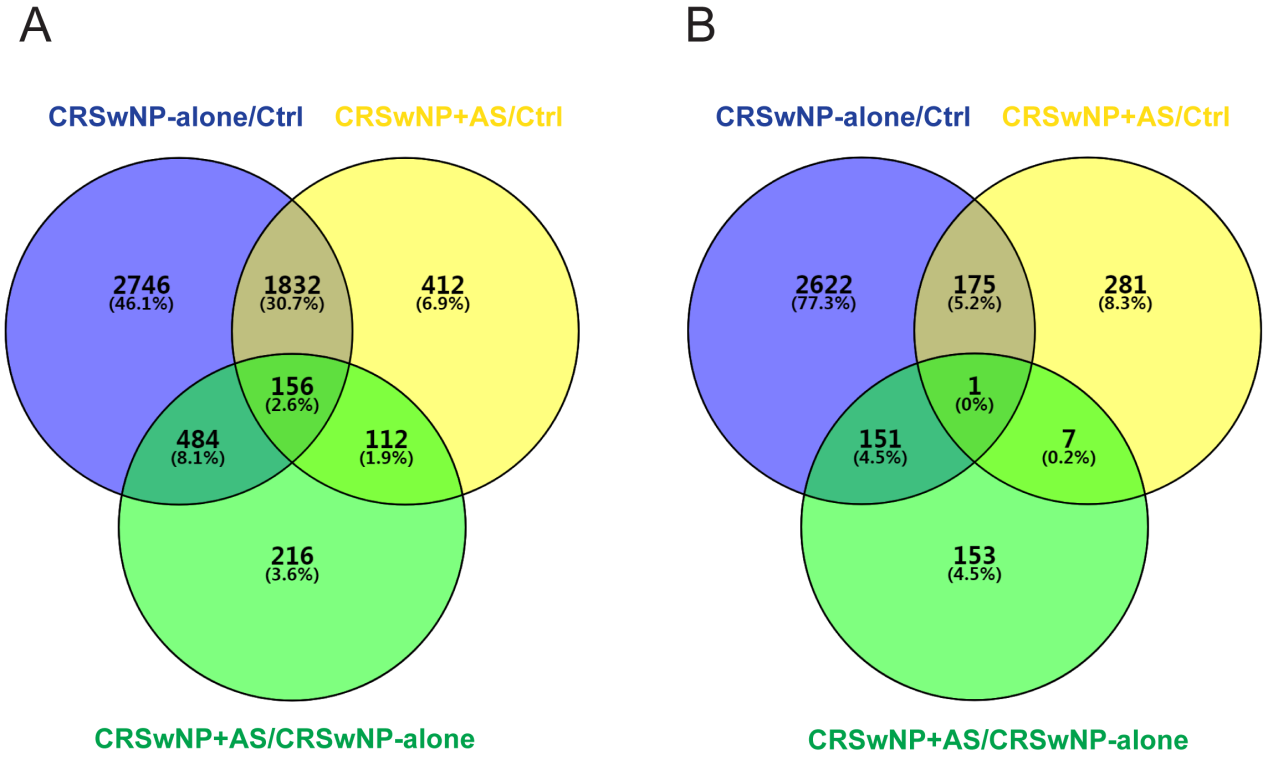


**Figure S3:** Hierarchical clustering of differentially expressed genes. Heat map showing top 500 differentially expressed genes. Background factors (gender, smoking, atopy, disease condition) of subjects are marked in the corresponding areas. CRSwNP+AS (n=10), CRSwNP-alone (n=10), and control (n=9). CRSwNP: chronic rhinosinusitis with nasal polyps; AS: asthma.

**
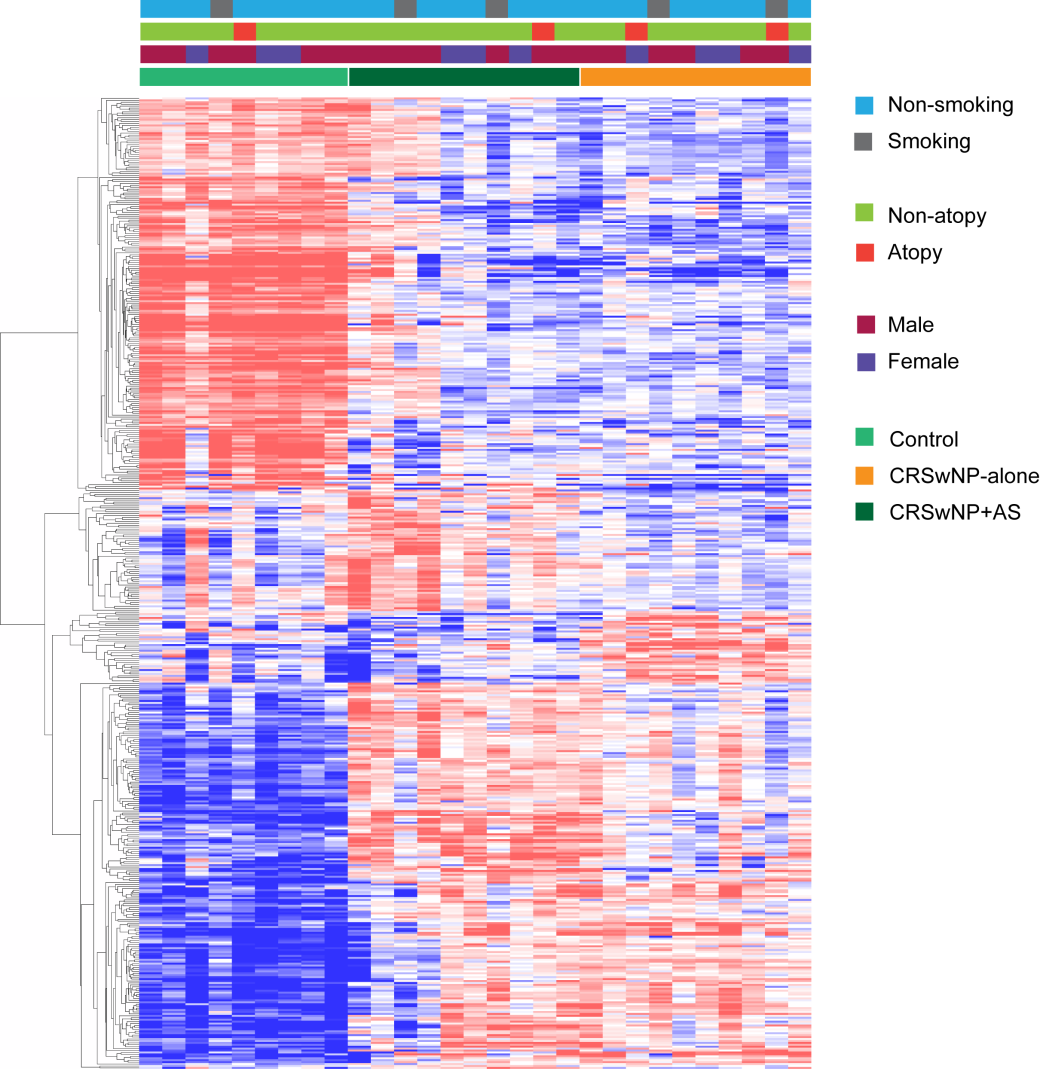
**

**Figure S4** GO biological processes enriched by common dysregulated genes in CRSwNP with comorbid asthma (CRSwNP+AS) and CRSwNP-alone. The result was visualized by cytoscape network. Node size represented gene number in node and node filled color represented P value. CRSwNP: chronic rhinosinusitis with nasal polyps; AS: asthma; GO: gene ontology.


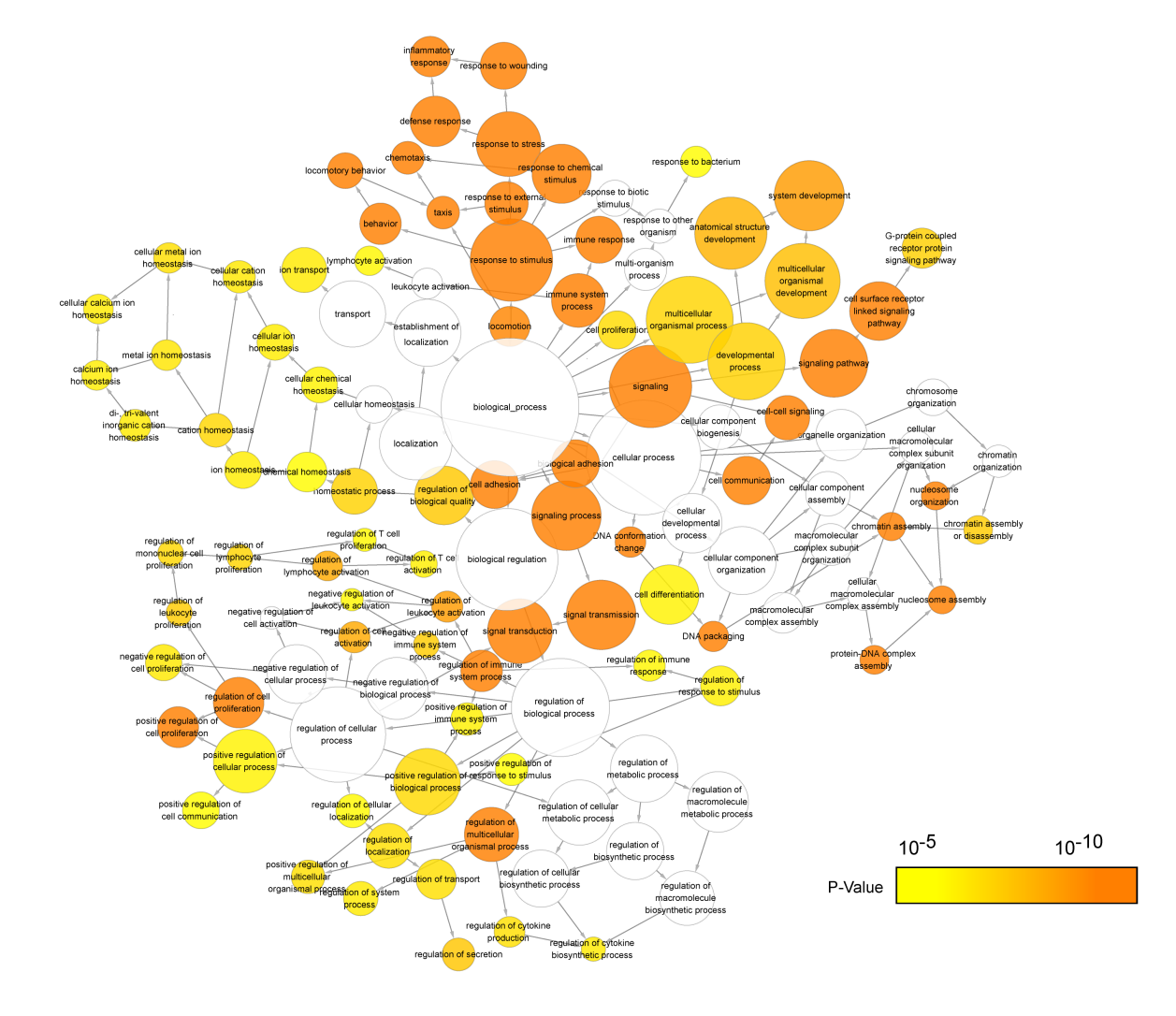


**Figure S5** Gene number of modules identified by weighted gene co-expression network analysis (WGCNA). WGCNA was applied to explore the potential functions of 176 common DE-lncRNAs, based on a coexpression network of DE-lncRNAs and DE-mRNAs. Branches of the dendrogram obtained by hierarchical clustering of adjacency based similarity result in 9 modules, labelled with distinct colours. Gene number of each module is shown above the corresponding column. lncRNA: long non-coding RNA; DE: differentially expressed.


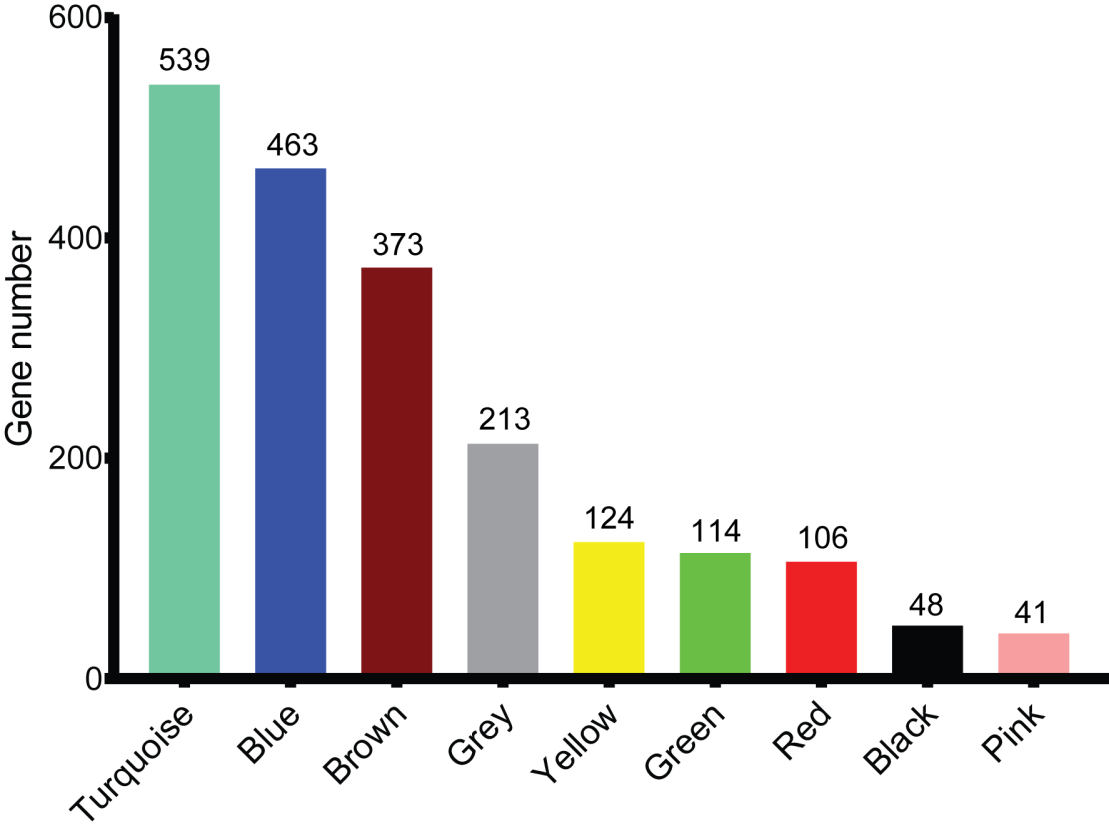


**Figure S6** The expression and potential functions of LINC01146. (A) The expression of LINC01146 in CRSwNP with comorbid asthma (CRSwNP+AS), CRSwNP-alone and control group. ** *P*<0.01. (B) LINC01146 gene locus and adjacent genes. Spearman correlation analysis was performed between expression of LINC01146 and GALC. (C) Top 10 KEGG pathways significantly enriched by coexpressed mRNAs of LINC01146. Values of *P*<0.05 were considered statistically significant. CRSwNP: chronic rhinosinusitis with nasal polyps; AS: asthma.


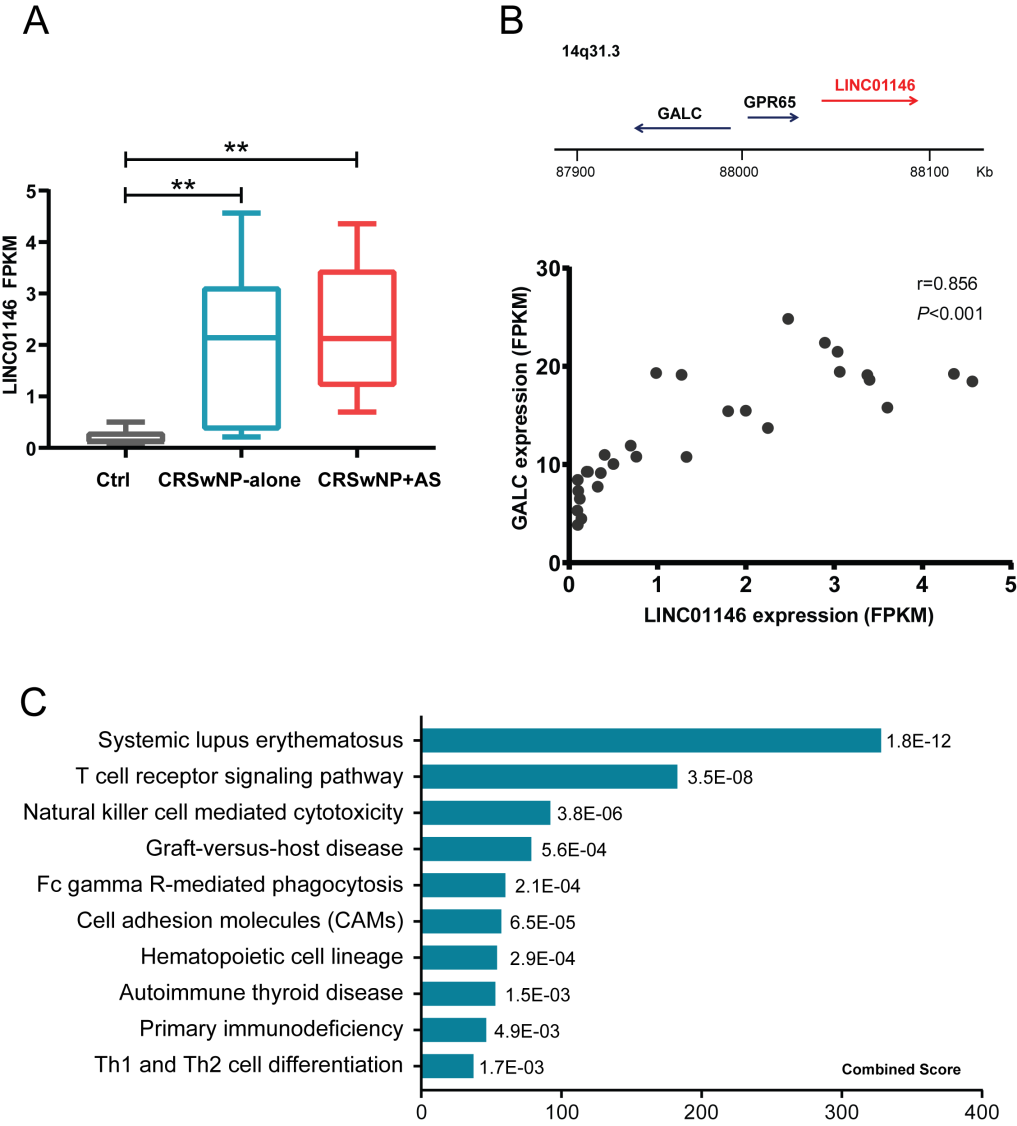


**Figure S7** Gene modules identified by weighted gene co-expression network analysis (WGCNA) based on expression of differentially expressed mRNAs (DE-mRNAs) and differentially expressed lncRNAs (DE-lncRNAs) in CRSwNP with comorbid asthma (CRSwNP+AS) versus CRSwNP-alone. Branches of the dendrogram obtained by hierarchical clustering of adjacency based similarity demonstrated 7 modules, labelled with distinct colours. CRSwNP: chronic rhinosinusitis with nasal polyps; AS: asthma; lncRNA: long non-coding RNA; DE: differentially expressed.


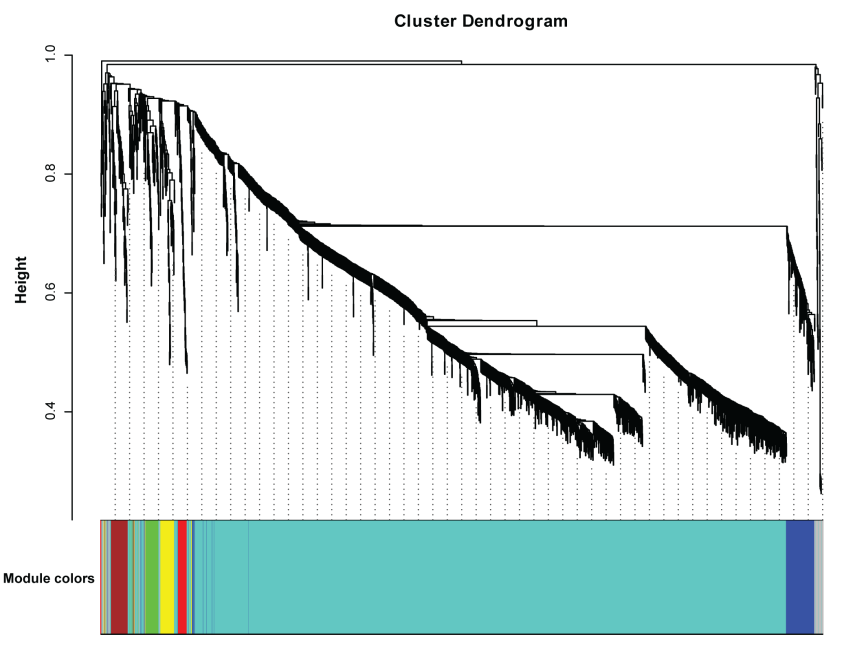


**Figure S8** Asthma related genes expressed in nasal tissue of CRSwNP patients with comorbid asthma (CRSwNP+AS) and patients with CRSwNP-alone. CRSwNP+AS (n=10), CRSwNP-alone (n=10), and control (n=9). CRSwNP: chronic rhinosinusitis with nasal polyps; AS: asthma; FPKM: Fragments per kilo-base of exon per million fragments mapped.


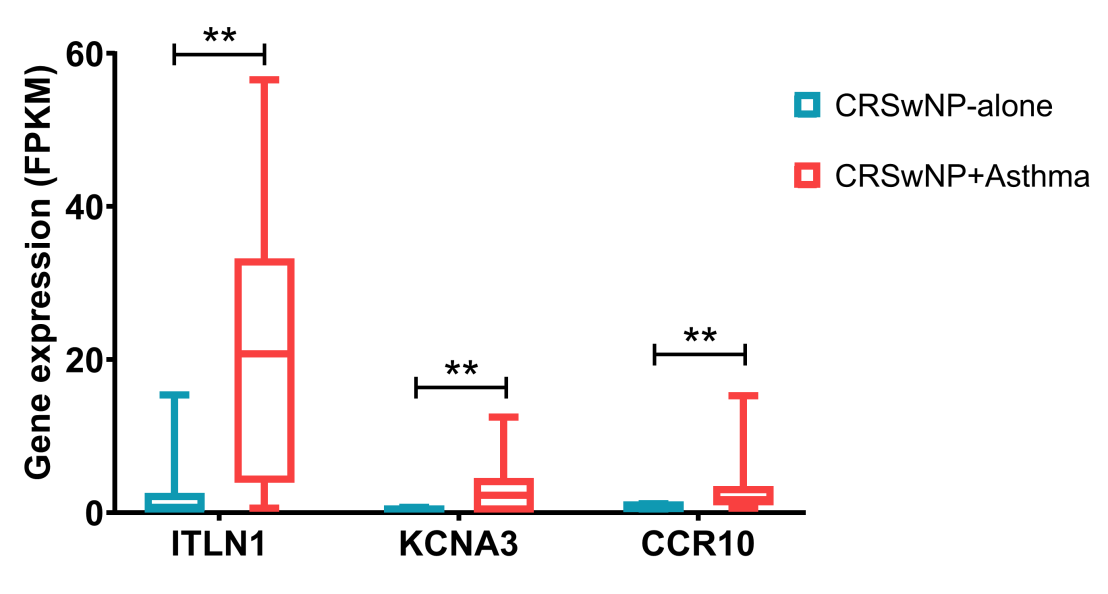


**Table S1.** Demographic and clinical characteristics of study subjects

|  | **Control** | **CRSwNP-alone** | **CRSwNP + AS** | ***P* value** |
| --- | --- | --- | --- | --- |
| **Subjects** | 31 | 99 | 65 | NA |
| **Sex (male/female)** | 16/15 | 61/38 | 36/29 | 0.427 |
| **Age (years±SD)** | 49.10±13.82 | 44.65±11.72 | 45.60±11.27 | 0.603 |
| **Atopy (yes/no)** | 5/26 | 22/77 | 25/40* | 0.025 |
| **Smoker (yes/no)** | 7/24 | 23/76 | 14/51 | 0.796 |
| **Recurrence (yes/no)** | NA | 21/78 | 26/39* | 0.009 |
| **FEV1/FVC%, median (IQR)** | NA | 88.65 (84.14-90.96) | 80.19 (68.53-85.41)* | <0.001 |
| **FENO, median (IQR)** | NA | 17.00 (12.25-23.00) | 33.50 (19.00-57.00)* | <0.001 |
| **Peripheral blood eosinophils (%), median (IQR)** | 1.70 (1.10-2.30) | 3.00 (1.50-4.90) | 6.45 (4.08-8.80)* | <0.001 |
| **Peripheral blood total IgE (kU/l), median (IQR)** | 14.40 (22.50-60.80) | 53.90 (22.37-90.88) | 143.00 (73.15-308.0)* | <0.001 |
| **Tissue eosinophils (%),**  **median (IQR)** | NA | 9.58 (3.18-34.55) | 34.78 (26.34-54.45)* | 0.002 |
| **Tissue neutrophils (%),**  **median (IQR)** | NA | 10.16 (2.75-16.22) | 7.21 (1.58-16.34) | 0.425 |
| **Tissue lymphocytes (%),**  **median (IQR)** | NA | 56.42 (32.49-69.23) | 38.79 (19.20-45.06)* | 0.037 |
| **Tissue plasma cells (%),**  **median (IQR)** | NA | 12.75 (7.95-21.26) | 16.54 (12.74-21.81) | 0.515 |
| **Tissue total IgE(ng/ml),**  **median (IQR)** | 3.48 (2.03-8.64) | 9.47 (4.63-19.98) | 90.98 (39.91-137.55)* | <0.001 |

* CRSwNP+AS vs CRSwNP-alone. CRSwNP: chronic rhinosinusitis with nasal polyps; AS: asthma.

**Table S2.** Common DE-mRNAs and DE-lncRNAs shared by CRSwNP-alone versus control and CRSwNP+AS versus control

| **Gene identifier** | **Gene name** | **Gene type** | **CRSwNP-alone vs. Control** | | **CRSwNP+AS vs. Control** | |
| --- | --- | --- | --- | --- | --- | --- |
|  |  |  | **Log2(Fold Change)** | ***P* value** | **Log2(Fold Change)** | ***P* value** |
| ENSG00000000938 | FGR | mRNA | 1.363 | 2.991E-07 | 2.002 | 1.330E-12 |
| ENSG00000002587 | HS3ST1 | mRNA | 1.320 | 5.218E-10 | 1.194 | 2.934E-07 |
| ENSG00000002726 | AOC1 | mRNA | 2.582 | 2.067E-05 | 2.715 | 1.269E-05 |
| ENSG00000002933 | TMEM176A | mRNA | 1.542 | 2.176E-13 | 1.642 | 1.457E-12 |
| ENSG00000003249 | DBNDD1 | mRNA | -2.091 | 1.412E-15 | -1.830 | 2.289E-10 |
| ENSG00000004468 | CD38 | mRNA | 1.502 | 1.786E-07 | 1.591 | 1.429E-04 |
| ENSG00000004660 | CAMKK1 | mRNA | -1.914 | 9.762E-17 | -1.172 | 1.212E-05 |
| ENSG00000004848 | ARX | mRNA | -2.866 | 1.009E-04 | -2.716 | 3.124E-04 |
| ENSG00000005059 | CCDC109B | mRNA | 1.647 | 2.449E-12 | 1.184 | 6.296E-04 |
| ENSG00000005379 | BZRAP1 | mRNA | -1.948 | 1.154E-13 | -1.047 | 1.458E-03 |
| ENSG00000005513 | SOX8 | mRNA | -4.699 | 6.168E-43 | -3.084 | 6.873E-12 |
| ENSG00000005981 | ASB4 | mRNA | -1.866 | 1.121E-04 | -1.500 | 1.084E-03 |
| ENSG00000006071 | ABCC8 | mRNA | -2.735 | 2.813E-09 | -2.024 | 4.787E-04 |
| ENSG00000006074 | CCL18 | mRNA | 6.938 | 2.438E-12 | 8.880 | 5.284E-38 |
| ENSG00000006128 | TAC1 | mRNA | -2.891 | 5.193E-05 | -2.233 | 4.927E-03 |
| ENSG00000006377 | DLX6 | mRNA | -1.314 | 1.458E-03 | -1.520 | 1.202E-03 |
| ENSG00000006576 | PHTF2 | mRNA | 1.753 | 1.203E-08 | 1.244 | 1.834E-03 |
| ENSG00000006740 | ARHGAP44 | mRNA | -1.439 | 8.561E-08 | -1.676 | 7.124E-11 |
| ENSG00000007216 | SLC13A2 | mRNA | -4.510 | 5.905E-46 | -3.832 | 1.854E-16 |
| ENSG00000007314 | SCN4A | mRNA | -2.535 | 2.775E-12 | -2.756 | 1.508E-09 |
| ENSG00000007516 | BAIAP3 | mRNA | -1.887 | 2.231E-04 | -1.986 | 5.195E-05 |
| ENSG00000007968 | E2F2 | mRNA | 1.821 | 2.022E-05 | 1.966 | 4.043E-06 |
| ENSG00000008283 | CYB561 | mRNA | -1.678 | 1.704E-15 | -1.630 | 3.489E-13 |
| ENSG00000008441 | NFIX | mRNA | -1.903 | 9.730E-24 | -1.456 | 5.071E-08 |
| ENSG00000008516 | MMP25 | mRNA | 1.553 | 3.733E-05 | 2.522 | 2.782E-08 |
| ENSG00000008735 | MAPK8IP2 | mRNA | -2.214 | 3.098E-13 | -1.312 | 5.158E-04 |
| ENSG00000009694 | TENM1 | mRNA | -1.790 | 3.207E-04 | -2.639 | 1.646E-06 |
| ENSG00000009790 | TRAF3IP3 | mRNA | 1.452 | 2.909E-06 | 1.352 | 3.834E-05 |
| ENSG00000010610 | CD4 | mRNA | 1.282 | 3.592E-07 | 1.740 | 6.099E-10 |
| ENSG00000011347 | SYT7 | mRNA | -2.528 | 1.340E-10 | -2.657 | 2.325E-11 |
| ENSG00000011426 | ANLN | mRNA | 2.990 | 1.226E-14 | 2.356 | 3.240E-07 |
| ENSG00000011600 | TYROBP | mRNA | 2.102 | 3.467E-16 | 2.386 | 1.772E-17 |
| ENSG00000012223 | LTF | mRNA | -5.519 | 4.364E-32 | -4.285 | 1.084E-11 |
| ENSG00000012660 | ELOVL5 | mRNA | 1.381 | 7.457E-09 | 1.399 | 3.331E-06 |
| ENSG00000012779 | ALOX5 | mRNA | 1.197 | 5.690E-08 | 1.668 | 1.484E-10 |
| ENSG00000015285 | WAS | mRNA | 1.317 | 6.102E-07 | 1.644 | 4.939E-08 |
| ENSG00000016391 | CHDH | mRNA | -2.514 | 2.192E-22 | -1.529 | 6.757E-08 |
| ENSG00000016490 | CLCA1 | mRNA | 4.994 | 6.521E-03 | 8.494 | 1.837E-10 |
| ENSG00000017373 | SRCIN1 | mRNA | -2.694 | 2.189E-14 | -2.286 | 5.552E-10 |
| ENSG00000018280 | SLC11A1 | mRNA | 1.581 | 1.296E-03 | 1.963 | 2.199E-05 |
| ENSG00000018625 | ATP1A2 | mRNA | -1.712 | 1.086E-08 | -1.218 | 1.092E-04 |
| ENSG00000019169 | MARCO | mRNA | 3.442 | 4.480E-09 | 7.840 | 3.180E-15 |
| ENSG00000019505 | SYT13 | mRNA | -3.812 | 1.160E-10 | -3.814 | 8.564E-14 |
| ENSG00000021300 | PLEKHB1 | mRNA | -2.130 | 4.145E-11 | -1.657 | 4.254E-08 |
| ENSG00000023445 | BIRC3 | mRNA | 1.512 | 7.837E-06 | 1.804 | 3.521E-04 |
| ENSG00000024526 | DEPDC1 | mRNA | 3.470 | 6.694E-12 | 2.725 | 1.083E-04 |
| ENSG00000026103 | FAS | mRNA | 1.794 | 1.612E-12 | 1.251 | 8.038E-04 |
| ENSG00000026508 | CD44 | mRNA | 1.802 | 2.109E-09 | 1.758 | 3.464E-06 |
| ENSG00000026751 | SLAMF7 | mRNA | 1.426 | 4.517E-05 | 1.324 | 9.837E-04 |
| ENSG00000029153 | ARNTL2 | mRNA | 1.924 | 9.384E-19 | 1.612 | 1.602E-08 |
| ENSG00000029559 | IBSP | mRNA | -4.158 | 5.171E-05 | -3.140 | 4.729E-03 |
| ENSG00000029993 | HMGB3 | mRNA | 1.300 | 1.302E-08 | 1.069 | 1.485E-04 |
| ENSG00000034063 | UHRF1 | mRNA | 1.666 | 7.620E-05 | 1.296 | 1.491E-03 |
| ENSG00000035499 | DEPDC1B | mRNA | 2.913 | 1.450E-10 | 2.530 | 9.649E-07 |
| ENSG00000037749 | MFAP3 | mRNA | 1.336 | 4.480E-07 | 1.071 | 4.651E-03 |
| ENSG00000038427 | VCAN | mRNA | 1.556 | 3.348E-09 | 1.184 | 2.950E-04 |
| ENSG00000038945 | MSR1 | mRNA | 2.761 | 2.462E-16 | 2.410 | 3.161E-12 |
| ENSG00000041515 | MYO16 | mRNA | 3.342 | 3.981E-13 | 1.884 | 2.325E-04 |
| ENSG00000043039 | BARX2 | mRNA | -3.892 | 3.110E-23 | -3.190 | 9.519E-11 |
| ENSG00000043462 | LCP2 | mRNA | 2.326 | 1.943E-14 | 2.292 | 8.401E-11 |
| ENSG00000043591 | ADRB1 | mRNA | -1.214 | 9.870E-04 | -1.136 | 6.207E-03 |
| ENSG00000044524 | EPHA3 | mRNA | -1.995 | 6.260E-07 | -1.922 | 3.654E-05 |
| ENSG00000048028 | USP28 | mRNA | 1.104 | 6.469E-07 | 1.206 | 9.301E-05 |
| ENSG00000048540 | LMO3 | mRNA | -1.489 | 1.529E-02 | -1.868 | 2.132E-03 |
| ENSG00000048740 | CELF2 | mRNA | 1.256 | 1.417E-05 | 1.284 | 2.157E-04 |
| ENSG00000049089 | COL9A2 | mRNA | -2.404 | 5.416E-17 | -1.485 | 3.712E-04 |
| ENSG00000049130 | KITLG | mRNA | 1.591 | 1.341E-09 | 1.165 | 8.655E-04 |
| ENSG00000049192 | ADAMTS6 | mRNA | 1.462 | 1.401E-04 | 1.364 | 9.474E-04 |
| ENSG00000049768 | FOXP3 | mRNA | 2.268 | 1.465E-10 | 1.568 | 1.303E-04 |
| ENSG00000051180 | RAD51 | mRNA | 1.630 | 3.170E-05 | 1.065 | 6.428E-03 |
| ENSG00000052344 | PRSS8 | mRNA | -2.359 | 1.382E-22 | -2.084 | 1.673E-10 |
| ENSG00000054179 | ENTPD2 | mRNA | -1.438 | 5.814E-06 | -1.537 | 2.124E-07 |
| ENSG00000054277 | OPN3 | mRNA | 1.112 | 1.749E-05 | 1.087 | 7.740E-04 |
| ENSG00000054803 | CBLN4 | mRNA | -1.874 | 3.646E-03 | -2.306 | 8.354E-04 |
| ENSG00000057149 | SERPINB3 | mRNA | 2.646 | 1.878E-24 | 1.609 | 1.912E-05 |
| ENSG00000057294 | PKP2 | mRNA | -1.728 | 7.620E-17 | -1.559 | 9.081E-09 |
| ENSG00000058453 | CROCC | mRNA | -2.541 | 4.145E-08 | -1.446 | 4.468E-03 |
| ENSG00000059377 | TBXAS1 | mRNA | 1.433 | 3.547E-09 | 2.104 | 9.288E-19 |
| ENSG00000059573 | ALDH18A1 | mRNA | -1.378 | 1.462E-11 | -1.302 | 8.065E-09 |
| ENSG00000059728 | MXD1 | mRNA | 1.542 | 4.348E-10 | 1.992 | 2.009E-09 |
| ENSG00000064042 | LIMCH1 | mRNA | -1.533 | 4.268E-11 | -1.363 | 1.571E-07 |
| ENSG00000064225 | ST3GAL6 | mRNA | -1.221 | 6.922E-05 | -1.516 | 3.166E-05 |
| ENSG00000064300 | NGFR | mRNA | -1.226 | 2.518E-05 | -1.278 | 1.037E-04 |
| ENSG00000064651 | SLC12A2 | mRNA | -2.204 | 4.507E-13 | -2.461 | 8.387E-14 |
| ENSG00000064886 | CHI3L2 | mRNA | -2.193 | 2.547E-07 | -3.003 | 2.674E-13 |
| ENSG00000065054 | SLC9A3R2 | mRNA | -1.921 | 4.396E-11 | -1.021 | 4.493E-03 |
| ENSG00000065328 | MCM10 | mRNA | 2.908 | 1.191E-08 | 2.268 | 7.293E-06 |
| ENSG00000065371 | ROPN1 | mRNA | -5.602 | 6.384E-13 | -6.016 | 6.583E-13 |
| ENSG00000065615 | CYB5R4 | mRNA | 1.418 | 7.985E-10 | 1.056 | 4.812E-04 |
| ENSG00000065675 | PRKCQ | mRNA | 1.110 | 6.439E-05 | 1.004 | 1.670E-04 |
| ENSG00000066032 | CTNNA2 | mRNA | -2.464 | 1.443E-06 | -2.979 | 3.676E-08 |
| ENSG00000066230 | SLC9A3 | mRNA | 1.337 | 4.553E-04 | 2.245 | 1.539E-08 |
| ENSG00000066279 | ASPM | mRNA | 3.411 | 1.749E-16 | 2.812 | 1.141E-07 |
| ENSG00000066294 | CD84 | mRNA | 2.365 | 1.155E-15 | 2.534 | 2.690E-17 |
| ENSG00000066336 | SPI1 | mRNA | 1.586 | 6.217E-08 | 2.182 | 2.245E-11 |
| ENSG00000066468 | FGFR2 | mRNA | -1.295 | 1.229E-11 | -1.149 | 3.325E-06 |
| ENSG00000067798 | NAV3 | mRNA | -1.022 | 1.040E-03 | -1.311 | 5.659E-04 |
| ENSG00000068366 | ACSL4 | mRNA | 1.805 | 1.546E-09 | 1.580 | 4.918E-05 |
| ENSG00000068489 | PRR11 | mRNA | 1.375 | 4.777E-05 | 1.289 | 7.064E-04 |
| ENSG00000069431 | ABCC9 | mRNA | 1.809 | 1.914E-16 | 1.334 | 3.081E-06 |
| ENSG00000069702 | TGFBR3 | mRNA | -1.308 | 2.278E-11 | -1.266 | 1.525E-08 |
| ENSG00000070190 | DAPP1 | mRNA | 1.725 | 3.368E-12 | 1.218 | 6.595E-04 |
| ENSG00000070193 | FGF10 | mRNA | -1.071 | 1.180E-03 | -1.922 | 1.764E-07 |
| ENSG00000070985 | TRPM5 | mRNA | -1.494 | 8.113E-03 | 2.021 | 4.670E-05 |
| ENSG00000071991 | CDH19 | mRNA | -5.670 | 3.292E-10 | -6.450 | 2.472E-10 |
| ENSG00000072041 | SLC6A15 | mRNA | 1.253 | 5.635E-05 | 1.194 | 4.999E-03 |
| ENSG00000072401 | UBE2D1 | mRNA | 1.830 | 1.439E-11 | 1.097 | 3.990E-03 |
| ENSG00000072571 | HMMR | mRNA | 3.836 | 4.705E-15 | 2.880 | 3.011E-06 |
| ENSG00000072694 | FCGR2B | mRNA | 1.437 | 1.270E-08 | 1.967 | 1.777E-09 |
| ENSG00000073282 | TP63 | mRNA | 1.790 | 1.290E-14 | 1.606 | 1.012E-05 |
| ENSG00000074047 | GLI2 | mRNA | -1.968 | 2.181E-13 | -1.042 | 5.615E-04 |
| ENSG00000074370 | ATP2A3 | mRNA | -3.738 | 1.516E-40 | -2.670 | 4.754E-14 |
| ENSG00000074416 | MGLL | mRNA | -2.509 | 1.814E-35 | -1.650 | 1.426E-10 |
| ENSG00000074966 | TXK | mRNA | 2.062 | 1.392E-06 | 1.508 | 4.051E-04 |
| ENSG00000075218 | GTSE1 | mRNA | 1.920 | 4.452E-06 | 1.876 | 2.004E-05 |
| ENSG00000075290 | WNT8B | mRNA | -1.677 | 2.535E-03 | -1.715 | 4.475E-03 |
| ENSG00000075884 | ARHGAP15 | mRNA | 1.738 | 1.308E-07 | 1.417 | 4.347E-04 |
| ENSG00000076382 | SPAG5 | mRNA | 1.364 | 2.979E-07 | 1.024 | 1.051E-03 |
| ENSG00000076641 | PAG1 | mRNA | 1.430 | 3.279E-09 | 1.325 | 1.025E-06 |
| ENSG00000076826 | CAMSAP3 | mRNA | -2.215 | 7.524E-21 | -1.483 | 1.438E-05 |
| ENSG00000076864 | RAP1GAP | mRNA | -4.209 | 6.673E-51 | -2.703 | 1.872E-25 |
| ENSG00000077152 | UBE2T | mRNA | 3.028 | 1.376E-14 | 2.164 | 4.315E-05 |
| ENSG00000077264 | PAK3 | mRNA | -1.044 | 3.681E-04 | -1.091 | 1.170E-03 |
| ENSG00000077279 | DCX | mRNA | -4.441 | 1.909E-12 | -4.925 | 4.718E-09 |
| ENSG00000077420 | APBB1IP | mRNA | 1.980 | 5.385E-13 | 1.872 | 1.683E-09 |
| ENSG00000077585 | GPR137B | mRNA | 1.737 | 6.552E-11 | 1.575 | 2.023E-06 |
| ENSG00000077984 | CST7 | mRNA | 1.258 | 1.367E-05 | 1.182 | 1.250E-04 |
| ENSG00000078053 | AMPH | mRNA | 1.261 | 6.081E-06 | 1.053 | 1.526E-04 |
| ENSG00000078081 | LAMP3 | mRNA | 2.146 | 1.002E-10 | 1.789 | 3.001E-03 |
| ENSG00000078098 | FAP | mRNA | 3.541 | 3.185E-14 | 3.532 | 4.293E-12 |
| ENSG00000078237 | C12orf5 | mRNA | 1.685 | 1.974E-13 | 1.228 | 4.987E-05 |
| ENSG00000078549 | ADCYAP1R1 | mRNA | -3.294 | 1.106E-11 | -1.495 | 2.165E-03 |
| ENSG00000078589 | P2RY10 | mRNA | 2.413 | 2.992E-08 | 1.980 | 3.405E-04 |
| ENSG00000078898 | BPIFB2 | mRNA | -8.633 | 3.512E-32 | -6.916 | 1.198E-19 |
| ENSG00000079101 | CLUL1 | mRNA | -2.224 | 3.228E-07 | -2.048 | 3.294E-06 |
| ENSG00000079215 | SLC1A3 | mRNA | 1.089 | 3.220E-04 | 1.639 | 8.565E-05 |
| ENSG00000079263 | SP140 | mRNA | 1.562 | 1.904E-05 | 1.126 | 5.630E-03 |
| ENSG00000079337 | RAPGEF3 | mRNA | -2.522 | 1.564E-34 | -1.377 | 1.857E-05 |
| ENSG00000079691 | LRRC16A | mRNA | -1.545 | 2.155E-07 | -1.950 | 1.084E-08 |
| ENSG00000080031 | PTPRH | mRNA | 2.384 | 5.746E-11 | 1.617 | 1.509E-04 |
| ENSG00000080224 | EPHA6 | mRNA | -4.529 | 2.787E-23 | -2.569 | 2.917E-04 |
| ENSG00000080986 | NDC80 | mRNA | 3.094 | 6.350E-11 | 2.161 | 7.327E-05 |
| ENSG00000081148 | IMPG2 | mRNA | -1.697 | 2.065E-02 | -2.038 | 5.579E-03 |
| ENSG00000081237 | PTPRC | mRNA | 2.578 | 4.108E-12 | 2.026 | 1.516E-05 |
| ENSG00000081479 | LRP2 | mRNA | 1.142 | 4.070E-05 | 1.304 | 4.626E-04 |
| ENSG00000081760 | AACS | mRNA | -2.043 | 7.550E-24 | -1.373 | 1.499E-08 |
| ENSG00000081803 | CADPS2 | mRNA | -1.321 | 5.824E-12 | -1.156 | 1.351E-07 |
| ENSG00000081985 | IL12RB2 | mRNA | 2.107 | 8.786E-10 | 1.237 | 1.519E-03 |
| ENSG00000082074 | FYB | mRNA | 2.688 | 1.371E-15 | 2.062 | 1.626E-06 |
| ENSG00000082126 | MPP4 | mRNA | 1.264 | 5.287E-04 | 1.372 | 2.153E-03 |
| ENSG00000082482 | KCNK2 | mRNA | -1.229 | 6.133E-04 | -1.544 | 4.512E-04 |
| ENSG00000083454 | P2RX5 | mRNA | 1.364 | 3.013E-04 | 1.584 | 4.674E-05 |
| ENSG00000084110 | HAL | mRNA | 1.916 | 1.421E-07 | 1.894 | 1.577E-05 |
| ENSG00000084453 | SLCO1A2 | mRNA | -5.647 | 1.005E-15 | -2.628 | 1.697E-05 |
| ENSG00000084628 | NKAIN1 | mRNA | -2.097 | 3.273E-04 | -2.563 | 1.645E-05 |
| ENSG00000085117 | CD82 | mRNA | -1.302 | 3.244E-09 | -1.148 | 3.208E-05 |
| ENSG00000085265 | FCN1 | mRNA | 2.616 | 6.461E-10 | 2.041 | 3.333E-05 |
| ENSG00000085514 | PILRA | mRNA | 1.579 | 1.004E-06 | 1.729 | 8.937E-09 |
| ENSG00000085840 | ORC1 | mRNA | 1.675 | 1.431E-05 | 1.159 | 3.742E-03 |
| ENSG00000086159 | AQP6 | mRNA | -1.924 | 2.329E-09 | -1.069 | 3.663E-03 |
| ENSG00000086205 | FOLH1 | mRNA | -3.260 | 1.675E-17 | -3.364 | 6.238E-18 |
| ENSG00000086300 | SNX10 | mRNA | 2.849 | 3.158E-10 | 2.537 | 8.189E-05 |
| ENSG00000086696 | HSD17B2 | mRNA | -1.842 | 1.695E-13 | -1.574 | 4.960E-08 |
| ENSG00000086991 | NOX4 | mRNA | 3.211 | 2.822E-19 | 3.092 | 5.794E-12 |
| ENSG00000087128 | TMPRSS11E | mRNA | 2.627 | 8.981E-06 | 2.226 | 4.358E-04 |
| ENSG00000087253 | LPCAT2 | mRNA | 1.426 | 1.077E-06 | 1.498 | 1.062E-05 |
| ENSG00000087494 | PTHLH | mRNA | 2.690 | 4.625E-16 | 2.742 | 6.521E-17 |
| ENSG00000087586 | AURKA | mRNA | 2.657 | 5.327E-11 | 2.010 | 2.448E-05 |
| ENSG00000087589 | CASS4 | mRNA | 2.098 | 4.469E-10 | 2.190 | 8.685E-09 |
| ENSG00000087916 | SLC6A14 | mRNA | 2.140 | 7.386E-13 | 1.655 | 3.276E-05 |
| ENSG00000088325 | TPX2 | mRNA | 2.293 | 1.887E-09 | 1.964 | 1.275E-05 |
| ENSG00000088386 | SLC15A1 | mRNA | 1.150 | 9.324E-03 | 1.460 | 7.246E-04 |
| ENSG00000088827 | SIGLEC1 | mRNA | 1.921 | 2.620E-09 | 3.081 | 1.859E-16 |
| ENSG00000088899 | LZTS3 | mRNA | -2.371 | 2.149E-29 | -1.622 | 2.994E-07 |
| ENSG00000088992 | TESC | mRNA | -1.825 | 6.713E-11 | -1.673 | 1.263E-08 |
| ENSG00000089199 | CHGB | mRNA | -1.730 | 1.407E-03 | -2.677 | 3.928E-06 |
| ENSG00000089220 | PEBP1 | mRNA | -1.089 | 3.210E-06 | -1.191 | 5.989E-06 |
| ENSG00000089250 | NOS1 | mRNA | 3.388 | 9.657E-05 | 4.564 | 4.092E-12 |
| ENSG00000089327 | FXYD5 | mRNA | 1.174 | 1.338E-07 | 1.117 | 2.387E-06 |
| ENSG00000089505 | CMTM1 | mRNA | 1.309 | 3.564E-08 | 1.288 | 8.181E-06 |
| ENSG00000089847 | ANKRD24 | mRNA | -2.081 | 4.563E-09 | -1.321 | 9.834E-04 |
| ENSG00000090382 | LYZ | mRNA | -5.416 | 1.731E-28 | -5.426 | 4.174E-21 |
| ENSG00000090512 | FETUB | mRNA | 2.852 | 5.145E-03 | 4.345 | 3.185E-07 |
| ENSG00000090565 | RAB11FIP3 | mRNA | -2.369 | 1.314E-25 | -1.282 | 8.651E-05 |
| ENSG00000090659 | CD209 | mRNA | 2.131 | 3.121E-08 | 3.198 | 9.301E-19 |
| ENSG00000090889 | KIF4A | mRNA | 2.268 | 8.006E-09 | 1.916 | 4.577E-05 |
| ENSG00000091106 | NLRC4 | mRNA | 2.545 | 1.762E-11 | 2.479 | 5.664E-08 |
| ENSG00000091137 | SLC26A4 | mRNA | 3.430 | 4.916E-09 | 2.237 | 4.672E-04 |
| ENSG00000091262 | ABCC6 | mRNA | -2.063 | 4.575E-06 | -1.867 | 1.266E-05 |
| ENSG00000091513 | TF | mRNA | -1.391 | 6.987E-03 | -2.903 | 1.306E-07 |
| ENSG00000092067 | CEBPE | mRNA | 3.345 | 9.085E-06 | 4.993 | 7.184E-14 |
| ENSG00000092096 | SLC22A17 | mRNA | -2.083 | 3.477E-18 | -1.160 | 2.518E-04 |
| ENSG00000092621 | PHGDH | mRNA | -3.648 | 4.074E-55 | -2.204 | 1.256E-10 |
| ENSG00000092853 | CLSPN | mRNA | 2.376 | 2.743E-08 | 1.824 | 1.804E-05 |
| ENSG00000093009 | CDC45 | mRNA | 2.407 | 2.724E-08 | 2.133 | 3.333E-06 |
| ENSG00000094755 | GABRP | mRNA | 2.160 | 3.339E-14 | 1.699 | 1.289E-07 |
| ENSG00000094963 | FMO2 | mRNA | 1.332 | 4.874E-07 | 1.115 | 5.754E-04 |
| ENSG00000095380 | NANS | mRNA | -1.380 | 1.458E-12 | -1.206 | 4.385E-09 |
| ENSG00000095585 | BLNK | mRNA | 1.522 | 8.429E-06 | 1.185 | 7.599E-03 |
| ENSG00000095637 | SORBS1 | mRNA | -1.226 | 7.505E-06 | -1.022 | 1.342E-03 |
| ENSG00000095713 | CRTAC1 | mRNA | -3.842 | 1.874E-13 | -3.687 | 1.290E-13 |
| ENSG00000095752 | IL11 | mRNA | 1.712 | 1.787E-03 | 2.787 | 2.501E-08 |
| ENSG00000095917 | TPSD1 | mRNA | 1.389 | 1.887E-02 | 2.149 | 3.540E-06 |
| ENSG00000096006 | CRISP3 | mRNA | -6.647 | 1.445E-10 | -4.948 | 1.984E-05 |
| ENSG00000097046 | CDC7 | mRNA | 1.944 | 3.603E-10 | 1.168 | 3.549E-03 |
| ENSG00000099840 | IZUMO4 | mRNA | -1.278 | 9.728E-05 | -1.019 | 5.817E-03 |
| ENSG00000099953 | MMP11 | mRNA | 2.374 | 5.988E-06 | 3.201 | 9.416E-09 |
| ENSG00000099985 | OSM | mRNA | 2.927 | 4.310E-07 | 3.653 | 2.582E-11 |
| ENSG00000100012 | SEC14L3 | mRNA | -4.054 | 1.761E-09 | -3.500 | 4.464E-08 |
| ENSG00000100055 | CYTH4 | mRNA | 1.255 | 9.381E-07 | 1.509 | 2.717E-07 |
| ENSG00000100077 | ADRBK2 | mRNA | 1.113 | 1.211E-09 | 1.052 | 1.431E-05 |
| ENSG00000100116 | GCAT | mRNA | -1.640 | 1.406E-10 | -1.252 | 3.772E-06 |
| ENSG00000100122 | CRYBB1 | mRNA | 1.226 | 2.103E-02 | 1.549 | 2.135E-03 |
| ENSG00000100146 | SOX10 | mRNA | -5.879 | 1.365E-40 | -3.828 | 2.474E-10 |
| ENSG00000100170 | SLC5A1 | mRNA | -3.461 | 1.765E-14 | -3.761 | 7.472E-16 |
| ENSG00000100219 | XBP1 | mRNA | -1.419 | 2.819E-09 | -1.504 | 3.572E-09 |
| ENSG00000100292 | HMOX1 | mRNA | 2.170 | 1.025E-08 | 2.322 | 1.992E-13 |
| ENSG00000100344 | PNPLA3 | mRNA | -1.713 | 1.179E-08 | -1.259 | 2.246E-04 |
| ENSG00000100365 | NCF4 | mRNA | 1.054 | 3.640E-05 | 1.996 | 1.103E-11 |
| ENSG00000100368 | CSF2RB | mRNA | 1.592 | 2.219E-10 | 2.066 | 8.793E-11 |
| ENSG00000100399 | CHADL | mRNA | -2.835 | 6.121E-18 | -1.582 | 1.899E-04 |
| ENSG00000100433 | KCNK10 | mRNA | 2.664 | 1.623E-05 | 2.763 | 3.106E-04 |
| ENSG00000100448 | CTSG | mRNA | 1.724 | 1.007E-04 | 1.977 | 1.150E-04 |
| ENSG00000100453 | GZMB | mRNA | 2.401 | 3.355E-06 | 1.988 | 2.658E-03 |
| ENSG00000100505 | TRIM9 | mRNA | -1.741 | 1.499E-08 | -1.172 | 1.622E-03 |
| ENSG00000100526 | CDKN3 | mRNA | 3.845 | 3.639E-10 | 3.308 | 5.968E-05 |
| ENSG00000100565 | C14orf166B | mRNA | -2.488 | 2.281E-03 | -2.700 | 3.884E-03 |
| ENSG00000100644 | HIF1A | mRNA | 1.756 | 2.986E-11 | 1.346 | 4.297E-05 |
| ENSG00000100739 | BDKRB1 | mRNA | 1.058 | 2.894E-03 | 1.542 | 4.844E-03 |
| ENSG00000100994 | PYGB | mRNA | -1.727 | 2.107E-14 | -1.048 | 6.769E-04 |
| ENSG00000101003 | GINS1 | mRNA | 1.758 | 3.050E-09 | 1.181 | 2.351E-04 |
| ENSG00000101057 | MYBL2 | mRNA | 1.706 | 1.179E-04 | 1.945 | 5.085E-05 |
| ENSG00000101098 | RIMS4 | mRNA | -4.100 | 6.011E-20 | -3.473 | 2.125E-07 |
| ENSG00000101162 | TUBB1 | mRNA | 1.560 | 2.011E-02 | 1.978 | 4.772E-03 |
| ENSG00000101188 | NTSR1 | mRNA | 1.931 | 7.651E-03 | 3.117 | 1.672E-04 |
| ENSG00000101203 | COL20A1 | mRNA | -2.673 | 2.612E-03 | -2.717 | 3.532E-04 |
| ENSG00000101204 | CHRNA4 | mRNA | -4.228 | 1.712E-06 | -3.211 | 9.714E-05 |
| ENSG00000101210 | EEF1A2 | mRNA | -3.716 | 6.169E-14 | -2.262 | 9.172E-08 |
| ENSG00000101265 | RASSF2 | mRNA | 1.060 | 2.999E-06 | 1.535 | 4.329E-08 |
| ENSG00000101276 | SLC52A3 | mRNA | -1.438 | 1.080E-08 | -1.429 | 5.747E-07 |
| ENSG00000101298 | SNPH | mRNA | -2.357 | 9.023E-16 | -1.275 | 1.805E-03 |
| ENSG00000101307 | SIRPB1 | mRNA | 2.842 | 2.239E-11 | 3.076 | 5.316E-10 |
| ENSG00000101336 | HCK | mRNA | 2.038 | 1.973E-13 | 2.456 | 6.088E-15 |
| ENSG00000101400 | SNTA1 | mRNA | -1.835 | 3.820E-15 | -1.119 | 7.129E-04 |
| ENSG00000101489 | CELF4 | mRNA | -2.610 | 3.852E-06 | -3.141 | 2.699E-06 |
| ENSG00000101670 | LIPG | mRNA | 1.520 | 6.615E-04 | 1.281 | 4.497E-03 |
| ENSG00000101746 | NOL4 | mRNA | -3.258 | 4.126E-05 | -4.019 | 3.796E-06 |
| ENSG00000101916 | TLR8 | mRNA | 4.307 | 2.214E-20 | 4.058 | 1.564E-08 |
| ENSG00000101935 | AMMECR1 | mRNA | 1.664 | 1.032E-12 | 1.137 | 3.256E-04 |
| ENSG00000101938 | CHRDL1 | mRNA | -1.273 | 5.199E-04 | -1.684 | 2.460E-04 |
| ENSG00000101955 | SRPX | mRNA | -1.092 | 2.813E-03 | -1.617 | 5.908E-07 |
| ENSG00000102001 | CACNA1F | mRNA | -1.422 | 1.579E-05 | -1.123 | 2.132E-03 |
| ENSG00000102007 | PLP2 | mRNA | 2.033 | 2.718E-13 | 1.665 | 5.157E-06 |
| ENSG00000102032 | RENBP | mRNA | 1.086 | 8.758E-04 | 1.581 | 5.146E-07 |
| ENSG00000102109 | PCSK1N | mRNA | -2.283 | 1.913E-05 | -2.405 | 1.155E-05 |
| ENSG00000102145 | GATA1 | mRNA | 3.319 | 6.455E-05 | 4.570 | 1.447E-11 |
| ENSG00000102271 | KLHL4 | mRNA | -1.507 | 3.917E-03 | -2.306 | 2.303E-04 |
| ENSG00000102287 | GABRE | mRNA | 1.157 | 5.200E-06 | 1.516 | 2.181E-06 |
| ENSG00000102313 | ITIH6 | mRNA | -1.766 | 1.876E-04 | -1.372 | 2.283E-03 |
| ENSG00000102385 | DRP2 | mRNA | -1.824 | 3.367E-05 | -1.400 | 4.609E-03 |
| ENSG00000102466 | FGF14 | mRNA | -2.180 | 1.194E-03 | -1.946 | 5.903E-03 |
| ENSG00000102468 | HTR2A | mRNA | -1.989 | 5.171E-03 | -2.749 | 5.583E-04 |
| ENSG00000102854 | MSLN | mRNA | -3.001 | 1.946E-07 | -2.752 | 1.970E-06 |
| ENSG00000102878 | HSF4 | mRNA | -2.444 | 9.526E-20 | -1.217 | 3.428E-04 |
| ENSG00000102962 | CCL22 | mRNA | 2.601 | 1.072E-08 | 2.180 | 8.677E-06 |
| ENSG00000103044 | HAS3 | mRNA | 2.238 | 1.982E-13 | 2.369 | 1.868E-11 |
| ENSG00000103187 | COTL1 | mRNA | 1.243 | 1.943E-07 | 1.548 | 1.405E-08 |
| ENSG00000103202 | NME4 | mRNA | -1.825 | 7.883E-17 | -1.358 | 2.448E-07 |
| ENSG00000103260 | METRN | mRNA | -2.577 | 2.956E-15 | -1.240 | 1.512E-03 |
| ENSG00000103310 | ZP2 | mRNA | -3.058 | 1.551E-04 | -3.288 | 4.187E-04 |
| ENSG00000103313 | MEFV | mRNA | 2.548 | 1.696E-10 | 1.945 | 1.563E-05 |
| ENSG00000103316 | CRYM | mRNA | -1.546 | 3.706E-07 | -1.765 | 8.787E-09 |
| ENSG00000103449 | SALL1 | mRNA | 2.638 | 1.028E-03 | 3.152 | 1.058E-07 |
| ENSG00000103569 | AQP9 | mRNA | 5.055 | 1.433E-18 | 4.281 | 3.431E-12 |
| ENSG00000103888 | KIAA1199 | mRNA | 1.751 | 1.231E-04 | 2.343 | 1.815E-06 |
| ENSG00000103942 | HOMER2 | mRNA | -1.909 | 5.731E-18 | -1.183 | 4.463E-06 |
| ENSG00000104043 | ATP8B4 | mRNA | 1.666 | 5.879E-10 | 1.464 | 7.681E-05 |
| ENSG00000104055 | TGM5 | mRNA | -2.312 | 1.003E-05 | -1.933 | 2.241E-04 |
| ENSG00000104059 | FAM189A1 | mRNA | -2.287 | 3.055E-06 | -1.718 | 5.151E-04 |
| ENSG00000104112 | SCG3 | mRNA | 4.819 | 9.087E-08 | 2.401 | 6.646E-03 |
| ENSG00000104267 | CA2 | mRNA | -3.640 | 1.049E-24 | -3.753 | 2.333E-16 |
| ENSG00000104312 | RIPK2 | mRNA | 1.386 | 2.915E-06 | 1.934 | 3.666E-06 |
| ENSG00000104327 | CALB1 | mRNA | 2.245 | 3.186E-07 | 1.231 | 1.257E-03 |
| ENSG00000104415 | WISP1 | mRNA | 2.221 | 8.881E-16 | 2.806 | 1.004E-16 |
| ENSG00000104490 | NCALD | mRNA | -2.168 | 1.548E-13 | -2.485 | 2.380E-14 |
| ENSG00000104783 | KCNN4 | mRNA | -4.668 | 5.912E-57 | -3.624 | 2.742E-18 |
| ENSG00000104888 | SLC17A7 | mRNA | -2.254 | 5.277E-07 | -1.206 | 7.664E-03 |
| ENSG00000104894 | CD37 | mRNA | 1.348 | 6.394E-07 | 1.311 | 4.033E-06 |
| ENSG00000104951 | IL4I1 | mRNA | 1.134 | 1.442E-03 | 1.297 | 1.394E-03 |
| ENSG00000104972 | LILRB1 | mRNA | 1.668 | 2.952E-08 | 1.849 | 3.959E-07 |
| ENSG00000104974 | LILRA1 | mRNA | 2.101 | 5.763E-09 | 1.748 | 1.817E-04 |
| ENSG00000105011 | ASF1B | mRNA | 1.581 | 1.876E-05 | 1.125 | 3.182E-03 |
| ENSG00000105048 | TNNT1 | mRNA | 2.127 | 1.800E-04 | 3.000 | 4.165E-07 |
| ENSG00000105205 | CLC | mRNA | 5.203 | 1.576E-09 | 6.876 | 3.774E-16 |
| ENSG00000105278 | ZFR2 | mRNA | -1.785 | 5.474E-03 | -3.674 | 2.933E-06 |
| ENSG00000105289 | TJP3 | mRNA | -1.197 | 1.499E-04 | -1.256 | 2.757E-04 |
| ENSG00000105352 | CEACAM4 | mRNA | 1.707 | 5.643E-05 | 1.626 | 2.679E-04 |
| ENSG00000105366 | SIGLEC8 | mRNA | 2.987 | 1.924E-07 | 4.257 | 4.547E-21 |
| ENSG00000105374 | NKG7 | mRNA | 1.200 | 4.990E-04 | 1.159 | 4.539E-04 |
| ENSG00000105383 | CD33 | mRNA | 2.233 | 1.600E-11 | 2.245 | 9.421E-10 |
| ENSG00000105388 | CEACAM5 | mRNA | 3.798 | 1.238E-13 | 3.284 | 9.506E-13 |
| ENSG00000105492 | SIGLEC6 | mRNA | 1.385 | 2.424E-04 | 1.959 | 4.261E-07 |
| ENSG00000105501 | SIGLEC5 | mRNA | 3.640 | 2.681E-10 | 2.966 | 1.017E-04 |
| ENSG00000105519 | CAPS | mRNA | -2.125 | 9.159E-05 | -1.790 | 4.518E-04 |
| ENSG00000105609 | LILRB5 | mRNA | 1.301 | 1.055E-03 | 2.739 | 6.928E-10 |
| ENSG00000105639 | JAK3 | mRNA | 1.138 | 2.989E-05 | 1.123 | 8.351E-05 |
| ENSG00000105810 | CDK6 | mRNA | 1.419 | 8.349E-12 | 1.127 | 1.334E-04 |
| ENSG00000105928 | DFNA5 | mRNA | 1.107 | 6.466E-06 | 1.183 | 2.086E-05 |
| ENSG00000105967 | TFEC | mRNA | 2.934 | 7.658E-08 | 2.103 | 2.908E-03 |
| ENSG00000105976 | MET | mRNA | 1.634 | 2.968E-16 | 1.235 | 2.456E-05 |
| ENSG00000105989 | WNT2 | mRNA | 4.299 | 1.074E-04 | 4.141 | 1.685E-07 |
| ENSG00000105991 | HOXA1 | mRNA | 2.636 | 7.335E-08 | 2.820 | 2.983E-06 |
| ENSG00000106066 | CPVL | mRNA | 2.301 | 1.049E-21 | 1.809 | 2.567E-10 |
| ENSG00000106078 | COBL | mRNA | -1.703 | 9.613E-13 | -1.469 | 1.108E-09 |
| ENSG00000106178 | CCL24 | mRNA | 2.765 | 7.959E-05 | 4.401 | 8.621E-10 |
| ENSG00000106351 | AGFG2 | mRNA | -2.392 | 3.121E-32 | -1.905 | 1.200E-12 |
| ENSG00000106366 | SERPINE1 | mRNA | 1.941 | 1.204E-04 | 2.902 | 1.008E-08 |
| ENSG00000106483 | SFRP4 | mRNA | -1.697 | 6.579E-04 | -1.501 | 6.381E-03 |
| ENSG00000106565 | TMEM176B | mRNA | 1.266 | 5.237E-09 | 1.581 | 3.269E-12 |
| ENSG00000106809 | OGN | mRNA | -1.605 | 1.701E-02 | -3.228 | 1.470E-04 |
| ENSG00000106952 | TNFSF8 | mRNA | 2.090 | 6.085E-08 | 1.676 | 3.380E-04 |
| ENSG00000107159 | CA9 | mRNA | 3.667 | 8.283E-09 | 5.074 | 3.427E-12 |
| ENSG00000107165 | TYRP1 | mRNA | 2.849 | 7.903E-05 | 2.587 | 1.936E-03 |
| ENSG00000107201 | DDX58 | mRNA | 1.036 | 6.590E-07 | 1.021 | 4.247E-03 |
| ENSG00000107281 | NPDC1 | mRNA | -2.790 | 3.404E-23 | -1.324 | 3.015E-04 |
| ENSG00000107623 | GDF10 | mRNA | -2.755 | 1.245E-08 | -3.087 | 3.400E-05 |
| ENSG00000107798 | LIPA | mRNA | 1.504 | 5.951E-10 | 1.457 | 2.955E-05 |
| ENSG00000107821 | KAZALD1 | mRNA | -2.618 | 2.976E-20 | -1.921 | 1.885E-09 |
| ENSG00000108679 | LGALS3BP | mRNA | -1.430 | 3.243E-09 | -1.223 | 1.981E-05 |
| ENSG00000108691 | CCL2 | mRNA | 3.099 | 1.683E-09 | 2.158 | 1.275E-03 |
| ENSG00000108700 | CCL8 | mRNA | 3.961 | 2.438E-12 | 3.772 | 6.816E-07 |
| ENSG00000108823 | SGCA | mRNA | -1.321 | 3.218E-03 | -1.072 | 6.108E-03 |
| ENSG00000108932 | SLC16A6 | mRNA | 1.981 | 4.535E-09 | 1.478 | 1.771E-03 |
| ENSG00000108960 | MMD | mRNA | 1.253 | 7.776E-07 | 1.483 | 9.739E-07 |
| ENSG00000109084 | TMEM97 | mRNA | 1.059 | 4.003E-05 | 1.377 | 8.661E-05 |
| ENSG00000109205 | ODAM | mRNA | -4.703 | 4.409E-11 | -2.847 | 4.588E-04 |
| ENSG00000109208 | SMR3A | mRNA | -6.217 | 1.026E-35 | -3.786 | 3.687E-07 |
| ENSG00000109255 | NMU | mRNA | 3.405 | 4.197E-07 | 3.075 | 1.774E-04 |
| ENSG00000109674 | NEIL3 | mRNA | 3.151 | 1.239E-09 | 2.233 | 7.361E-04 |
| ENSG00000109684 | CLNK | mRNA | 2.071 | 3.966E-09 | 1.832 | 2.873E-06 |
| ENSG00000109738 | GLRB | mRNA | -2.265 | 3.382E-07 | -3.654 | 3.041E-14 |
| ENSG00000109805 | NCAPG | mRNA | 3.460 | 3.154E-11 | 2.819 | 4.549E-06 |
| ENSG00000109846 | CRYAB | mRNA | -1.555 | 4.502E-08 | -1.326 | 3.730E-05 |
| ENSG00000109861 | CTSC | mRNA | 1.881 | 6.844E-09 | 2.152 | 4.640E-09 |
| ENSG00000109956 | B3GAT1 | mRNA | -1.985 | 2.904E-05 | -1.439 | 9.963E-04 |
| ENSG00000110042 | DTX4 | mRNA | -1.149 | 2.956E-08 | -1.090 | 2.111E-05 |
| ENSG00000110077 | MS4A6A | mRNA | 2.577 | 1.349E-14 | 2.482 | 7.190E-10 |
| ENSG00000110079 | MS4A4A | mRNA | 3.234 | 9.095E-16 | 3.808 | 1.545E-19 |
| ENSG00000110080 | ST3GAL4 | mRNA | -1.664 | 3.076E-14 | -1.116 | 1.881E-05 |
| ENSG00000110195 | FOLR1 | mRNA | -3.970 | 1.436E-19 | -3.912 | 1.603E-16 |
| ENSG00000110203 | FOLR3 | mRNA | 2.904 | 1.489E-04 | 4.583 | 8.461E-06 |
| ENSG00000110324 | IL10RA | mRNA | 1.217 | 2.550E-06 | 1.484 | 3.981E-10 |
| ENSG00000110347 | MMP12 | mRNA | 5.483 | 1.148E-08 | 3.401 | 6.426E-04 |
| ENSG00000110395 | CBL | mRNA | 1.187 | 7.990E-10 | 1.115 | 6.275E-06 |
| ENSG00000110436 | SLC1A2 | mRNA | -1.218 | 8.907E-07 | -1.158 | 6.123E-05 |
| ENSG00000110484 | SCGB2A2 | mRNA | -9.455 | 1.830E-07 | -9.384 | 5.394E-07 |
| ENSG00000110721 | CHKA | mRNA | -1.898 | 8.805E-18 | -1.754 | 4.353E-12 |
| ENSG00000110786 | PTPN5 | mRNA | -2.089 | 9.153E-05 | -1.304 | 2.807E-03 |
| ENSG00000110848 | CD69 | mRNA | 2.642 | 5.427E-08 | 3.205 | 7.738E-08 |
| ENSG00000110852 | CLEC2B | mRNA | 2.510 | 1.087E-14 | 1.566 | 6.233E-04 |
| ENSG00000110934 | BIN2 | mRNA | 1.938 | 4.704E-11 | 2.016 | 2.684E-09 |
| ENSG00000111052 | LIN7A | mRNA | 1.833 | 1.702E-05 | 1.927 | 3.884E-04 |
| ENSG00000111181 | SLC6A12 | mRNA | 1.985 | 1.184E-03 | 2.205 | 8.828E-04 |
| ENSG00000111206 | FOXM1 | mRNA | 2.116 | 3.879E-07 | 1.844 | 2.574E-05 |
| ENSG00000111261 | MANSC1 | mRNA | -2.439 | 7.610E-17 | -2.734 | 1.606E-15 |
| ENSG00000111262 | KCNA1 | mRNA | -2.962 | 1.474E-05 | -3.100 | 3.749E-05 |
| ENSG00000111291 | GPRC5D | mRNA | -2.234 | 1.803E-05 | -2.231 | 6.237E-05 |
| ENSG00000111341 | MGP | mRNA | 1.531 | 4.145E-06 | 1.408 | 5.632E-04 |
| ENSG00000111344 | RASAL1 | mRNA | -4.664 | 2.656E-47 | -3.363 | 8.189E-15 |
| ENSG00000111348 | ARHGDIB | mRNA | 1.619 | 1.712E-09 | 1.452 | 1.045E-05 |
| ENSG00000111490 | TBC1D30 | mRNA | -1.967 | 9.071E-18 | -2.097 | 2.540E-16 |
| ENSG00000111665 | CDCA3 | mRNA | 1.823 | 4.834E-06 | 1.782 | 1.234E-05 |
| ENSG00000111666 | CHPT1 | mRNA | -1.591 | 1.461E-11 | -1.504 | 2.524E-08 |
| ENSG00000111728 | ST8SIA1 | mRNA | 1.313 | 9.091E-09 | 1.266 | 1.240E-05 |
| ENSG00000111729 | CLEC4A | mRNA | 1.896 | 2.353E-09 | 1.702 | 2.913E-06 |
| ENSG00000111799 | COL12A1 | mRNA | 1.673 | 1.525E-11 | 1.935 | 1.313E-11 |
| ENSG00000111817 | DSE | mRNA | 1.727 | 1.756E-18 | 1.831 | 1.985E-12 |
| ENSG00000111886 | GABRR2 | mRNA | 1.530 | 1.092E-05 | 1.216 | 4.549E-03 |
| ENSG00000111907 | TPD52L1 | mRNA | -1.135 | 9.024E-05 | -1.525 | 6.352E-06 |
| ENSG00000111913 | FAM65B | mRNA | 1.424 | 6.231E-11 | 1.468 | 3.518E-07 |
| ENSG00000112077 | RHAG | mRNA | -4.137 | 5.654E-04 | -3.139 | 7.794E-03 |
| ENSG00000112137 | PHACTR1 | mRNA | 1.124 | 3.503E-05 | 1.003 | 4.348E-03 |
| ENSG00000112195 | TREML2 | mRNA | 2.218 | 8.755E-06 | 3.528 | 3.342E-12 |
| ENSG00000112242 | E2F3 | mRNA | 1.567 | 3.816E-13 | 1.342 | 8.302E-08 |
| ENSG00000112276 | BVES | mRNA | -1.387 | 1.395E-06 | -1.232 | 1.522E-04 |
| ENSG00000112280 | COL9A1 | mRNA | -3.219 | 1.696E-10 | -2.605 | 1.431E-06 |
| ENSG00000112290 | WASF1 | mRNA | 1.483 | 1.125E-09 | 1.357 | 1.315E-06 |
| ENSG00000112303 | VNN2 | mRNA | 2.959 | 1.178E-15 | 1.633 | 4.757E-04 |
| ENSG00000112379 | KIAA1244 | mRNA | -1.587 | 3.264E-18 | -1.646 | 6.128E-14 |
| ENSG00000112414 | GPR126 | mRNA | -2.568 | 4.128E-13 | -2.306 | 4.053E-08 |
| ENSG00000112742 | TTK | mRNA | 3.140 | 3.613E-12 | 2.125 | 8.469E-04 |
| ENSG00000112773 | FAM46A | mRNA | -1.498 | 6.027E-09 | -1.575 | 4.372E-07 |
| ENSG00000112799 | LY86 | mRNA | 2.468 | 4.149E-08 | 1.497 | 1.132E-03 |
| ENSG00000112812 | PRSS16 | mRNA | -1.150 | 5.351E-04 | -1.296 | 2.900E-04 |
| ENSG00000112984 | KIF20A | mRNA | 3.385 | 1.735E-12 | 2.734 | 3.502E-06 |
| ENSG00000113083 | LOX | mRNA | 1.041 | 5.214E-07 | 1.161 | 8.170E-06 |
| ENSG00000113088 | GZMK | mRNA | 3.015 | 4.864E-12 | 2.213 | 2.451E-06 |
| ENSG00000113209 | PCDHB5 | mRNA | -1.465 | 1.226E-06 | -1.161 | 3.633E-04 |
| ENSG00000113249 | HAVCR1 | mRNA | 1.713 | 5.501E-04 | 1.312 | 8.397E-03 |
| ENSG00000113263 | ITK | mRNA | 1.560 | 1.067E-06 | 1.101 | 3.010E-04 |
| ENSG00000113303 | BTNL8 | mRNA | 1.879 | 7.088E-05 | 2.275 | 7.556E-04 |
| ENSG00000113368 | LMNB1 | mRNA | 1.822 | 3.746E-12 | 1.432 | 9.733E-07 |
| ENSG00000113532 | ST8SIA4 | mRNA | 1.948 | 4.271E-14 | 1.402 | 5.149E-05 |
| ENSG00000113645 | WWC1 | mRNA | -2.322 | 3.678E-28 | -1.895 | 1.323E-13 |
| ENSG00000113749 | HRH2 | mRNA | 1.367 | 4.714E-06 | 1.677 | 4.134E-08 |
| ENSG00000113763 | UNC5A | mRNA | -3.939 | 1.266E-07 | -2.612 | 1.830E-05 |
| ENSG00000113810 | SMC4 | mRNA | 1.879 | 5.878E-18 | 1.288 | 7.257E-06 |
| ENSG00000114013 | CD86 | mRNA | 2.634 | 1.917E-15 | 2.482 | 5.457E-11 |
| ENSG00000114248 | LRRC31 | mRNA | -4.039 | 2.618E-07 | -1.963 | 3.831E-03 |
| ENSG00000114251 | WNT5A | mRNA | 1.096 | 2.318E-04 | 1.170 | 4.204E-04 |
| ENSG00000114279 | FGF12 | mRNA | -2.387 | 4.320E-15 | -2.054 | 7.965E-07 |
| ENSG00000114487 | MORC1 | mRNA | 4.372 | 2.110E-09 | 2.635 | 3.276E-04 |
| ENSG00000114547 | ROPN1B | mRNA | -1.375 | 8.226E-04 | -1.329 | 6.263E-04 |
| ENSG00000114631 | PODXL2 | mRNA | -2.652 | 2.326E-29 | -2.312 | 2.060E-15 |
| ENSG00000114638 | UPK1B | mRNA | 2.686 | 4.825E-06 | 1.847 | 2.576E-04 |
| ENSG00000114646 | CSPG5 | mRNA | -1.369 | 1.739E-03 | -1.247 | 7.231E-03 |
| ENSG00000114654 | EFCC1 | mRNA | -1.571 | 3.513E-06 | -1.127 | 2.497E-03 |
| ENSG00000114771 | AADAC | mRNA | 2.240 | 8.709E-05 | 2.526 | 2.367E-05 |
| ENSG00000114812 | VIPR1 | mRNA | -2.100 | 7.986E-25 | -1.412 | 6.846E-08 |
| ENSG00000114948 | ADAM23 | mRNA | -1.359 | 1.584E-03 | -1.453 | 1.731E-03 |
| ENSG00000114978 | MOB1A | mRNA | 1.711 | 7.678E-10 | 1.132 | 4.021E-03 |
| ENSG00000115008 | IL1A | mRNA | 3.942 | 8.520E-08 | 4.878 | 6.331E-08 |
| ENSG00000115042 | FAHD2A | mRNA | -1.920 | 2.924E-17 | -1.670 | 6.131E-10 |
| ENSG00000115091 | ACTR3 | mRNA | 1.660 | 1.998E-09 | 1.149 | 3.425E-03 |
| ENSG00000115163 | CENPA | mRNA | 3.202 | 6.583E-09 | 2.740 | 1.113E-05 |
| ENSG00000115165 | CYTIP | mRNA | 1.175 | 1.703E-05 | 1.514 | 1.058E-05 |
| ENSG00000115221 | ITGB6 | mRNA | 1.692 | 5.906E-14 | 1.301 | 3.094E-07 |
| ENSG00000115232 | ITGA4 | mRNA | 1.996 | 1.505E-08 | 1.245 | 2.226E-03 |
| ENSG00000115255 | REEP6 | mRNA | -1.367 | 1.447E-07 | -1.184 | 3.384E-05 |
| ENSG00000115266 | APC2 | mRNA | -3.537 | 2.631E-20 | -2.415 | 9.619E-08 |
| ENSG00000115295 | CLIP4 | mRNA | 1.176 | 6.365E-06 | 1.088 | 9.440E-04 |
| ENSG00000115353 | TACR1 | mRNA | 1.324 | 5.794E-06 | 2.028 | 7.489E-07 |
| ENSG00000115361 | ACADL | mRNA | -3.287 | 2.582E-13 | -3.283 | 2.575E-12 |
| ENSG00000115380 | EFEMP1 | mRNA | 1.633 | 1.676E-10 | 1.363 | 7.820E-06 |
| ENSG00000115523 | GNLY | mRNA | 1.543 | 1.554E-03 | 1.195 | 4.157E-03 |
| ENSG00000115602 | IL1RL1 | mRNA | 2.703 | 1.101E-08 | 3.874 | 2.143E-14 |
| ENSG00000115616 | SLC9A2 | mRNA | -1.252 | 1.365E-04 | -1.595 | 9.797E-08 |
| ENSG00000115648 | MLPH | mRNA | -1.554 | 1.997E-13 | -1.089 | 7.535E-05 |
| ENSG00000115935 | WIPF1 | mRNA | 1.705 | 4.229E-11 | 1.564 | 4.054E-08 |
| ENSG00000115956 | PLEK | mRNA | 2.385 | 6.296E-19 | 2.721 | 4.244E-15 |
| ENSG00000116032 | GRIN3B | mRNA | -2.149 | 1.163E-03 | -1.827 | 5.409E-03 |
| ENSG00000116096 | SPR | mRNA | -2.107 | 1.355E-15 | -1.443 | 3.486E-07 |
| ENSG00000116194 | ANGPTL1 | mRNA | -1.199 | 2.360E-02 | -2.884 | 3.613E-07 |
| ENSG00000116260 | QSOX1 | mRNA | -1.891 | 1.932E-18 | -1.223 | 2.602E-05 |
| ENSG00000116299 | KIAA1324 | mRNA | -3.205 | 1.729E-31 | -2.801 | 3.181E-27 |
| ENSG00000116489 | CAPZA1 | mRNA | 1.712 | 6.542E-09 | 1.166 | 3.909E-03 |
| ENSG00000116701 | NCF2 | mRNA | 2.525 | 1.324E-15 | 3.222 | 3.504E-20 |
| ENSG00000116741 | RGS2 | mRNA | 2.358 | 4.812E-09 | 2.356 | 3.222E-08 |
| ENSG00000116745 | RPE65 | mRNA | 2.912 | 3.268E-11 | 2.929 | 4.950E-06 |
| ENSG00000116771 | AGMAT | mRNA | -1.711 | 3.927E-09 | -1.914 | 1.235E-08 |
| ENSG00000116815 | CD58 | mRNA | 1.344 | 6.323E-09 | 1.133 | 2.214E-05 |
| ENSG00000117009 | KMO | mRNA | 1.996 | 6.766E-06 | 1.739 | 7.719E-04 |
| ENSG00000117069 | ST6GALNAC5 | mRNA | 1.381 | 1.541E-02 | 1.926 | 1.943E-03 |
| ENSG00000117090 | SLAMF1 | mRNA | 1.627 | 5.619E-06 | 1.585 | 9.504E-05 |
| ENSG00000117091 | CD48 | mRNA | 1.813 | 1.189E-09 | 1.239 | 2.646E-03 |
| ENSG00000117115 | PADI2 | mRNA | -2.884 | 1.213E-22 | -2.496 | 2.891E-14 |
| ENSG00000117152 | RGS4 | mRNA | 2.102 | 4.572E-08 | 2.752 | 1.745E-10 |
| ENSG00000117228 | GBP1 | mRNA | 1.733 | 9.667E-05 | 1.826 | 7.365E-03 |
| ENSG00000117394 | SLC2A1 | mRNA | 1.044 | 3.161E-04 | 1.587 | 2.940E-06 |
| ENSG00000117399 | CDC20 | mRNA | 1.999 | 2.103E-08 | 1.654 | 2.478E-04 |
| ENSG00000117425 | PTCH2 | mRNA | -1.781 | 1.083E-14 | -1.679 | 4.483E-11 |
| ENSG00000117507 | FMO6P | mRNA | 1.230 | 2.851E-04 | 1.222 | 3.085E-03 |
| ENSG00000117560 | FASLG | mRNA | 2.062 | 9.123E-08 | 1.177 | 2.484E-03 |
| ENSG00000117594 | HSD11B1 | mRNA | 1.711 | 1.122E-02 | 2.692 | 9.616E-04 |
| ENSG00000117724 | CENPF | mRNA | 2.473 | 1.067E-15 | 1.858 | 1.438E-05 |
| ENSG00000117983 | MUC5B | mRNA | -3.809 | 4.982E-11 | -4.408 | 7.341E-16 |
| ENSG00000118156 | ZNF541 | mRNA | -1.774 | 7.384E-07 | -1.326 | 6.860E-04 |
| ENSG00000118193 | KIF14 | mRNA | 3.480 | 5.912E-16 | 2.875 | 2.904E-07 |
| ENSG00000118308 | LRMP | mRNA | 2.435 | 1.588E-09 | 2.482 | 3.742E-07 |
| ENSG00000118473 | SGIP1 | mRNA | 2.136 | 9.530E-06 | 2.641 | 2.552E-07 |
| ENSG00000118596 | SLC16A7 | mRNA | 1.790 | 3.113E-12 | 1.169 | 8.209E-04 |
| ENSG00000119121 | TRPM6 | mRNA | 1.826 | 2.526E-08 | 2.012 | 8.433E-12 |
| ENSG00000119125 | GDA | mRNA | 3.112 | 2.518E-08 | 1.792 | 5.948E-03 |
| ENSG00000119283 | TRIM67 | mRNA | 2.589 | 1.246E-07 | 3.820 | 1.209E-08 |
| ENSG00000119411 | BSPRY | mRNA | -1.585 | 1.388E-13 | -1.535 | 3.268E-09 |
| ENSG00000119514 | GALNT12 | mRNA | -1.222 | 2.691E-05 | -1.321 | 8.156E-06 |
| ENSG00000119535 | CSF3R | mRNA | 2.652 | 5.260E-14 | 2.436 | 6.676E-12 |
| ENSG00000119915 | ELOVL3 | mRNA | 1.694 | 1.042E-02 | 1.914 | 5.300E-03 |
| ENSG00000119917 | IFIT3 | mRNA | 1.581 | 1.930E-05 | 2.000 | 4.532E-03 |
| ENSG00000119922 | IFIT2 | mRNA | 1.678 | 4.520E-06 | 2.329 | 1.161E-03 |
| ENSG00000120055 | C10orf95 | mRNA | -1.696 | 5.377E-03 | -1.610 | 6.434E-03 |
| ENSG00000120057 | SFRP5 | mRNA | -4.700 | 4.011E-08 | -3.027 | 6.956E-05 |
| ENSG00000120217 | CD274 | mRNA | 1.935 | 1.544E-07 | 2.293 | 2.495E-05 |
| ENSG00000120251 | GRIA2 | mRNA | -7.377 | 8.916E-22 | -4.890 | 1.549E-12 |
| ENSG00000120254 | MTHFD1L | mRNA | 1.293 | 1.543E-10 | 1.155 | 1.031E-07 |
| ENSG00000120280 | CXorf21 | mRNA | 1.508 | 1.774E-06 | 1.554 | 1.208E-04 |
| ENSG00000120332 | TNN | mRNA | -1.538 | 7.377E-03 | -1.889 | 5.777E-03 |
| ENSG00000120337 | TNFSF18 | mRNA | 2.096 | 1.879E-05 | 2.656 | 2.800E-04 |
| ENSG00000120341 | SEC16B | mRNA | -2.356 | 1.773E-12 | -1.300 | 1.628E-04 |
| ENSG00000120440 | TTLL2 | mRNA | -2.683 | 1.248E-05 | -2.200 | 1.540E-04 |
| ENSG00000120498 | TEX11 | mRNA | 2.021 | 1.029E-03 | 1.990 | 2.073E-03 |
| ENSG00000120586 | MRC1 | mRNA | 2.344 | 9.772E-05 | 3.322 | 2.055E-07 |
| ENSG00000120594 | PLXDC2 | mRNA | 1.184 | 2.319E-08 | 1.010 | 3.260E-05 |
| ENSG00000120693 | SMAD9 | mRNA | -1.685 | 1.851E-11 | -1.730 | 1.279E-09 |
| ENSG00000120756 | PLS1 | mRNA | 2.022 | 2.383E-15 | 1.413 | 7.759E-05 |
| ENSG00000120875 | DUSP4 | mRNA | -1.804 | 3.234E-11 | -1.806 | 9.092E-08 |
| ENSG00000120885 | CLU | mRNA | -1.374 | 7.548E-09 | -1.207 | 3.154E-05 |
| ENSG00000120907 | ADRA1A | mRNA | -2.516 | 8.074E-08 | -1.919 | 1.874E-03 |
| ENSG00000120915 | EPHX2 | mRNA | -1.221 | 3.857E-12 | -1.095 | 4.404E-07 |
| ENSG00000120949 | TNFRSF8 | mRNA | 1.390 | 5.774E-05 | 1.112 | 3.494E-03 |
| ENSG00000121075 | TBX4 | mRNA | 1.702 | 9.105E-04 | 2.995 | 6.702E-12 |
| ENSG00000121152 | NCAPH | mRNA | 2.182 | 1.223E-08 | 1.429 | 2.413E-04 |
| ENSG00000121207 | LRAT | mRNA | -2.558 | 3.309E-09 | -2.486 | 1.917E-07 |
| ENSG00000121316 | PLBD1 | mRNA | 1.513 | 2.043E-09 | 1.073 | 1.004E-03 |
| ENSG00000121413 | ZSCAN18 | mRNA | -2.147 | 3.701E-10 | -1.037 | 8.021E-03 |
| ENSG00000121594 | CD80 | mRNA | 2.616 | 5.590E-07 | 1.762 | 1.234E-03 |
| ENSG00000121621 | KIF18A | mRNA | 3.105 | 3.561E-14 | 2.211 | 3.763E-05 |
| ENSG00000121671 | CRY2 | mRNA | -1.489 | 8.619E-14 | -1.034 | 1.792E-05 |
| ENSG00000121742 | GJB6 | mRNA | 2.959 | 8.425E-13 | 3.048 | 1.820E-12 |
| ENSG00000121769 | FABP3 | mRNA | 1.496 | 1.293E-03 | 2.269 | 2.905E-05 |
| ENSG00000121797 | CCRL2 | mRNA | 1.414 | 1.588E-05 | 1.244 | 7.728E-04 |
| ENSG00000121807 | CCR2 | mRNA | 1.467 | 2.381E-06 | 1.947 | 5.274E-06 |
| ENSG00000121895 | TMEM156 | mRNA | 1.591 | 8.754E-06 | 1.625 | 2.537E-04 |
| ENSG00000121933 | ADORA3 | mRNA | 2.595 | 1.577E-07 | 4.226 | 2.225E-21 |
| ENSG00000121957 | GPSM2 | mRNA | 1.618 | 1.980E-13 | 1.164 | 1.053E-04 |
| ENSG00000122025 | FLT3 | mRNA | 1.366 | 2.754E-04 | 1.193 | 6.709E-03 |
| ENSG00000122122 | SASH3 | mRNA | 1.183 | 9.194E-05 | 1.388 | 8.007E-06 |
| ENSG00000122188 | LAX1 | mRNA | 1.589 | 1.400E-07 | 1.675 | 4.891E-06 |
| ENSG00000122223 | CD244 | mRNA | 1.744 | 4.815E-07 | 1.840 | 1.673E-05 |
| ENSG00000122574 | WIPF3 | mRNA | -1.340 | 1.428E-05 | -1.362 | 6.654E-05 |
| ENSG00000122591 | FAM126A | mRNA | 1.632 | 9.444E-07 | 1.261 | 2.928E-03 |
| ENSG00000122641 | INHBA | mRNA | 1.858 | 3.838E-08 | 2.470 | 9.275E-12 |
| ENSG00000122756 | CNTFR | mRNA | -4.053 | 3.934E-18 | -3.172 | 6.604E-08 |
| ENSG00000122824 | NUDT10 | mRNA | 1.569 | 1.599E-05 | 1.468 | 9.154E-06 |
| ENSG00000122861 | PLAU | mRNA | 1.869 | 8.511E-12 | 1.901 | 8.028E-12 |
| ENSG00000122862 | SRGN | mRNA | 2.136 | 2.643E-11 | 2.710 | 6.572E-11 |
| ENSG00000122986 | HVCN1 | mRNA | 1.099 | 9.386E-05 | 1.137 | 2.529E-04 |
| ENSG00000123119 | NECAB1 | mRNA | -2.556 | 7.022E-05 | -2.048 | 3.631E-03 |
| ENSG00000123219 | CENPK | mRNA | 2.563 | 3.584E-15 | 1.488 | 7.321E-04 |
| ENSG00000123329 | ARHGAP9 | mRNA | 1.141 | 1.478E-05 | 1.686 | 6.140E-08 |
| ENSG00000123338 | NCKAP1L | mRNA | 1.613 | 6.058E-11 | 1.667 | 2.122E-10 |
| ENSG00000123405 | NFE2 | mRNA | 2.659 | 3.590E-07 | 3.602 | 2.271E-11 |
| ENSG00000123427 | METTL21B | mRNA | -2.839 | 2.225E-09 | -1.584 | 3.960E-07 |
| ENSG00000123485 | HJURP | mRNA | 1.933 | 1.327E-05 | 1.553 | 6.546E-04 |
| ENSG00000123500 | COL10A1 | mRNA | 1.970 | 4.217E-12 | 1.692 | 5.283E-08 |
| ENSG00000123560 | PLP1 | mRNA | -2.825 | 1.009E-08 | -3.267 | 4.098E-11 |
| ENSG00000123610 | TNFAIP6 | mRNA | 2.677 | 3.635E-07 | 1.675 | 3.038E-03 |
| ENSG00000123612 | ACVR1C | mRNA | -1.731 | 2.712E-05 | -2.174 | 3.270E-06 |
| ENSG00000123689 | G0S2 | mRNA | 1.701 | 9.208E-04 | 2.836 | 1.785E-06 |
| ENSG00000123700 | KCNJ2 | mRNA | 1.469 | 5.262E-13 | 1.458 | 2.852E-06 |
| ENSG00000123975 | CKS2 | mRNA | 3.671 | 2.172E-17 | 2.981 | 3.896E-05 |
| ENSG00000124003 | MOGAT1 | mRNA | -3.832 | 1.933E-04 | -3.726 | 3.815E-04 |
| ENSG00000124107 | SLPI | mRNA | -3.772 | 4.144E-45 | -2.918 | 1.938E-17 |
| ENSG00000124134 | KCNS1 | mRNA | -1.989 | 1.515E-10 | -2.225 | 1.334E-08 |
| ENSG00000124159 | MATN4 | mRNA | -5.083 | 1.062E-29 | -3.922 | 5.205E-12 |
| ENSG00000124191 | TOX2 | mRNA | 1.123 | 1.765E-04 | 1.469 | 2.842E-06 |
| ENSG00000124205 | EDN3 | mRNA | -3.088 | 6.717E-10 | -1.622 | 4.111E-03 |
| ENSG00000124215 | CDH26 | mRNA | 2.237 | 1.516E-06 | 3.087 | 1.486E-15 |
| ENSG00000124249 | KCNK15 | mRNA | -2.163 | 3.185E-05 | -2.188 | 1.867E-05 |
| ENSG00000124334 | IL9R | mRNA | 1.542 | 4.055E-03 | 2.265 | 1.246E-05 |
| ENSG00000124343 | XG | mRNA | 1.819 | 1.828E-06 | 1.665 | 5.187E-05 |
| ENSG00000124374 | PAIP2B | mRNA | -1.959 | 2.867E-11 | -2.069 | 2.723E-10 |
| ENSG00000124466 | LYPD3 | mRNA | 1.420 | 3.453E-03 | 1.820 | 9.022E-04 |
| ENSG00000124490 | CRISP2 | mRNA | -2.886 | 4.952E-13 | -2.755 | 3.615E-11 |
| ENSG00000124491 | F13A1 | mRNA | 2.539 | 1.407E-12 | 3.615 | 2.451E-21 |
| ENSG00000124635 | HIST1H2BJ | mRNA | 3.211 | 1.625E-14 | 2.199 | 7.418E-06 |
| ENSG00000124664 | SPDEF | mRNA | -2.253 | 4.658E-12 | -1.248 | 3.932E-05 |
| ENSG00000124693 | HIST1H3B | mRNA | 2.716 | 9.971E-12 | 2.428 | 1.804E-08 |
| ENSG00000124713 | GNMT | mRNA | -2.264 | 6.065E-11 | -2.464 | 1.860E-10 |
| ENSG00000124731 | TREM1 | mRNA | 3.082 | 1.370E-11 | 3.075 | 1.677E-10 |
| ENSG00000124839 | RAB17 | mRNA | -1.894 | 4.013E-11 | -1.595 | 8.627E-06 |
| ENSG00000124882 | EREG | mRNA | 4.754 | 8.370E-09 | 5.549 | 5.266E-11 |
| ENSG00000124935 | SCGB1D2 | mRNA | -6.738 | 2.715E-08 | -5.109 | 2.801E-05 |
| ENSG00000125398 | SOX9 | mRNA | -1.965 | 2.743E-19 | -1.837 | 2.445E-12 |
| ENSG00000125508 | SRMS | mRNA | -1.775 | 1.320E-09 | -1.315 | 4.691E-04 |
| ENSG00000125538 | IL1B | mRNA | 3.385 | 2.027E-10 | 2.376 | 5.377E-05 |
| ENSG00000125675 | GRIA3 | mRNA | 1.975 | 2.890E-04 | 2.037 | 2.298E-04 |
| ENSG00000125726 | CD70 | mRNA | 2.057 | 3.224E-04 | 1.947 | 1.648E-03 |
| ENSG00000125851 | PCSK2 | mRNA | -2.231 | 1.818E-04 | -3.691 | 5.066E-12 |
| ENSG00000125869 | LAMP5 | mRNA | 1.903 | 8.974E-11 | 2.434 | 3.903E-09 |
| ENSG00000125900 | SIRPD | mRNA | 3.340 | 1.739E-06 | 3.161 | 8.929E-06 |
| ENSG00000125965 | GDF5 | mRNA | -4.663 | 1.209E-15 | -3.494 | 4.599E-08 |
| ENSG00000125968 | ID1 | mRNA | -1.966 | 4.510E-14 | -1.492 | 1.164E-05 |
| ENSG00000125999 | BPIFB1 | mRNA | -3.557 | 4.543E-22 | -2.553 | 1.884E-16 |
| ENSG00000126262 | FFAR2 | mRNA | 3.269 | 8.551E-11 | 4.385 | 1.253E-13 |
| ENSG00000126264 | HCST | mRNA | 1.126 | 3.073E-04 | 1.154 | 8.122E-04 |
| ENSG00000126500 | FLRT1 | mRNA | -3.536 | 6.463E-20 | -2.674 | 3.567E-11 |
| ENSG00000126549 | STATH | mRNA | -7.998 | 8.339E-21 | -5.813 | 1.013E-07 |
| ENSG00000126733 | DACH2 | mRNA | -4.228 | 1.119E-09 | -3.566 | 2.899E-05 |
| ENSG00000126787 | DLGAP5 | mRNA | 3.567 | 3.700E-13 | 3.010 | 4.108E-07 |
| ENSG00000126860 | EVI2A | mRNA | 3.085 | 3.094E-08 | 2.513 | 5.808E-04 |
| ENSG00000126878 | AIF1L | mRNA | -1.995 | 7.457E-16 | -1.797 | 3.860E-12 |
| ENSG00000126950 | TMEM35 | mRNA | -1.151 | 6.299E-03 | -1.496 | 1.675E-04 |
| ENSG00000127074 | RGS13 | mRNA | 3.046 | 5.107E-10 | 2.975 | 3.141E-05 |
| ENSG00000127325 | BEST3 | mRNA | -1.754 | 8.137E-04 | -1.564 | 4.430E-03 |
| ENSG00000127418 | FGFRL1 | mRNA | -2.322 | 3.239E-28 | -1.452 | 1.902E-06 |
| ENSG00000127507 | EMR2 | mRNA | 2.603 | 7.312E-17 | 2.281 | 1.903E-12 |
| ENSG00000127564 | PKMYT1 | mRNA | 1.265 | 1.256E-02 | 1.495 | 5.513E-03 |
| ENSG00000127863 | TNFRSF19 | mRNA | -1.103 | 1.025E-06 | -1.389 | 3.400E-06 |
| ENSG00000127884 | ECHS1 | mRNA | -1.316 | 5.400E-12 | -1.098 | 1.814E-06 |
| ENSG00000127947 | PTPN12 | mRNA | 1.374 | 2.565E-07 | 1.143 | 6.653E-04 |
| ENSG00000127951 | FGL2 | mRNA | 2.364 | 8.282E-11 | 2.157 | 1.789E-05 |
| ENSG00000128218 | VPREB3 | mRNA | 2.120 | 4.243E-05 | 1.942 | 1.405E-03 |
| ENSG00000128340 | RAC2 | mRNA | 1.469 | 2.841E-09 | 1.600 | 3.779E-08 |
| ENSG00000128383 | APOBEC3A | mRNA | 2.206 | 6.091E-05 | 2.067 | 4.107E-04 |
| ENSG00000128482 | RNF112 | mRNA | -1.518 | 4.676E-05 | -1.073 | 7.374E-03 |
| ENSG00000128510 | CPA4 | mRNA | 4.986 | 3.916E-13 | 4.693 | 3.950E-08 |
| ENSG00000128655 | PDE11A | mRNA | -1.711 | 5.343E-04 | -2.722 | 1.177E-06 |
| ENSG00000128656 | CHN1 | mRNA | 1.853 | 2.045E-12 | 1.658 | 5.801E-06 |
| ENSG00000128709 | HOXD9 | mRNA | 1.697 | 1.909E-05 | 2.252 | 9.836E-08 |
| ENSG00000128710 | HOXD10 | mRNA | 4.236 | 1.129E-09 | 4.397 | 3.903E-08 |
| ENSG00000128849 | CGNL1 | mRNA | -2.540 | 1.795E-26 | -2.319 | 4.775E-19 |
| ENSG00000128944 | KNSTRN | mRNA | 1.443 | 2.839E-06 | 1.242 | 3.578E-03 |
| ENSG00000129048 | ACKR4 | mRNA | 2.430 | 7.542E-09 | 1.613 | 4.113E-03 |
| ENSG00000129151 | BBOX1 | mRNA | 1.573 | 1.191E-07 | 1.376 | 4.774E-05 |
| ENSG00000129173 | E2F8 | mRNA | 2.118 | 1.688E-12 | 1.280 | 8.331E-04 |
| ENSG00000129226 | CD68 | mRNA | 2.421 | 2.766E-23 | 2.787 | 5.286E-26 |
| ENSG00000129437 | KLK14 | mRNA | -3.406 | 1.033E-06 | -3.109 | 9.535E-06 |
| ENSG00000129521 | EGLN3 | mRNA | 1.768 | 7.047E-07 | 1.964 | 6.214E-08 |
| ENSG00000129596 | CDO1 | mRNA | -1.891 | 8.573E-06 | -1.186 | 4.244E-03 |
| ENSG00000129646 | QRICH2 | mRNA | -1.331 | 4.663E-08 | -1.149 | 1.406E-04 |
| ENSG00000129744 | ART1 | mRNA | -2.171 | 1.459E-04 | -1.651 | 4.592E-03 |
| ENSG00000129810 | SGOL1 | mRNA | 2.988 | 1.148E-08 | 2.020 | 1.407E-03 |
| ENSG00000129910 | CDH15 | mRNA | -4.539 | 1.003E-08 | -2.729 | 4.056E-04 |
| ENSG00000130005 | GAMT | mRNA | -2.149 | 5.408E-16 | -1.217 | 1.070E-05 |
| ENSG00000130032 | PRRG3 | mRNA | -2.654 | 2.968E-12 | -1.934 | 2.568E-05 |
| ENSG00000130037 | KCNA5 | mRNA | -1.198 | 7.370E-03 | 1.215 | 8.349E-03 |
| ENSG00000130038 | EFCAB4B | mRNA | -1.914 | 1.582E-16 | -1.745 | 6.643E-13 |
| ENSG00000130147 | SH3BP4 | mRNA | -1.972 | 1.240E-25 | -1.576 | 5.419E-12 |
| ENSG00000130176 | CNN1 | mRNA | -1.490 | 2.756E-05 | -1.590 | 2.140E-05 |
| ENSG00000130193 | THEM6 | mRNA | -1.783 | 6.085E-12 | -1.057 | 2.277E-03 |
| ENSG00000130208 | APOC1 | mRNA | 2.801 | 1.749E-07 | 3.646 | 3.457E-15 |
| ENSG00000130283 | GDF1 | mRNA | -2.546 | 5.861E-06 | -1.905 | 1.434E-03 |
| ENSG00000130287 | NCAN | mRNA | -2.006 | 3.863E-04 | -1.592 | 4.254E-03 |
| ENSG00000130294 | KIF1A | mRNA | -2.835 | 3.158E-05 | -2.695 | 3.660E-05 |
| ENSG00000130427 | EPO | mRNA | -2.728 | 3.521E-04 | -2.563 | 1.572E-03 |
| ENSG00000130508 | PXDN | mRNA | 1.018 | 5.339E-04 | 1.405 | 2.526E-06 |
| ENSG00000130653 | PNPLA7 | mRNA | -3.182 | 4.213E-36 | -1.639 | 6.400E-06 |
| ENSG00000130701 | RBBP8NL | mRNA | -1.607 | 1.281E-08 | -1.353 | 2.556E-05 |
| ENSG00000130733 | YIPF2 | mRNA | -1.328 | 5.352E-11 | -1.027 | 2.943E-06 |
| ENSG00000130755 | GMFG | mRNA | 1.418 | 3.364E-07 | 1.484 | 1.150E-07 |
| ENSG00000130787 | HIP1R | mRNA | -2.029 | 2.849E-21 | -1.181 | 2.610E-05 |
| ENSG00000130822 | PNCK | mRNA | 1.262 | 3.144E-03 | 2.338 | 1.033E-06 |
| ENSG00000130830 | MPP1 | mRNA | 1.100 | 2.000E-04 | 1.466 | 1.335E-06 |
| ENSG00000130881 | LRP3 | mRNA | -2.260 | 1.824E-19 | -1.137 | 4.676E-04 |
| ENSG00000130988 | RGN | mRNA | -1.739 | 2.499E-08 | -1.390 | 5.739E-06 |
| ENSG00000131015 | ULBP2 | mRNA | 2.594 | 2.211E-08 | 2.610 | 1.688E-04 |
| ENSG00000131042 | LILRB2 | mRNA | 1.934 | 1.613E-11 | 1.980 | 1.927E-09 |
| ENSG00000131080 | EDA2R | mRNA | 1.195 | 8.178E-04 | 1.214 | 5.750E-03 |
| ENSG00000131095 | GFAP | mRNA | -5.780 | 6.044E-22 | -4.335 | 6.603E-12 |
| ENSG00000131097 | HIGD1B | mRNA | 1.680 | 1.997E-06 | 1.952 | 1.186E-06 |
| ENSG00000131188 | PRR7 | mRNA | -1.650 | 6.010E-04 | -1.380 | 7.336E-03 |
| ENSG00000131203 | IDO1 | mRNA | 2.716 | 9.001E-06 | 3.261 | 4.571E-04 |
| ENSG00000131355 | EMR3 | mRNA | 4.199 | 2.355E-16 | 5.515 | 8.188E-21 |
| ENSG00000131389 | SLC6A6 | mRNA | 1.095 | 2.009E-08 | 1.167 | 2.759E-06 |
| ENSG00000131620 | ANO1 | mRNA | -2.347 | 2.543E-14 | -1.816 | 6.798E-10 |
| ENSG00000131747 | TOP2A | mRNA | 3.274 | 3.227E-18 | 2.500 | 8.798E-07 |
| ENSG00000131771 | PPP1R1B | mRNA | -6.695 | 7.165E-51 | -4.606 | 2.277E-24 |
| ENSG00000131781 | FMO5 | mRNA | -1.386 | 1.467E-06 | -1.143 | 3.975E-04 |
| ENSG00000132254 | ARFIP2 | mRNA | -1.146 | 8.400E-09 | -1.063 | 1.778E-06 |
| ENSG00000132297 | HHLA1 | mRNA | 2.092 | 2.193E-07 | 2.345 | 6.448E-06 |
| ENSG00000132334 | PTPRE | mRNA | 1.184 | 9.824E-07 | 1.701 | 3.139E-12 |
| ENSG00000132692 | BCAN | mRNA | -3.931 | 6.521E-08 | -3.421 | 8.247E-07 |
| ENSG00000132702 | HAPLN2 | mRNA | -2.803 | 4.249E-07 | -1.427 | 8.307E-03 |
| ENSG00000132744 | ACY3 | mRNA | -1.230 | 1.446E-03 | -1.858 | 2.437E-05 |
| ENSG00000132846 | ZBED3 | mRNA | -1.349 | 1.547E-08 | -1.036 | 2.262E-04 |
| ENSG00000132854 | KANK4 | mRNA | -3.723 | 2.213E-35 | -2.764 | 4.256E-08 |
| ENSG00000132911 | NMUR2 | mRNA | 2.759 | 2.002E-04 | 4.014 | 4.637E-05 |
| ENSG00000132932 | ATP8A2 | mRNA | 2.198 | 4.129E-09 | 2.184 | 7.808E-07 |
| ENSG00000132938 | MTUS2 | mRNA | -3.313 | 2.109E-16 | -1.956 | 1.312E-06 |
| ENSG00000132958 | TPTE2 | mRNA | 1.662 | 2.369E-03 | 2.772 | 7.924E-04 |
| ENSG00000132965 | ALOX5AP | mRNA | 2.504 | 4.228E-18 | 2.755 | 8.258E-11 |
| ENSG00000133019 | CHRM3 | mRNA | -3.766 | 1.353E-31 | -3.631 | 4.886E-22 |
| ENSG00000133063 | CHIT1 | mRNA | 1.687 | 8.631E-04 | 1.164 | 7.641E-03 |
| ENSG00000133067 | LGR6 | mRNA | -1.173 | 8.388E-05 | -1.435 | 1.582E-06 |
| ENSG00000133107 | TRPC4 | mRNA | 1.915 | 1.346E-06 | 1.967 | 5.936E-07 |
| ENSG00000133110 | POSTN | mRNA | 2.941 | 5.848E-08 | 3.641 | 2.979E-13 |
| ENSG00000133134 | BEX2 | mRNA | -1.178 | 1.625E-05 | -1.538 | 3.641E-07 |
| ENSG00000133135 | RNF128 | mRNA | -3.920 | 7.816E-21 | -3.973 | 1.244E-21 |
| ENSG00000133315 | MACROD1 | mRNA | -1.607 | 1.104E-12 | -1.066 | 1.829E-04 |
| ENSG00000133317 | LGALS12 | mRNA | 4.663 | 2.431E-08 | 6.029 | 2.131E-19 |
| ENSG00000133392 | MYH11 | mRNA | -2.140 | 5.528E-12 | -1.950 | 4.580E-08 |
| ENSG00000133574 | GIMAP4 | mRNA | 1.777 | 6.808E-08 | 1.279 | 1.747E-03 |
| ENSG00000133661 | SFTPD | mRNA | -2.842 | 4.554E-13 | -1.952 | 1.653E-05 |
| ENSG00000133800 | LYVE1 | mRNA | 1.687 | 1.267E-03 | 3.815 | 1.242E-11 |
| ENSG00000133816 | MICAL2 | mRNA | 1.194 | 1.042E-10 | 1.145 | 2.889E-07 |
| ENSG00000133863 | TEX15 | mRNA | 2.139 | 6.645E-03 | 2.881 | 1.049E-03 |
| ENSG00000134007 | ADAM20 | mRNA | 1.193 | 2.142E-03 | 1.216 | 2.369E-03 |
| ENSG00000134028 | ADAMDEC1 | mRNA | 3.848 | 6.880E-13 | 1.865 | 2.060E-03 |
| ENSG00000134057 | CCNB1 | mRNA | 2.026 | 3.425E-10 | 1.602 | 2.223E-04 |
| ENSG00000134193 | REG4 | mRNA | -5.677 | 1.757E-10 | -3.598 | 5.962E-06 |
| ENSG00000134207 | SYT6 | mRNA | -2.165 | 9.078E-04 | -2.082 | 2.425E-03 |
| ENSG00000134215 | VAV3 | mRNA | 2.173 | 3.169E-16 | 1.296 | 4.288E-05 |
| ENSG00000134240 | HMGCS2 | mRNA | -5.116 | 1.526E-22 | -2.959 | 4.664E-07 |
| ENSG00000134242 | PTPN22 | mRNA | 2.327 | 5.500E-09 | 1.893 | 2.047E-04 |
| ENSG00000134256 | CD101 | mRNA | 2.202 | 5.016E-12 | 1.540 | 6.494E-04 |
| ENSG00000134323 | MYCN | mRNA | -2.798 | 4.392E-13 | -2.519 | 2.681E-07 |
| ENSG00000134333 | LDHA | mRNA | 1.603 | 4.133E-11 | 1.407 | 1.397E-05 |
| ENSG00000134460 | IL2RA | mRNA | 3.357 | 1.089E-14 | 4.289 | 3.008E-22 |
| ENSG00000134463 | ECHDC3 | mRNA | -2.331 | 2.499E-15 | -1.031 | 2.293E-03 |
| ENSG00000134489 | HRH4 | mRNA | 2.792 | 7.157E-05 | 4.188 | 3.183E-08 |
| ENSG00000134516 | DOCK2 | mRNA | 1.423 | 7.820E-09 | 1.619 | 1.305E-09 |
| ENSG00000134551 | PRH2 | mRNA | -3.125 | 1.396E-03 | -4.104 | 7.871E-05 |
| ENSG00000134602 | MST4 | mRNA | 1.688 | 7.264E-08 | 1.174 | 4.285E-03 |
| ENSG00000134690 | CDCA8 | mRNA | 1.391 | 3.938E-04 | 1.116 | 6.060E-03 |
| ENSG00000134716 | CYP2J2 | mRNA | -1.510 | 2.559E-10 | -1.041 | 4.149E-03 |
| ENSG00000134757 | DSG3 | mRNA | 3.009 | 1.742E-10 | 3.240 | 8.096E-08 |
| ENSG00000134762 | DSC3 | mRNA | 1.770 | 5.584E-10 | 1.453 | 8.519E-04 |
| ENSG00000134827 | TCN1 | mRNA | -3.697 | 6.748E-11 | -3.214 | 6.426E-07 |
| ENSG00000134853 | PDGFRA | mRNA | 1.439 | 2.614E-09 | 1.862 | 1.446E-10 |
| ENSG00000134955 | SLC37A2 | mRNA | -2.442 | 1.492E-18 | -1.798 | 5.135E-08 |
| ENSG00000134986 | NREP | mRNA | 1.725 | 1.712E-12 | 1.666 | 1.991E-06 |
| ENSG00000135074 | ADAM19 | mRNA | 1.121 | 2.003E-05 | 1.626 | 9.054E-08 |
| ENSG00000135114 | OASL | mRNA | 1.548 | 4.339E-05 | 1.813 | 1.327E-03 |
| ENSG00000135218 | CD36 | mRNA | 1.853 | 4.778E-06 | 1.985 | 1.787E-05 |
| ENSG00000135312 | HTR1B | mRNA | 1.579 | 1.067E-03 | 1.949 | 8.385E-04 |
| ENSG00000135333 | EPHA7 | mRNA | -2.999 | 1.293E-10 | -2.883 | 2.677E-07 |
| ENSG00000135362 | PRR5L | mRNA | 1.064 | 1.010E-06 | 1.422 | 5.845E-09 |
| ENSG00000135373 | EHF | mRNA | -1.232 | 3.337E-04 | -1.481 | 2.657E-04 |
| ENSG00000135374 | ELF5 | mRNA | -2.500 | 6.249E-15 | -1.934 | 5.367E-08 |
| ENSG00000135406 | PRPH | mRNA | -3.604 | 6.906E-12 | -2.774 | 7.762E-06 |
| ENSG00000135409 | AMHR2 | mRNA | 2.723 | 8.348E-04 | 3.046 | 3.677E-05 |
| ENSG00000135426 | TESPA1 | mRNA | 2.139 | 3.363E-10 | 2.136 | 2.519E-09 |
| ENSG00000135451 | TROAP | mRNA | 2.007 | 1.115E-05 | 1.582 | 1.734E-03 |
| ENSG00000135454 | B4GALNT1 | mRNA | -1.212 | 2.459E-04 | -1.503 | 9.281E-06 |
| ENSG00000135472 | FAIM2 | mRNA | -1.206 | 4.259E-04 | -1.740 | 8.688E-07 |
| ENSG00000135480 | KRT7 | mRNA | -1.838 | 1.810E-11 | -1.568 | 1.697E-04 |
| ENSG00000135519 | KCNH3 | mRNA | -1.439 | 1.227E-02 | -1.582 | 5.800E-03 |
| ENSG00000135678 | CPM | mRNA | 1.005 | 1.314E-03 | 1.857 | 3.425E-07 |
| ENSG00000135744 | AGT | mRNA | -1.803 | 5.028E-07 | -1.536 | 3.592E-04 |
| ENSG00000135773 | CAPN9 | mRNA | -2.043 | 2.875E-08 | -1.153 | 2.136E-03 |
| ENSG00000135838 | NPL | mRNA | 2.548 | 6.074E-23 | 2.603 | 7.828E-20 |
| ENSG00000135898 | GPR55 | mRNA | -1.660 | 4.342E-03 | -1.644 | 6.012E-03 |
| ENSG00000135914 | HTR2B | mRNA | 2.412 | 7.422E-06 | 2.729 | 3.590E-07 |
| ENSG00000135929 | CYP27A1 | mRNA | -2.097 | 3.534E-21 | -1.068 | 6.554E-05 |
| ENSG00000136011 | STAB2 | mRNA | -3.013 | 1.761E-07 | -2.347 | 2.943E-06 |
| ENSG00000136040 | PLXNC1 | mRNA | 1.549 | 1.652E-11 | 1.475 | 1.250E-07 |
| ENSG00000136048 | DRAM1 | mRNA | 1.399 | 9.672E-09 | 1.098 | 2.526E-03 |
| ENSG00000136099 | PCDH8 | mRNA | -4.068 | 3.786E-06 | -2.623 | 3.540E-03 |
| ENSG00000136155 | SCEL | mRNA | 3.930 | 4.378E-09 | 3.071 | 2.813E-05 |
| ENSG00000136167 | LCP1 | mRNA | 1.727 | 8.137E-11 | 1.534 | 3.557E-06 |
| ENSG00000136235 | GPNMB | mRNA | 1.882 | 3.448E-14 | 1.884 | 5.665E-11 |
| ENSG00000136250 | AOAH | mRNA | 1.757 | 1.287E-10 | 1.338 | 4.896E-05 |
| ENSG00000136404 | TM6SF1 | mRNA | 1.497 | 2.420E-06 | 1.612 | 1.491E-05 |
| ENSG00000136457 | CHAD | mRNA | -2.389 | 3.247E-07 | -1.047 | 3.149E-03 |
| ENSG00000136541 | ERMN | mRNA | 1.311 | 6.062E-04 | 1.386 | 1.666E-03 |
| ENSG00000136546 | SCN7A | mRNA | -4.046 | 2.519E-09 | -3.400 | 7.273E-07 |
| ENSG00000136574 | GATA4 | mRNA | -4.248 | 2.497E-06 | -4.131 | 8.328E-06 |
| ENSG00000136630 | HLX | mRNA | 1.075 | 7.204E-04 | 1.849 | 3.245E-09 |
| ENSG00000136634 | IL10 | mRNA | 2.516 | 1.865E-07 | 3.648 | 1.428E-11 |
| ENSG00000136867 | SLC31A2 | mRNA | -2.065 | 7.774E-11 | -1.887 | 4.591E-07 |
| ENSG00000136869 | TLR4 | mRNA | 1.238 | 1.977E-04 | 1.085 | 3.919E-03 |
| ENSG00000136883 | KIF12 | mRNA | -3.420 | 4.037E-16 | -1.518 | 9.843E-04 |
| ENSG00000136895 | GARNL3 | mRNA | -1.316 | 2.704E-08 | -1.158 | 3.538E-05 |
| ENSG00000136960 | ENPP2 | mRNA | 1.421 | 2.765E-08 | 1.261 | 1.769E-05 |
| ENSG00000137103 | TMEM8B | mRNA | -1.586 | 1.097E-13 | -1.030 | 7.740E-05 |
| ENSG00000137193 | PIM1 | mRNA | 1.041 | 1.643E-04 | 1.815 | 4.608E-10 |
| ENSG00000137198 | GMPR | mRNA | -2.490 | 1.321E-32 | -1.698 | 2.322E-09 |
| ENSG00000137259 | HIST1H2AB | mRNA | 2.432 | 3.199E-08 | 1.943 | 7.328E-07 |
| ENSG00000137265 | IRF4 | mRNA | 1.355 | 3.053E-05 | 1.522 | 5.517E-04 |
| ENSG00000137440 | FGFBP1 | mRNA | 3.223 | 9.172E-09 | 2.906 | 2.470E-06 |
| ENSG00000137462 | TLR2 | mRNA | 1.179 | 3.453E-06 | 1.231 | 3.587E-03 |
| ENSG00000137463 | MGARP | mRNA | 2.527 | 4.069E-08 | 2.741 | 1.893E-09 |
| ENSG00000137474 | MYO7A | mRNA | 1.034 | 2.659E-03 | 1.628 | 2.295E-06 |
| ENSG00000137491 | SLCO2B1 | mRNA | 1.094 | 2.025E-06 | 1.852 | 2.914E-12 |
| ENSG00000137573 | SULF1 | mRNA | 2.530 | 6.902E-26 | 2.607 | 3.992E-15 |
| ENSG00000137672 | TRPC6 | mRNA | 1.744 | 1.286E-09 | 2.112 | 1.481E-10 |
| ENSG00000137675 | MMP27 | mRNA | 1.821 | 1.945E-03 | 1.891 | 1.541E-03 |
| ENSG00000137804 | NUSAP1 | mRNA | 3.278 | 1.366E-15 | 2.482 | 4.941E-06 |
| ENSG00000137807 | KIF23 | mRNA | 3.276 | 4.943E-15 | 2.633 | 1.358E-05 |
| ENSG00000137809 | ITGA11 | mRNA | 1.167 | 3.546E-05 | 2.025 | 4.328E-08 |
| ENSG00000137812 | CASC5 | mRNA | 1.992 | 3.098E-08 | 1.398 | 1.053E-03 |
| ENSG00000137819 | PAQR5 | mRNA | 1.347 | 2.770E-04 | 1.493 | 4.885E-05 |
| ENSG00000137878 | GCOM1 | mRNA | -1.440 | 2.162E-06 | -1.118 | 1.156E-03 |
| ENSG00000137968 | SLC44A5 | mRNA | -1.769 | 2.274E-03 | -2.569 | 3.357E-04 |
| ENSG00000137975 | CLCA2 | mRNA | 3.021 | 3.312E-08 | 1.917 | 7.408E-03 |
| ENSG00000138028 | CGREF1 | mRNA | -6.682 | 2.484E-24 | -4.899 | 2.583E-17 |
| ENSG00000138101 | DTNB | mRNA | -1.247 | 7.633E-10 | -1.068 | 9.302E-06 |
| ENSG00000138119 | MYOF | mRNA | 1.137 | 1.022E-09 | 1.002 | 2.022E-05 |
| ENSG00000138160 | KIF11 | mRNA | 2.181 | 2.626E-13 | 1.409 | 3.526E-04 |
| ENSG00000138162 | TACC2 | mRNA | -1.677 | 8.324E-11 | -1.129 | 6.695E-04 |
| ENSG00000138180 | CEP55 | mRNA | 3.156 | 4.703E-10 | 2.545 | 7.307E-05 |
| ENSG00000138185 | ENTPD1 | mRNA | 1.438 | 1.218E-07 | 1.280 | 2.385E-04 |
| ENSG00000138207 | RBP4 | mRNA | -2.614 | 4.125E-03 | 2.795 | 8.125E-04 |
| ENSG00000138271 | GPR87 | mRNA | 2.850 | 1.818E-15 | 2.131 | 1.976E-06 |
| ENSG00000138378 | STAT4 | mRNA | 1.585 | 7.933E-07 | 1.138 | 1.269E-03 |
| ENSG00000138395 | CDK15 | mRNA | 2.011 | 1.282E-06 | 2.321 | 1.549E-07 |
| ENSG00000138435 | CHRNA1 | mRNA | 4.568 | 3.105E-08 | 4.979 | 3.288E-06 |
| ENSG00000138615 | CILP | mRNA | -1.566 | 2.320E-07 | -1.199 | 9.807E-04 |
| ENSG00000138744 | NAAA | mRNA | -1.481 | 1.305E-12 | -1.294 | 5.705E-08 |
| ENSG00000138756 | BMP2K | mRNA | 1.602 | 2.975E-10 | 1.134 | 8.284E-04 |
| ENSG00000138771 | SHROOM3 | mRNA | -1.107 | 8.766E-06 | -1.076 | 7.403E-07 |
| ENSG00000138778 | CENPE | mRNA | 3.767 | 9.542E-18 | 3.103 | 6.556E-07 |
| ENSG00000138792 | ENPEP | mRNA | 1.118 | 4.200E-04 | 1.466 | 1.896E-04 |
| ENSG00000138798 | EGF | mRNA | -4.025 | 6.856E-24 | -4.720 | 6.151E-24 |
| ENSG00000138821 | SLC39A8 | mRNA | -1.605 | 2.234E-09 | -1.180 | 1.266E-04 |
| ENSG00000139055 | ERP27 | mRNA | 1.843 | 9.912E-07 | 1.344 | 1.455E-03 |
| ENSG00000139117 | CPNE8 | mRNA | 1.455 | 2.031E-08 | 1.220 | 4.418E-04 |
| ENSG00000139187 | KLRG1 | mRNA | 1.546 | 1.764E-06 | 1.348 | 1.503E-04 |
| ENSG00000139278 | GLIPR1 | mRNA | 2.804 | 7.289E-11 | 1.786 | 1.074E-03 |
| ENSG00000139289 | PHLDA1 | mRNA | -2.560 | 4.157E-22 | -2.524 | 3.145E-18 |
| ENSG00000139292 | LGR5 | mRNA | 1.285 | 1.655E-02 | 2.202 | 1.651E-05 |
| ENSG00000139304 | PTPRQ | mRNA | 3.114 | 1.365E-06 | 4.380 | 5.107E-09 |
| ENSG00000139324 | TMTC3 | mRNA | 2.275 | 1.236E-09 | 1.539 | 2.464E-03 |
| ENSG00000139329 | LUM | mRNA | 2.236 | 4.188E-10 | 1.861 | 2.411E-04 |
| ENSG00000139572 | GPR84 | mRNA | 2.933 | 1.924E-06 | 2.615 | 1.096E-04 |
| ENSG00000139618 | BRCA2 | mRNA | 2.377 | 2.202E-10 | 1.449 | 2.234E-03 |
| ENSG00000139629 | GALNT6 | mRNA | -2.682 | 4.295E-25 | -2.334 | 3.908E-14 |
| ENSG00000139734 | DIAPH3 | mRNA | 2.299 | 5.001E-10 | 1.788 | 1.840E-05 |
| ENSG00000139737 | SLAIN1 | mRNA | -2.383 | 1.892E-15 | -2.331 | 2.739E-11 |
| ENSG00000139874 | SSTR1 | mRNA | -1.093 | 4.150E-03 | -1.312 | 8.245E-04 |
| ENSG00000139970 | RTN1 | mRNA | -3.252 | 2.713E-28 | -3.158 | 6.768E-22 |
| ENSG00000140030 | GPR65 | mRNA | 3.103 | 1.538E-11 | 3.027 | 1.639E-06 |
| ENSG00000140067 | FAM181A | mRNA | -2.037 | 5.146E-03 | -3.254 | 1.542E-06 |
| ENSG00000140104 | C14orf79 | mRNA | -1.223 | 4.878E-04 | -1.034 | 4.351E-03 |
| ENSG00000140274 | DUOXA2 | mRNA | 4.263 | 2.321E-07 | 3.037 | 2.012E-03 |
| ENSG00000140285 | FGF7 | mRNA | 2.340 | 3.582E-07 | 1.764 | 1.459E-03 |
| ENSG00000140287 | HDC | mRNA | 1.308 | 9.012E-05 | 1.689 | 1.711E-07 |
| ENSG00000140379 | BCL2A1 | mRNA | 3.870 | 3.861E-16 | 4.640 | 7.167E-12 |
| ENSG00000140470 | ADAMTS17 | mRNA | -1.895 | 6.352E-15 | -1.254 | 1.424E-05 |
| ENSG00000140479 | PCSK6 | mRNA | 1.075 | 9.163E-03 | 2.229 | 8.759E-10 |
| ENSG00000140481 | CCDC33 | mRNA | -1.667 | 3.398E-03 | -1.578 | 5.578E-03 |
| ENSG00000140506 | LMAN1L | mRNA | -5.496 | 7.106E-09 | -3.065 | 2.822E-05 |
| ENSG00000140519 | RHCG | mRNA | 3.405 | 1.832E-04 | 5.148 | 1.832E-06 |
| ENSG00000140534 | TICRR | mRNA | 2.041 | 7.080E-06 | 1.769 | 4.622E-04 |
| ENSG00000140678 | ITGAX | mRNA | 1.543 | 1.278E-07 | 2.114 | 1.781E-12 |
| ENSG00000140749 | IGSF6 | mRNA | 2.667 | 1.535E-19 | 2.293 | 6.395E-11 |
| ENSG00000140932 | CMTM2 | mRNA | 1.801 | 1.212E-02 | 2.000 | 5.885E-03 |
| ENSG00000140945 | CDH13 | mRNA | 1.425 | 2.292E-05 | 1.448 | 7.882E-05 |
| ENSG00000141448 | GATA6 | mRNA | -1.629 | 4.284E-08 | -1.078 | 1.044E-03 |
| ENSG00000141469 | SLC14A1 | mRNA | -1.798 | 3.977E-06 | -3.070 | 9.725E-13 |
| ENSG00000141480 | ARRB2 | mRNA | 1.162 | 2.190E-08 | 1.549 | 1.633E-13 |
| ENSG00000141655 | TNFRSF11A | mRNA | -2.424 | 3.977E-31 | -2.411 | 2.567E-19 |
| ENSG00000141837 | CACNA1A | mRNA | -1.742 | 2.012E-04 | -1.753 | 1.285E-04 |
| ENSG00000141854 | hsa-mir-1199 | mRNA | -2.095 | 7.994E-09 | -1.281 | 1.127E-03 |
| ENSG00000141934 | PPAP2C | mRNA | -1.467 | 1.557E-10 | -1.202 | 1.117E-05 |
| ENSG00000142089 | IFITM3 | mRNA | 1.373 | 1.682E-12 | 1.309 | 1.300E-05 |
| ENSG00000142197 | DOPEY2 | mRNA | -1.393 | 3.156E-13 | -1.234 | 2.409E-08 |
| ENSG00000142227 | EMP3 | mRNA | 1.115 | 2.583E-06 | 1.328 | 1.983E-07 |
| ENSG00000142347 | MYO1F | mRNA | 1.106 | 7.813E-05 | 1.597 | 7.846E-08 |
| ENSG00000142405 | NLRP12 | mRNA | 1.520 | 2.393E-03 | 2.823 | 9.873E-09 |
| ENSG00000142449 | FBN3 | mRNA | -4.878 | 1.611E-16 | -2.741 | 2.793E-05 |
| ENSG00000142512 | SIGLEC10 | mRNA | 1.880 | 1.084E-07 | 2.996 | 1.494E-12 |
| ENSG00000142530 | FAM71E1 | mRNA | -1.387 | 5.573E-05 | -1.304 | 1.510E-04 |
| ENSG00000142583 | SLC2A5 | mRNA | 1.264 | 1.901E-04 | 2.456 | 1.098E-09 |
| ENSG00000142619 | PADI3 | mRNA | 1.661 | 2.044E-03 | 1.648 | 2.687E-03 |
| ENSG00000142632 | ARHGEF19 | mRNA | -1.804 | 8.884E-12 | -1.060 | 5.011E-04 |
| ENSG00000142669 | SH3BGRL3 | mRNA | 1.154 | 8.785E-08 | 1.106 | 3.731E-06 |
| ENSG00000142731 | PLK4 | mRNA | 2.966 | 1.533E-15 | 1.813 | 5.278E-05 |
| ENSG00000142945 | KIF2C | mRNA | 1.905 | 3.073E-06 | 1.606 | 3.269E-04 |
| ENSG00000142959 | BEST4 | mRNA | -2.030 | 1.499E-04 | -2.135 | 6.956E-05 |
| ENSG00000143001 | TMEM61 | mRNA | -1.738 | 1.903E-06 | -1.365 | 4.417E-04 |
| ENSG00000143110 | C1orf162 | mRNA | 2.168 | 1.481E-08 | 2.638 | 1.469E-08 |
| ENSG00000143119 | CD53 | mRNA | 2.203 | 1.325E-13 | 1.951 | 2.136E-07 |
| ENSG00000143140 | GJA5 | mRNA | 1.737 | 4.680E-07 | 1.740 | 2.401E-06 |
| ENSG00000143171 | RXRG | mRNA | -2.228 | 3.100E-06 | -3.076 | 4.002E-08 |
| ENSG00000143226 | FCGR2A | mRNA | 2.297 | 5.336E-16 | 2.467 | 1.430E-19 |
| ENSG00000143228 | NUF2 | mRNA | 2.949 | 5.673E-10 | 2.100 | 9.303E-04 |
| ENSG00000143341 | HMCN1 | mRNA | 1.279 | 7.642E-08 | 1.011 | 3.731E-03 |
| ENSG00000143365 | RORC | mRNA | -2.209 | 2.561E-22 | -2.007 | 3.857E-14 |
| ENSG00000143369 | ECM1 | mRNA | 1.488 | 1.430E-09 | 1.840 | 2.957E-10 |
| ENSG00000143416 | SELENBP1 | mRNA | -1.237 | 6.736E-09 | -1.223 | 4.131E-07 |
| ENSG00000143450 | OAZ3 | mRNA | -1.297 | 9.592E-05 | -1.339 | 7.685E-05 |
| ENSG00000143476 | DTL | mRNA | 2.785 | 3.359E-13 | 1.774 | 4.714E-05 |
| ENSG00000143502 | SUSD4 | mRNA | -1.151 | 4.840E-08 | -1.641 | 6.617E-08 |
| ENSG00000143546 | S100A8 | mRNA | 5.570 | 8.794E-24 | 5.407 | 6.338E-15 |
| ENSG00000143578 | CREB3L4 | mRNA | -1.866 | 4.948E-13 | -1.563 | 1.019E-09 |
| ENSG00000143768 | LEFTY2 | mRNA | -1.696 | 1.894E-03 | 2.491 | 2.890E-03 |
| ENSG00000143797 | MBOAT2 | mRNA | 1.881 | 5.121E-10 | 1.017 | 4.173E-03 |
| ENSG00000143842 | SOX13 | mRNA | -1.616 | 2.250E-16 | -1.106 | 1.888E-05 |
| ENSG00000143851 | PTPN7 | mRNA | 1.536 | 2.720E-07 | 1.893 | 1.782E-08 |
| ENSG00000143858 | SYT2 | mRNA | 2.210 | 3.325E-06 | 2.134 | 4.716E-07 |
| ENSG00000143867 | OSR1 | mRNA | -1.675 | 4.764E-09 | -1.109 | 1.437E-04 |
| ENSG00000144031 | ANKRD53 | mRNA | -1.386 | 4.791E-04 | -1.189 | 1.048E-03 |
| ENSG00000144045 | DQX1 | mRNA | 1.683 | 1.071E-04 | 2.027 | 4.580E-07 |
| ENSG00000144199 | FAHD2B | mRNA | -1.093 | 1.238E-05 | -1.044 | 2.604E-04 |
| ENSG00000144229 | THSD7B | mRNA | -1.541 | 1.289E-03 | -1.173 | 3.487E-03 |
| ENSG00000144230 | GPR17 | mRNA | -2.934 | 4.304E-04 | -2.046 | 8.295E-03 |
| ENSG00000144278 | GALNT13 | mRNA | -5.121 | 2.093E-27 | -4.584 | 4.152E-15 |
| ENSG00000144331 | ZNF385B | mRNA | -2.344 | 2.017E-04 | -4.395 | 1.526E-09 |
| ENSG00000144369 | FAM171B | mRNA | -1.236 | 7.359E-05 | -1.689 | 5.215E-06 |
| ENSG00000144406 | UNC80 | mRNA | -3.077 | 1.690E-14 | -2.741 | 2.278E-12 |
| ENSG00000144407 | PTH2R | mRNA | -3.153 | 5.387E-15 | -2.397 | 1.957E-09 |
| ENSG00000144452 | ABCA12 | mRNA | 3.114 | 1.969E-09 | 1.658 | 6.124E-03 |
| ENSG00000144681 | STAC | mRNA | 1.018 | 7.000E-03 | 1.466 | 6.383E-04 |
| ENSG00000144785 | RP11-977G19.10 | mRNA | -1.560 | 2.310E-04 | -1.262 | 2.529E-03 |
| ENSG00000144810 | COL8A1 | mRNA | 1.226 | 8.666E-06 | 1.925 | 1.249E-10 |
| ENSG00000144824 | PHLDB2 | mRNA | 1.068 | 6.038E-05 | 1.189 | 3.145E-04 |
| ENSG00000144837 | PLA1A | mRNA | 1.533 | 9.720E-06 | 1.206 | 1.586E-03 |
| ENSG00000144847 | IGSF11 | mRNA | -1.369 | 4.785E-05 | -1.936 | 3.740E-05 |
| ENSG00000144852 | NR1I2 | mRNA | -1.185 | 3.237E-03 | -1.405 | 1.159E-03 |
| ENSG00000144908 | ALDH1L1 | mRNA | -2.926 | 2.706E-29 | -2.410 | 3.728E-10 |
| ENSG00000145087 | STXBP5L | mRNA | -3.743 | 5.167E-06 | -3.932 | 4.013E-06 |
| ENSG00000145216 | FIP1L1 | mRNA | 1.121 | 4.137E-09 | 1.361 | 1.213E-08 |
| ENSG00000145217 | SLC26A1 | mRNA | -2.144 | 2.313E-06 | -1.303 | 3.477E-03 |
| ENSG00000145386 | CCNA2 | mRNA | 2.757 | 4.158E-09 | 2.122 | 1.359E-04 |
| ENSG00000145416 | 43525.000 | mRNA | 3.153 | 3.526E-14 | 2.579 | 1.165E-06 |
| ENSG00000145431 | PDGFC | mRNA | -1.383 | 6.661E-14 | -1.268 | 2.117E-07 |
| ENSG00000145623 | OSMR | mRNA | 1.634 | 8.169E-16 | 1.583 | 7.814E-10 |
| ENSG00000145649 | GZMA | mRNA | 2.469 | 3.097E-07 | 1.552 | 5.086E-03 |
| ENSG00000145681 | HAPLN1 | mRNA | 2.495 | 2.514E-05 | 3.187 | 1.668E-07 |
| ENSG00000145708 | CRHBP | mRNA | -3.680 | 1.334E-08 | -3.460 | 2.342E-06 |
| ENSG00000145777 | TSLP | mRNA | 2.091 | 6.242E-08 | 1.695 | 3.604E-03 |
| ENSG00000145779 | TNFAIP8 | mRNA | 1.520 | 1.842E-10 | 1.388 | 1.182E-06 |
| ENSG00000145808 | ADAMTS19 | mRNA | -3.078 | 1.579E-09 | -3.490 | 1.139E-10 |
| ENSG00000145832 | SLC25A48 | mRNA | -5.158 | 9.164E-19 | -3.282 | 1.994E-09 |
| ENSG00000145850 | TIMD4 | mRNA | 2.816 | 9.964E-08 | 5.435 | 7.415E-10 |
| ENSG00000145920 | CPLX2 | mRNA | 2.390 | 1.514E-02 | 3.201 | 1.848E-04 |
| ENSG00000145936 | KCNMB1 | mRNA | -1.342 | 6.671E-05 | -1.390 | 1.954E-04 |
| ENSG00000146070 | PLA2G7 | mRNA | 4.045 | 4.016E-23 | 3.505 | 5.346E-13 |
| ENSG00000146094 | DOK3 | mRNA | 1.295 | 4.108E-06 | 1.924 | 5.624E-09 |
| ENSG00000146477 | SLC22A3 | mRNA | -4.447 | 1.215E-45 | -4.023 | 5.800E-20 |
| ENSG00000146521 | C6orf123 | mRNA | -1.687 | 1.317E-04 | -1.669 | 3.237E-04 |
| ENSG00000146530 | VWDE | mRNA | -1.584 | 1.401E-06 | -2.103 | 4.169E-08 |
| ENSG00000146592 | CREB5 | mRNA | 1.915 | 3.063E-12 | 1.134 | 4.809E-03 |
| ENSG00000146670 | CDCA5 | mRNA | 1.427 | 8.495E-04 | 1.153 | 7.238E-03 |
| ENSG00000146674 | IGFBP3 | mRNA | 2.057 | 2.114E-06 | 1.858 | 2.915E-05 |
| ENSG00000147138 | GPR174 | mRNA | 2.203 | 1.135E-07 | 1.320 | 6.090E-03 |
| ENSG00000147234 | FRMPD3 | mRNA | -2.603 | 5.137E-12 | -1.798 | 7.859E-06 |
| ENSG00000147251 | DOCK11 | mRNA | 1.594 | 1.365E-11 | 1.490 | 7.818E-08 |
| ENSG00000147255 | IGSF1 | mRNA | -3.884 | 2.663E-13 | -3.145 | 7.950E-08 |
| ENSG00000147402 | GABRQ | mRNA | 2.540 | 1.139E-05 | 2.578 | 6.568E-04 |
| ENSG00000147443 | DOK2 | mRNA | 1.050 | 3.079E-03 | 1.906 | 1.858E-07 |
| ENSG00000147488 | ST18 | mRNA | 3.432 | 5.439E-12 | 2.923 | 4.315E-08 |
| ENSG00000147588 | PMP2 | mRNA | -6.637 | 9.941E-10 | -6.518 | 4.063E-09 |
| ENSG00000147689 | FAM83A | mRNA | 2.330 | 2.530E-06 | 2.760 | 1.652E-04 |
| ENSG00000147697 | GSDMC | mRNA | 1.520 | 1.038E-08 | 1.637 | 1.124E-07 |
| ENSG00000147799 | ARHGAP39 | mRNA | -1.521 | 3.953E-04 | -1.236 | 4.615E-03 |
| ENSG00000148175 | STOM | mRNA | 1.161 | 1.783E-07 | 1.297 | 1.596E-04 |
| ENSG00000148204 | CRB2 | mRNA | -3.103 | 1.239E-09 | -2.179 | 1.397E-04 |
| ENSG00000148219 | ASTN2 | mRNA | -1.750 | 2.808E-12 | -1.479 | 7.775E-09 |
| ENSG00000148468 | FAM171A1 | mRNA | -1.355 | 1.778E-13 | -1.325 | 2.554E-09 |
| ENSG00000148483 | TMEM236 | mRNA | 2.003 | 6.440E-03 | 2.924 | 2.418E-04 |
| ENSG00000148604 | RGR | mRNA | -4.194 | 8.277E-11 | -3.196 | 1.865E-07 |
| ENSG00000148773 | MKI67 | mRNA | 3.241 | 1.332E-15 | 2.626 | 2.105E-08 |
| ENSG00000149021 | SCGB1A1 | mRNA | -3.636 | 9.041E-07 | -3.269 | 1.562E-05 |
| ENSG00000149243 | KLHL35 | mRNA | -2.095 | 3.241E-13 | -1.657 | 4.490E-05 |
| ENSG00000149294 | NCAM1 | mRNA | -1.409 | 1.325E-04 | -1.464 | 2.112E-04 |
| ENSG00000149328 | GLB1L2 | mRNA | -1.740 | 6.381E-11 | -1.919 | 2.161E-10 |
| ENSG00000149452 | SLC22A8 | mRNA | -1.970 | 6.524E-03 | -2.964 | 4.173E-05 |
| ENSG00000149534 | MS4A2 | mRNA | 2.932 | 7.894E-12 | 2.683 | 9.249E-08 |
| ENSG00000149571 | KIRREL3 | mRNA | -3.140 | 5.047E-16 | -1.513 | 4.896E-04 |
| ENSG00000149596 | JPH2 | mRNA | -2.235 | 1.032E-14 | -1.368 | 1.255E-04 |
| ENSG00000149809 | TM7SF2 | mRNA | -1.872 | 9.267E-17 | -1.879 | 9.644E-17 |
| ENSG00000149948 | HMGA2 | mRNA | 1.991 | 2.573E-06 | 1.853 | 2.361E-05 |
| ENSG00000149968 | MMP3 | mRNA | 4.082 | 1.071E-05 | 2.668 | 4.714E-04 |
| ENSG00000150165 | ANXA8L1 | mRNA | 1.864 | 1.389E-07 | 2.195 | 4.924E-09 |
| ENSG00000150275 | PCDH15 | mRNA | -2.677 | 3.512E-03 | -3.829 | 1.159E-05 |
| ENSG00000150337 | FCGR1A | mRNA | 3.513 | 1.836E-13 | 3.297 | 1.124E-05 |
| ENSG00000150556 | LYPD6B | mRNA | 1.054 | 7.585E-05 | 1.035 | 1.974E-04 |
| ENSG00000150625 | GPM6A | mRNA | -1.589 | 1.630E-05 | -2.626 | 8.838E-09 |
| ENSG00000150636 | CCDC102B | mRNA | 2.388 | 1.589E-10 | 1.717 | 4.092E-04 |
| ENSG00000150672 | DLG2 | mRNA | -1.163 | 1.490E-04 | -1.900 | 3.770E-08 |
| ENSG00000150681 | RGS18 | mRNA | 3.772 | 1.458E-18 | 4.034 | 6.199E-09 |
| ENSG00000151012 | SLC7A11 | mRNA | 2.219 | 1.007E-12 | 1.526 | 1.563E-04 |
| ENSG00000151364 | KCTD14 | mRNA | -1.869 | 6.210E-11 | -2.146 | 1.630E-11 |
| ENSG00000151388 | ADAMTS12 | mRNA | 1.591 | 2.822E-07 | 2.142 | 1.232E-10 |
| ENSG00000151490 | PTPRO | mRNA | 1.678 | 1.208E-06 | 1.762 | 2.225E-05 |
| ENSG00000151651 | ADAM8 | mRNA | 1.001 | 1.465E-03 | 2.108 | 2.886E-08 |
| ENSG00000151702 | FLI1 | mRNA | 1.250 | 2.125E-06 | 1.186 | 5.454E-05 |
| ENSG00000151704 | KCNJ1 | mRNA | 1.274 | 1.946E-02 | 1.570 | 8.327E-04 |
| ENSG00000151746 | BICD1 | mRNA | 1.156 | 1.977E-09 | 1.107 | 2.072E-06 |
| ENSG00000151790 | TDO2 | mRNA | 4.490 | 6.289E-13 | 2.366 | 4.666E-05 |
| ENSG00000151834 | GABRA2 | mRNA | -3.481 | 5.164E-07 | -4.200 | 8.207E-08 |
| ENSG00000151882 | CCL28 | mRNA | -3.017 | 2.294E-14 | -3.403 | 4.411E-17 |
| ENSG00000151948 | GLT1D1 | mRNA | 1.932 | 3.334E-05 | 1.642 | 1.003E-03 |
| ENSG00000152049 | KCNE4 | mRNA | 1.359 | 6.009E-07 | 1.305 | 2.276E-05 |
| ENSG00000152078 | TMEM56 | mRNA | -1.353 | 1.722E-02 | -1.995 | 3.573E-04 |
| ENSG00000152207 | CYSLTR2 | mRNA | 2.633 | 1.215E-10 | 3.153 | 6.865E-11 |
| ENSG00000152208 | GRID2 | mRNA | -2.528 | 2.230E-03 | -2.583 | 3.325E-03 |
| ENSG00000152213 | ARL11 | mRNA | 1.238 | 2.771E-04 | 1.306 | 2.924E-04 |
| ENSG00000152229 | PSTPIP2 | mRNA | 1.055 | 2.904E-06 | 1.434 | 5.344E-05 |
| ENSG00000152253 | SPC25 | mRNA | 2.247 | 2.395E-06 | 1.492 | 8.525E-03 |
| ENSG00000152256 | PDK1 | mRNA | 1.054 | 1.962E-03 | 1.124 | 5.435E-03 |
| ENSG00000152315 | KCNK13 | mRNA | 1.388 | 1.955E-03 | 1.383 | 4.261E-03 |
| ENSG00000152467 | ZSCAN1 | mRNA | -3.082 | 8.060E-04 | -2.666 | 1.876E-03 |
| ENSG00000152495 | CAMK4 | mRNA | 1.923 | 7.701E-07 | 1.322 | 1.082E-03 |
| ENSG00000152578 | GRIA4 | mRNA | -1.251 | 1.223E-02 | -2.353 | 2.225E-05 |
| ENSG00000152592 | DMP1 | mRNA | -7.681 | 6.124E-06 | -5.017 | 2.732E-03 |
| ENSG00000152661 | GJA1 | mRNA | 1.306 | 1.899E-07 | 1.484 | 3.373E-06 |
| ENSG00000152766 | ANKRD22 | mRNA | 3.171 | 1.775E-14 | 3.059 | 2.040E-04 |
| ENSG00000152952 | PLOD2 | mRNA | 1.998 | 7.519E-14 | 1.833 | 1.940E-06 |
| ENSG00000152954 | NRSN1 | mRNA | -4.142 | 3.997E-05 | -4.020 | 8.436E-05 |
| ENSG00000153012 | LGI2 | mRNA | 1.003 | 1.245E-02 | 1.473 | 1.370E-04 |
| ENSG00000153157 | SYCP2L | mRNA | 1.176 | 6.102E-03 | 1.583 | 6.412E-04 |
| ENSG00000153250 | RBMS1 | mRNA | 1.325 | 2.017E-08 | 1.099 | 6.314E-04 |
| ENSG00000153292 | GPR110 | mRNA | 2.442 | 2.787E-12 | 1.287 | 4.874E-03 |
| ENSG00000153294 | GPR115 | mRNA | 3.228 | 6.049E-07 | 3.295 | 1.409E-06 |
| ENSG00000153303 | FRMD1 | mRNA | -4.667 | 2.149E-12 | -2.037 | 1.159E-03 |
| ENSG00000153310 | FAM49B | mRNA | 1.209 | 4.438E-10 | 1.067 | 7.935E-06 |
| ENSG00000153317 | ASAP1 | mRNA | 1.280 | 1.817E-08 | 1.306 | 3.570E-06 |
| ENSG00000153446 | C16orf89 | mRNA | -2.370 | 2.607E-06 | -2.463 | 6.037E-08 |
| ENSG00000153802 | TMPRSS11D | mRNA | 3.058 | 1.598E-17 | 1.515 | 2.399E-03 |
| ENSG00000153822 | KCNJ16 | mRNA | 3.023 | 1.178E-04 | 4.416 | 3.357E-11 |
| ENSG00000153902 | LGI4 | mRNA | -1.988 | 2.553E-12 | -1.387 | 2.732E-04 |
| ENSG00000154027 | AK5 | mRNA | 1.432 | 3.703E-06 | 1.729 | 1.386E-07 |
| ENSG00000154096 | THY1 | mRNA | 1.206 | 3.678E-06 | 1.532 | 2.346E-07 |
| ENSG00000154274 | C4orf19 | mRNA | -1.513 | 3.927E-11 | -1.963 | 6.298E-12 |
| ENSG00000154330 | PGM5 | mRNA | -1.584 | 5.112E-10 | -1.357 | 7.719E-06 |
| ENSG00000154451 | GBP5 | mRNA | 2.512 | 4.121E-06 | 1.791 | 5.492E-03 |
| ENSG00000154493 | C10orf90 | mRNA | -5.988 | 1.920E-19 | -5.692 | 1.216E-18 |
| ENSG00000154898 | CCDC144CP | mRNA | 1.857 | 8.393E-06 | 1.546 | 3.519E-03 |
| ENSG00000154914 | USP43 | mRNA | -1.503 | 1.621E-05 | -1.039 | 5.978E-03 |
| ENSG00000155093 | PTPRN2 | mRNA | -2.175 | 9.229E-14 | -1.445 | 4.322E-08 |
| ENSG00000155307 | SAMSN1 | mRNA | 2.689 | 1.724E-10 | 3.698 | 3.520E-09 |
| ENSG00000155380 | SLC16A1 | mRNA | 1.976 | 1.383E-17 | 1.863 | 1.916E-07 |
| ENSG00000155465 | SLC7A7 | mRNA | 1.586 | 3.002E-10 | 1.687 | 4.564E-09 |
| ENSG00000155657 | TTN | mRNA | 1.572 | 8.470E-07 | 1.500 | 7.269E-06 |
| ENSG00000155659 | VSIG4 | mRNA | 1.529 | 2.444E-07 | 3.355 | 1.553E-13 |
| ENSG00000155760 | FZD7 | mRNA | -1.793 | 4.975E-14 | -1.004 | 2.572E-03 |
| ENSG00000155792 | DEPTOR | mRNA | -3.146 | 3.553E-14 | -2.565 | 6.389E-09 |
| ENSG00000155897 | ADCY8 | mRNA | -6.690 | 7.967E-23 | -4.737 | 1.769E-07 |
| ENSG00000155918 | RAET1L | mRNA | 3.524 | 1.694E-07 | 3.462 | 1.593E-08 |
| ENSG00000155926 | SLA | mRNA | 1.472 | 4.328E-07 | 1.879 | 1.746E-10 |
| ENSG00000155961 | RAB39B | mRNA | 1.853 | 2.169E-07 | 1.342 | 1.276E-03 |
| ENSG00000156076 | WIF1 | mRNA | -4.010 | 1.535E-06 | -3.851 | 2.446E-07 |
| ENSG00000156113 | KCNMA1 | mRNA | -2.340 | 2.754E-17 | -1.682 | 3.308E-06 |
| ENSG00000156219 | ART3 | mRNA | -3.284 | 1.289E-07 | -2.996 | 2.327E-05 |
| ENSG00000156284 | CLDN8 | mRNA | -2.632 | 2.257E-12 | -2.043 | 2.831E-06 |
| ENSG00000156299 | TIAM1 | mRNA | 1.559 | 1.494E-10 | 1.424 | 4.119E-09 |
| ENSG00000156395 | SORCS3 | mRNA | -5.107 | 1.947E-10 | -3.286 | 7.644E-06 |
| ENSG00000156413 | FUT6 | mRNA | -2.107 | 5.124E-17 | -1.490 | 2.131E-06 |
| ENSG00000156486 | KCNS2 | mRNA | 1.179 | 2.293E-02 | 2.532 | 3.011E-07 |
| ENSG00000156535 | CD109 | mRNA | 2.072 | 1.942E-08 | 1.758 | 1.270E-05 |
| ENSG00000156687 | UNC5D | mRNA | -2.267 | 1.074E-03 | -2.529 | 6.537E-04 |
| ENSG00000156689 | GLYATL2 | mRNA | -1.840 | 1.235E-03 | -3.482 | 1.473E-07 |
| ENSG00000156755 | IGKV1OR-2 | mRNA | -3.113 | 2.777E-07 | -4.249 | 1.400E-10 |
| ENSG00000156966 | B3GNT7 | mRNA | -1.215 | 1.602E-05 | -1.234 | 1.498E-04 |
| ENSG00000156970 | BUB1B | mRNA | 2.845 | 1.105E-11 | 2.286 | 9.146E-06 |
| ENSG00000157103 | SLC6A1 | mRNA | 1.290 | 7.109E-04 | 1.961 | 2.671E-08 |
| ENSG00000157152 | SYN2 | mRNA | -1.613 | 6.329E-07 | -2.535 | 1.399E-12 |
| ENSG00000157343 | ARMC12 | mRNA | -2.195 | 6.291E-08 | -1.142 | 7.039E-03 |
| ENSG00000157456 | CCNB2 | mRNA | 3.186 | 1.379E-14 | 2.473 | 1.278E-05 |
| ENSG00000157551 | KCNJ15 | mRNA | 1.889 | 6.921E-10 | 1.086 | 6.820E-04 |
| ENSG00000157613 | CREB3L1 | mRNA | -1.753 | 6.186E-11 | -1.362 | 8.036E-07 |
| ENSG00000157766 | ACAN | mRNA | -1.562 | 4.047E-03 | -1.691 | 2.293E-03 |
| ENSG00000157992 | KRTCAP3 | mRNA | -1.904 | 5.273E-15 | -1.354 | 1.303E-05 |
| ENSG00000158055 | GRHL3 | mRNA | 1.151 | 9.559E-04 | 1.240 | 1.288E-03 |
| ENSG00000158106 | RHPN1 | mRNA | -1.789 | 2.325E-06 | -1.133 | 7.781E-03 |
| ENSG00000158125 | XDH | mRNA | -1.199 | 7.693E-06 | -1.979 | 9.409E-09 |
| ENSG00000158220 | ESYT3 | mRNA | -2.037 | 6.850E-11 | -1.635 | 5.014E-06 |
| ENSG00000158246 | FAM46B | mRNA | -2.551 | 1.169E-19 | -1.420 | 3.440E-08 |
| ENSG00000158258 | CLSTN2 | mRNA | -1.433 | 4.893E-06 | -1.238 | 1.008E-04 |
| ENSG00000158290 | CUL4B | mRNA | 1.284 | 8.842E-06 | 1.010 | 7.651E-03 |
| ENSG00000158402 | CDC25C | mRNA | 2.185 | 1.397E-06 | 1.580 | 3.164E-03 |
| ENSG00000158457 | TSPAN33 | mRNA | -1.118 | 9.754E-06 | -1.605 | 4.535E-10 |
| ENSG00000158458 | NRG2 | mRNA | -1.459 | 6.722E-10 | -1.236 | 1.100E-05 |
| ENSG00000158517 | NCF1 | mRNA | 1.950 | 9.280E-10 | 2.890 | 1.614E-13 |
| ENSG00000158528 | PPP1R9A | mRNA | -2.613 | 1.878E-15 | -2.713 | 1.175E-10 |
| ENSG00000158555 | GDPD5 | mRNA | -2.485 | 1.039E-28 | -1.686 | 4.467E-09 |
| ENSG00000158714 | SLAMF8 | mRNA | 2.617 | 9.279E-11 | 2.420 | 7.162E-09 |
| ENSG00000158825 | CDA | mRNA | 1.216 | 1.517E-03 | 1.819 | 3.393E-06 |
| ENSG00000158869 | FCER1G | mRNA | 3.546 | 2.401E-21 | 3.406 | 1.459E-10 |
| ENSG00000158887 | MPZ | mRNA | -2.512 | 8.869E-12 | -2.333 | 6.116E-10 |
| ENSG00000159123 | DMRTC1 | mRNA | -2.100 | 4.623E-05 | -1.830 | 1.391E-04 |
| ENSG00000159167 | STC1 | mRNA | 1.920 | 1.635E-08 | 2.899 | 4.937E-10 |
| ENSG00000159189 | C1QC | mRNA | 1.292 | 4.760E-05 | 2.251 | 3.287E-10 |
| ENSG00000159214 | CCDC24 | mRNA | -1.376 | 1.356E-04 | -1.009 | 3.549E-03 |
| ENSG00000159231 | CBR3 | mRNA | -2.675 | 5.024E-17 | -2.345 | 4.986E-09 |
| ENSG00000159339 | PADI4 | mRNA | 2.633 | 2.523E-04 | 4.057 | 2.594E-09 |
| ENSG00000159398 | CES5A | mRNA | -3.487 | 1.555E-06 | -3.105 | 3.925E-05 |
| ENSG00000159527 | PGLYRP3 | mRNA | 2.455 | 6.129E-04 | 3.793 | 1.187E-04 |
| ENSG00000159618 | GPR114 | mRNA | 1.074 | 5.614E-04 | 1.932 | 8.880E-07 |
| ENSG00000159763 | PIP | mRNA | -7.694 | 2.752E-17 | -6.565 | 3.671E-10 |
| ENSG00000160161 | CILP2 | mRNA | -4.113 | 1.370E-12 | -2.232 | 2.125E-04 |
| ENSG00000160181 | TFF2 | mRNA | -5.488 | 8.856E-10 | -3.932 | 3.030E-06 |
| ENSG00000160182 | TFF1 | mRNA | -3.286 | 1.326E-06 | -3.331 | 6.443E-06 |
| ENSG00000160200 | CBS | mRNA | -3.535 | 1.723E-35 | -2.529 | 1.091E-11 |
| ENSG00000160213 | CSTB | mRNA | 2.010 | 8.050E-18 | 1.387 | 1.938E-05 |
| ENSG00000160219 | GAB3 | mRNA | 1.154 | 8.566E-06 | 1.200 | 1.805E-05 |
| ENSG00000160255 | ITGB2 | mRNA | 1.048 | 1.043E-04 | 1.818 | 5.915E-09 |
| ENSG00000160307 | S100B | mRNA | -2.243 | 2.031E-06 | -2.311 | 7.609E-06 |
| ENSG00000160339 | FCN2 | mRNA | -4.069 | 4.291E-29 | -4.086 | 1.535E-17 |
| ENSG00000160401 | C9orf117 | mRNA | -2.050 | 6.575E-04 | -1.830 | 2.322E-03 |
| ENSG00000160593 | AMICA1 | mRNA | 1.525 | 6.346E-10 | 1.948 | 2.979E-10 |
| ENSG00000160654 | CD3G | mRNA | 1.817 | 1.967E-07 | 1.147 | 6.179E-03 |
| ENSG00000160678 | S100A1 | mRNA | -6.712 | 2.550E-70 | -4.437 | 6.606E-18 |
| ENSG00000160856 | FCRL3 | mRNA | 1.821 | 4.913E-05 | 1.245 | 7.170E-03 |
| ENSG00000160862 | AZGP1 | mRNA | -6.668 | 7.945E-65 | -5.107 | 2.439E-20 |
| ENSG00000160867 | FGFR4 | mRNA | -1.516 | 6.042E-06 | -1.118 | 4.258E-03 |
| ENSG00000160883 | HK3 | mRNA | 2.501 | 1.515E-08 | 4.598 | 5.048E-20 |
| ENSG00000160972 | PPP1R16A | mRNA | -1.737 | 2.188E-06 | -1.374 | 3.794E-04 |
| ENSG00000161055 | SCGB3A1 | mRNA | -6.644 | 7.753E-42 | -4.783 | 4.217E-11 |
| ENSG00000161082 | CELF5 | mRNA | -3.774 | 4.636E-14 | -3.640 | 1.177E-12 |
| ENSG00000161298 | ZNF382 | mRNA | 1.865 | 5.879E-08 | 1.311 | 1.921E-03 |
| ENSG00000161395 | PGAP3 | mRNA | -1.910 | 3.343E-20 | -1.124 | 2.306E-04 |
| ENSG00000161405 | IKZF3 | mRNA | 1.318 | 1.166E-06 | 1.098 | 1.469E-04 |
| ENSG00000161643 | SIGLEC16 | mRNA | 1.328 | 1.418E-03 | 1.453 | 6.732E-03 |
| ENSG00000161649 | CD300LG | mRNA | -4.801 | 1.015E-14 | -4.209 | 3.117E-11 |
| ENSG00000161798 | AQP5 | mRNA | -1.976 | 9.858E-17 | -2.407 | 1.868E-18 |
| ENSG00000161800 | RACGAP1 | mRNA | 1.748 | 3.994E-11 | 1.367 | 2.692E-05 |
| ENSG00000161911 | TREML1 | mRNA | 1.211 | 1.360E-03 | 1.988 | 5.371E-08 |
| ENSG00000161929 | SCIMP | mRNA | 1.179 | 2.703E-04 | 1.367 | 2.395E-04 |
| ENSG00000161944 | ASGR2 | mRNA | 1.422 | 1.486E-03 | 1.392 | 5.881E-03 |
| ENSG00000161958 | FGF11 | mRNA | 2.137 | 2.358E-13 | 2.179 | 1.942E-12 |
| ENSG00000162006 | MSLNL | mRNA | -2.570 | 2.220E-06 | -2.164 | 3.899E-04 |
| ENSG00000162065 | TBC1D24 | mRNA | -2.244 | 2.133E-31 | -1.730 | 4.655E-11 |
| ENSG00000162078 | ZG16B | mRNA | -6.719 | 2.284E-53 | -5.011 | 4.417E-11 |
| ENSG00000162105 | SHANK2 | mRNA | -2.634 | 5.389E-19 | -1.928 | 1.757E-12 |
| ENSG00000162337 | LRP5 | mRNA | -2.150 | 1.898E-30 | -1.412 | 1.921E-08 |
| ENSG00000162366 | PDZK1IP1 | mRNA | -1.131 | 1.048E-03 | -2.057 | 1.994E-08 |
| ENSG00000162437 | RAVER2 | mRNA | -1.062 | 1.442E-05 | -1.425 | 1.348E-06 |
| ENSG00000162511 | LAPTM5 | mRNA | 2.181 | 7.230E-18 | 2.086 | 2.511E-14 |
| ENSG00000162616 | DNAJB4 | mRNA | 1.371 | 3.378E-07 | 1.004 | 4.991E-03 |
| ENSG00000162618 | ELTD1 | mRNA | 1.744 | 6.092E-06 | 1.482 | 3.306E-03 |
| ENSG00000162692 | VCAM1 | mRNA | 1.002 | 4.224E-05 | 1.339 | 1.105E-06 |
| ENSG00000162704 | ARPC5 | mRNA | 1.571 | 4.475E-10 | 1.304 | 1.153E-04 |
| ENSG00000162706 | CADM3 | mRNA | -1.435 | 1.345E-02 | -3.206 | 2.235E-12 |
| ENSG00000162739 | SLAMF6 | mRNA | 2.243 | 2.796E-09 | 1.986 | 2.528E-06 |
| ENSG00000162747 | FCGR3B | mRNA | 4.461 | 5.995E-19 | 4.100 | 6.765E-10 |
| ENSG00000162782 | TDRD5 | mRNA | -2.659 | 7.103E-05 | -3.703 | 1.069E-07 |
| ENSG00000162817 | C1orf115 | mRNA | -1.681 | 1.882E-16 | -1.479 | 3.531E-09 |
| ENSG00000162873 | KLHDC8A | mRNA | -3.486 | 2.735E-18 | -3.541 | 1.656E-17 |
| ENSG00000162878 | PKDCC | mRNA | -3.627 | 1.244E-21 | -2.700 | 1.331E-08 |
| ENSG00000162896 | PIGR | mRNA | -3.265 | 5.357E-27 | -3.067 | 6.677E-25 |
| ENSG00000162951 | LRRTM1 | mRNA | -1.968 | 2.795E-06 | -2.502 | 3.694E-08 |
| ENSG00000162989 | KCNJ3 | mRNA | -2.596 | 5.454E-05 | -1.770 | 4.551E-03 |
| ENSG00000163009 | C2orf48 | mRNA | 2.842 | 6.336E-04 | 2.677 | 9.369E-04 |
| ENSG00000163017 | ACTG2 | mRNA | -1.920 | 7.314E-05 | -2.241 | 2.396E-07 |
| ENSG00000163046 | ANKRD30BL | mRNA | -2.610 | 1.095E-06 | -2.029 | 8.876E-04 |
| ENSG00000163083 | INHBB | mRNA | -1.964 | 1.717E-06 | -1.127 | 5.171E-03 |
| ENSG00000163106 | HPGDS | mRNA | 2.403 | 1.808E-13 | 2.765 | 1.313E-12 |
| ENSG00000163121 | NEURL3 | mRNA | -3.396 | 1.812E-23 | -3.496 | 7.683E-14 |
| ENSG00000163131 | CTSS | mRNA | 1.439 | 1.346E-09 | 1.136 | 3.483E-04 |
| ENSG00000163154 | TNFAIP8L2 | mRNA | 1.681 | 1.053E-05 | 1.867 | 2.268E-06 |
| ENSG00000163191 | S100A11 | mRNA | 1.574 | 6.410E-11 | 1.014 | 3.624E-03 |
| ENSG00000163207 | IVL | mRNA | 3.383 | 5.805E-03 | 6.496 | 2.764E-04 |
| ENSG00000163209 | SPRR3 | mRNA | 5.875 | 1.068E-12 | 5.580 | 2.989E-09 |
| ENSG00000163216 | SPRR2D | mRNA | 8.134 | 7.010E-12 | 7.613 | 2.492E-06 |
| ENSG00000163219 | ARHGAP25 | mRNA | 1.079 | 7.118E-06 | 1.316 | 4.663E-07 |
| ENSG00000163220 | S100A9 | mRNA | 4.269 | 8.542E-22 | 3.740 | 6.257E-13 |
| ENSG00000163221 | S100A12 | mRNA | 3.229 | 6.326E-07 | 2.960 | 8.617E-04 |
| ENSG00000163273 | NPPC | mRNA | -1.629 | 9.573E-05 | -1.907 | 3.706E-05 |
| ENSG00000163347 | CLDN1 | mRNA | 1.923 | 2.979E-16 | 1.672 | 1.019E-06 |
| ENSG00000163395 | IGFN1 | mRNA | -3.764 | 2.219E-08 | -2.627 | 3.403E-04 |
| ENSG00000163421 | PROK2 | mRNA | 4.286 | 4.236E-13 | 3.164 | 9.937E-07 |
| ENSG00000163449 | TMEM169 | mRNA | 1.901 | 1.459E-06 | 1.793 | 3.057E-05 |
| ENSG00000163464 | CXCR1 | mRNA | 3.707 | 5.721E-13 | 2.741 | 1.259E-06 |
| ENSG00000163466 | ARPC2 | mRNA | 1.410 | 2.187E-10 | 1.111 | 9.999E-05 |
| ENSG00000163485 | ADORA1 | mRNA | -1.629 | 9.871E-06 | -1.238 | 3.984E-03 |
| ENSG00000163492 | CCDC141 | mRNA | 1.799 | 3.681E-05 | 1.428 | 1.759E-03 |
| ENSG00000163508 | EOMES | mRNA | 1.836 | 6.176E-07 | 1.245 | 1.384E-03 |
| ENSG00000163519 | TRAT1 | mRNA | 3.117 | 3.623E-11 | 2.116 | 2.418E-04 |
| ENSG00000163535 | SGOL2 | mRNA | 1.893 | 8.989E-12 | 1.159 | 3.033E-03 |
| ENSG00000163563 | MNDA | mRNA | 3.540 | 2.010E-18 | 3.057 | 6.872E-07 |
| ENSG00000163565 | IFI16 | mRNA | 1.704 | 6.052E-12 | 1.154 | 1.103E-03 |
| ENSG00000163599 | CTLA4 | mRNA | 2.472 | 3.397E-09 | 1.668 | 3.150E-05 |
| ENSG00000163600 | ICOS | mRNA | 1.736 | 8.962E-07 | 1.332 | 2.641E-04 |
| ENSG00000163606 | CD200R1 | mRNA | 3.355 | 1.238E-15 | 3.075 | 4.986E-09 |
| ENSG00000163618 | CADPS | mRNA | -1.460 | 7.869E-05 | -1.174 | 5.452E-03 |
| ENSG00000163687 | DNASE1L3 | mRNA | -1.879 | 4.978E-08 | -1.996 | 6.144E-08 |
| ENSG00000163701 | IL17RE | mRNA | -1.822 | 9.565E-15 | -1.024 | 4.726E-04 |
| ENSG00000163702 | IL17RC | mRNA | -1.745 | 1.497E-17 | -1.073 | 1.197E-04 |
| ENSG00000163751 | CPA3 | mRNA | 3.057 | 1.625E-10 | 3.246 | 7.393E-09 |
| ENSG00000163808 | KIF15 | mRNA | 2.315 | 9.151E-10 | 1.641 | 1.438E-04 |
| ENSG00000163823 | CCR1 | mRNA | 2.637 | 2.160E-14 | 2.492 | 3.124E-10 |
| ENSG00000163864 | NMNAT3 | mRNA | -1.461 | 1.965E-09 | -1.220 | 3.734E-05 |
| ENSG00000163873 | GRIK3 | mRNA | -6.408 | 1.219E-27 | -4.873 | 1.825E-15 |
| ENSG00000163884 | KLF15 | mRNA | -3.784 | 1.130E-56 | -2.225 | 1.507E-10 |
| ENSG00000163975 | MFI2 | mRNA | -2.450 | 9.193E-27 | -1.315 | 1.178E-04 |
| ENSG00000164038 | SLC9B2 | mRNA | 1.093 | 1.975E-03 | 1.432 | 1.997E-05 |
| ENSG00000164106 | SCRG1 | mRNA | -3.265 | 2.153E-08 | -2.412 | 6.376E-05 |
| ENSG00000164109 | MAD2L1 | mRNA | 2.049 | 7.501E-12 | 1.287 | 4.747E-04 |
| ENSG00000164120 | HPGD | mRNA | 2.242 | 1.545E-07 | 2.503 | 2.378E-08 |
| ENSG00000164122 | ASB5 | mRNA | -2.411 | 3.212E-03 | -2.967 | 1.640E-03 |
| ENSG00000164128 | NPY1R | mRNA | -1.639 | 2.509E-04 | -1.918 | 5.090E-06 |
| ENSG00000164129 | NPY5R | mRNA | -1.723 | 1.626E-03 | -2.286 | 3.005E-05 |
| ENSG00000164176 | EDIL3 | mRNA | 1.751 | 1.765E-15 | 2.113 | 2.948E-11 |
| ENSG00000164211 | STARD4 | mRNA | 1.443 | 6.160E-05 | 1.514 | 2.767E-03 |
| ENSG00000164400 | CSF2 | mRNA | 3.045 | 7.117E-03 | 5.459 | 2.098E-06 |
| ENSG00000164451 | FAM26D | mRNA | 2.675 | 2.081E-02 | 3.069 | 5.017E-03 |
| ENSG00000164483 | SAMD3 | mRNA | 2.324 | 9.419E-08 | 2.185 | 8.537E-06 |
| ENSG00000164484 | TMEM200A | mRNA | 1.112 | 6.545E-06 | 1.838 | 8.616E-11 |
| ENSG00000164488 | DACT2 | mRNA | -2.580 | 6.704E-21 | -1.121 | 3.146E-04 |
| ENSG00000164509 | IL31RA | mRNA | 2.779 | 1.687E-04 | 2.356 | 6.498E-03 |
| ENSG00000164512 | ANKRD55 | mRNA | 1.232 | 1.526E-02 | 1.614 | 1.939E-03 |
| ENSG00000164530 | PI16 | mRNA | -4.662 | 1.542E-22 | -3.528 | 3.756E-06 |
| ENSG00000164611 | PTTG1 | mRNA | 1.815 | 1.606E-08 | 1.671 | 4.359E-06 |
| ENSG00000164659 | KIAA1324L | mRNA | -1.166 | 2.902E-05 | -1.460 | 9.374E-06 |
| ENSG00000164687 | FABP5 | mRNA | 2.993 | 4.543E-18 | 2.899 | 3.558E-07 |
| ENSG00000164691 | TAGAP | mRNA | 1.493 | 1.361E-07 | 1.634 | 1.499E-06 |
| ENSG00000164761 | TNFRSF11B | mRNA | 1.340 | 1.274E-04 | 1.869 | 2.849E-05 |
| ENSG00000164932 | CTHRC1 | mRNA | 1.180 | 2.773E-04 | 1.329 | 3.952E-04 |
| ENSG00000165025 | SYK | mRNA | 1.027 | 6.154E-08 | 1.206 | 3.935E-09 |
| ENSG00000165078 | CPA6 | mRNA | 1.093 | 1.721E-02 | 1.527 | 9.211E-04 |
| ENSG00000165140 | FBP1 | mRNA | -1.735 | 4.057E-12 | -1.420 | 1.248E-07 |
| ENSG00000165168 | CYBB | mRNA | 2.984 | 1.177E-20 | 2.794 | 1.194E-09 |
| ENSG00000165178 | NCF1C | mRNA | 1.999 | 8.034E-09 | 2.919 | 3.374E-12 |
| ENSG00000165186 | PTCHD1 | mRNA | -2.774 | 6.568E-07 | -2.343 | 2.533E-05 |
| ENSG00000165215 | CLDN3 | mRNA | -2.237 | 8.633E-15 | -1.669 | 4.629E-08 |
| ENSG00000165238 | WNK2 | mRNA | -2.833 | 2.468E-30 | -2.096 | 7.989E-12 |
| ENSG00000165269 | AQP7 | mRNA | -1.727 | 3.071E-06 | -1.072 | 3.137E-03 |
| ENSG00000165300 | SLITRK5 | mRNA | -4.371 | 9.922E-30 | -4.356 | 3.880E-21 |
| ENSG00000165304 | MELK | mRNA | 3.643 | 6.752E-17 | 2.767 | 1.100E-06 |
| ENSG00000165323 | FAT3 | mRNA | -1.967 | 1.497E-05 | -2.010 | 2.767E-05 |
| ENSG00000165349 | SLC7A3 | mRNA | -1.708 | 2.298E-05 | -2.018 | 6.180E-06 |
| ENSG00000165376 | CLDN2 | mRNA | -5.866 | 9.300E-10 | -4.560 | 7.829E-07 |
| ENSG00000165388 | ZNF488 | mRNA | 1.429 | 1.094E-04 | 1.232 | 3.420E-04 |
| ENSG00000165390 | ANXA8 | mRNA | 1.831 | 1.462E-06 | 2.039 | 7.703E-08 |
| ENSG00000165457 | FOLR2 | mRNA | 1.255 | 1.414E-05 | 2.593 | 3.411E-13 |
| ENSG00000165474 | GJB2 | mRNA | 1.454 | 6.542E-08 | 1.248 | 7.866E-05 |
| ENSG00000165475 | CRYL1 | mRNA | -1.343 | 1.237E-10 | -1.146 | 4.584E-06 |
| ENSG00000165478 | HEPACAM | mRNA | -2.338 | 2.301E-05 | -3.928 | 8.642E-09 |
| ENSG00000165480 | SKA3 | mRNA | 3.266 | 5.864E-12 | 2.317 | 2.737E-04 |
| ENSG00000165568 | AKR1E2 | mRNA | -1.630 | 5.751E-07 | -1.128 | 1.275E-03 |
| ENSG00000165588 | OTX2 | mRNA | 2.439 | 1.855E-10 | 2.017 | 6.729E-05 |
| ENSG00000165621 | OXGR1 | mRNA | -2.374 | 4.348E-06 | -3.333 | 5.663E-07 |
| ENSG00000165626 | BEND7 | mRNA | -1.213 | 1.741E-08 | -1.584 | 1.528E-09 |
| ENSG00000165685 | TMEM52B | mRNA | 3.115 | 5.807E-06 | 3.141 | 7.559E-05 |
| ENSG00000165702 | GFI1B | mRNA | 3.029 | 6.940E-05 | 4.517 | 2.531E-12 |
| ENSG00000165795 | NDRG2 | mRNA | -2.886 | 1.136E-30 | -2.506 | 6.457E-16 |
| ENSG00000165816 | VWA2 | mRNA | -1.556 | 1.279E-08 | -1.658 | 1.947E-08 |
| ENSG00000165828 | PRAP1 | mRNA | -2.773 | 1.366E-03 | -4.231 | 6.436E-05 |
| ENSG00000165863 | C10orf82 | mRNA | -2.346 | 5.303E-07 | -3.176 | 1.105E-09 |
| ENSG00000165891 | E2F7 | mRNA | 2.228 | 9.723E-11 | 1.241 | 1.240E-03 |
| ENSG00000165905 | GYLTL1B | mRNA | -1.932 | 2.728E-16 | -1.533 | 3.288E-07 |
| ENSG00000165966 | PDZRN4 | mRNA | -3.513 | 3.123E-09 | -4.251 | 8.336E-12 |
| ENSG00000165973 | NELL1 | mRNA | 3.187 | 2.210E-04 | 2.442 | 4.233E-04 |
| ENSG00000165985 | C1QL3 | mRNA | -1.459 | 1.405E-02 | -1.932 | 3.822E-03 |
| ENSG00000166016 | ABTB2 | mRNA | -2.164 | 6.682E-20 | -1.120 | 5.756E-04 |
| ENSG00000166091 | CMTM5 | mRNA | -3.383 | 6.891E-10 | -2.576 | 4.654E-06 |
| ENSG00000166123 | GPT2 | mRNA | -2.042 | 2.220E-22 | -1.556 | 7.530E-08 |
| ENSG00000166130 | IKBIP | mRNA | 1.226 | 4.636E-07 | 1.056 | 9.302E-04 |
| ENSG00000166183 | ASPG | mRNA | -2.106 | 7.022E-15 | -1.679 | 2.920E-07 |
| ENSG00000166342 | NETO1 | mRNA | 3.177 | 1.573E-08 | 3.434 | 2.198E-08 |
| ENSG00000166426 | CRABP1 | mRNA | 2.055 | 5.900E-05 | 2.757 | 2.332E-07 |
| ENSG00000166501 | PRKCB | mRNA | 1.671 | 2.871E-10 | 1.765 | 1.494E-08 |
| ENSG00000166523 | CLEC4E | mRNA | 2.701 | 1.706E-05 | 2.283 | 2.252E-03 |
| ENSG00000166527 | CLEC4D | mRNA | 4.386 | 1.530E-11 | 3.057 | 2.430E-03 |
| ENSG00000166535 | A2ML1 | mRNA | 2.408 | 2.252E-04 | 2.706 | 7.550E-05 |
| ENSG00000166557 | TMED3 | mRNA | -2.380 | 1.374E-24 | -2.186 | 1.615E-16 |
| ENSG00000166582 | CENPV | mRNA | -2.141 | 2.358E-13 | -1.332 | 1.495E-04 |
| ENSG00000166670 | MMP10 | mRNA | 2.680 | 7.322E-11 | 2.165 | 7.037E-05 |
| ENSG00000166682 | TMPRSS5 | mRNA | -5.205 | 3.772E-22 | -2.077 | 3.851E-03 |
| ENSG00000166736 | HTR3A | mRNA | 3.134 | 1.890E-05 | 2.623 | 1.833E-03 |
| ENSG00000166803 | KIAA0101 | mRNA | 2.743 | 2.266E-10 | 1.908 | 5.215E-05 |
| ENSG00000166816 | LDHD | mRNA | -3.162 | 2.130E-31 | -2.252 | 8.025E-12 |
| ENSG00000166823 | MESP1 | mRNA | -2.104 | 7.085E-10 | -1.295 | 6.515E-03 |
| ENSG00000166840 | GLYATL1 | mRNA | -2.052 | 2.098E-05 | -3.199 | 2.466E-08 |
| ENSG00000166897 | ELFN2 | mRNA | 2.216 | 1.372E-03 | 1.764 | 7.397E-03 |
| ENSG00000166920 | C15orf48 | mRNA | 4.145 | 4.429E-16 | 2.164 | 4.497E-04 |
| ENSG00000166922 | SCG5 | mRNA | 2.147 | 1.597E-03 | 3.444 | 1.137E-09 |
| ENSG00000166923 | GREM1 | mRNA | 2.822 | 1.874E-16 | 2.732 | 9.916E-09 |
| ENSG00000166927 | MS4A7 | mRNA | 2.399 | 3.567E-12 | 2.344 | 1.138E-10 |
| ENSG00000167034 | NKX3-1 | mRNA | -3.118 | 2.653E-22 | -4.256 | 1.757E-35 |
| ENSG00000167080 | B4GALNT2 | mRNA | 3.150 | 1.826E-06 | 2.589 | 6.614E-04 |
| ENSG00000167085 | PHB | mRNA | -1.359 | 7.182E-13 | -1.247 | 7.332E-09 |
| ENSG00000167165 | UGT1A6 | mRNA | 1.129 | 9.198E-04 | 1.435 | 3.843E-04 |
| ENSG00000167178 | ISLR2 | mRNA | -2.506 | 2.676E-11 | -1.494 | 9.094E-04 |
| ENSG00000167183 | PRR15L | mRNA | -1.725 | 3.610E-11 | -1.630 | 9.762E-08 |
| ENSG00000167208 | SNX20 | mRNA | 1.712 | 1.033E-07 | 2.115 | 2.182E-08 |
| ENSG00000167236 | CCL23 | mRNA | 1.883 | 2.443E-03 | 3.732 | 2.802E-14 |
| ENSG00000167311 | ART5 | mRNA | -2.737 | 7.371E-08 | -1.287 | 8.384E-03 |
| ENSG00000167315 | ACAA2 | mRNA | 1.360 | 8.868E-06 | 1.571 | 8.886E-06 |
| ENSG00000167363 | FN3K | mRNA | -2.598 | 1.850E-22 | -1.723 | 9.982E-08 |
| ENSG00000167419 | LPO | mRNA | -8.841 | 1.800E-101 | -6.190 | 2.387E-21 |
| ENSG00000167588 | GPD1 | mRNA | -2.613 | 1.905E-20 | -3.024 | 8.543E-21 |
| ENSG00000167608 | TMC4 | mRNA | -1.206 | 9.501E-07 | -1.018 | 1.332E-04 |
| ENSG00000167614 | TTYH1 | mRNA | -6.211 | 2.279E-43 | -3.737 | 2.948E-10 |
| ENSG00000167618 | LAIR2 | mRNA | 3.045 | 2.486E-07 | 2.851 | 8.827E-05 |
| ENSG00000167619 | TMEM145 | mRNA | -1.932 | 1.887E-05 | -1.732 | 7.804E-05 |
| ENSG00000167642 | SPINT2 | mRNA | -1.401 | 3.426E-08 | -1.076 | 1.417E-04 |
| ENSG00000167656 | LY6D | mRNA | 3.312 | 1.095E-07 | 3.055 | 1.797E-05 |
| ENSG00000167676 | PLIN4 | mRNA | -1.894 | 5.051E-13 | -1.841 | 1.176E-09 |
| ENSG00000167700 | MFSD3 | mRNA | -2.027 | 1.709E-13 | -1.102 | 3.774E-03 |
| ENSG00000167701 | GPT | mRNA | -3.085 | 1.004E-15 | -3.275 | 1.323E-14 |
| ENSG00000167733 | HSD11B1L | mRNA | -1.731 | 3.491E-07 | -1.101 | 1.313E-03 |
| ENSG00000167741 | GGT6 | mRNA | -1.547 | 2.313E-14 | -1.585 | 7.813E-09 |
| ENSG00000167757 | KLK11 | mRNA | -2.714 | 3.106E-13 | -1.704 | 1.844E-07 |
| ENSG00000167850 | CD300C | mRNA | 1.461 | 1.693E-04 | 1.839 | 1.006E-06 |
| ENSG00000167851 | CD300A | mRNA | 1.175 | 5.613E-05 | 1.556 | 1.369E-06 |
| ENSG00000167861 | HID1 | mRNA | -2.576 | 3.690E-28 | -1.673 | 5.006E-08 |
| ENSG00000167914 | GSDMA | mRNA | 1.698 | 1.470E-03 | 3.082 | 2.557E-07 |
| ENSG00000167916 | KRT24 | mRNA | 5.511 | 7.821E-07 | 6.099 | 2.263E-06 |
| ENSG00000167964 | RAB26 | mRNA | -2.345 | 1.493E-18 | -1.673 | 3.666E-07 |
| ENSG00000167971 | CASKIN1 | mRNA | -2.835 | 4.427E-20 | -1.805 | 1.016E-05 |
| ENSG00000167972 | ABCA3 | mRNA | -2.910 | 6.178E-39 | -2.103 | 9.612E-13 |
| ENSG00000168077 | SCARA3 | mRNA | -1.311 | 7.869E-12 | -1.077 | 1.604E-04 |
| ENSG00000168078 | PBK | mRNA | 3.549 | 3.820E-11 | 2.708 | 1.051E-04 |
| ENSG00000168242 | HIST1H2BI | mRNA | 2.692 | 1.307E-11 | 1.919 | 6.520E-05 |
| ENSG00000168274 | HIST1H2AE | mRNA | 2.257 | 1.367E-12 | 1.443 | 7.547E-04 |
| ENSG00000168280 | KIF5C | mRNA | 1.606 | 2.709E-05 | 1.436 | 2.280E-04 |
| ENSG00000168309 | FAM107A | mRNA | -1.813 | 4.980E-10 | -1.047 | 4.555E-03 |
| ENSG00000168333 | C8orf22 | mRNA | 1.589 | 1.931E-02 | -2.655 | 6.936E-04 |
| ENSG00000168350 | DEGS2 | mRNA | -1.596 | 2.482E-07 | -1.033 | 5.405E-03 |
| ENSG00000168453 | HR | mRNA | -1.548 | 1.524E-07 | -1.117 | 7.212E-04 |
| ENSG00000168461 | RAB31 | mRNA | 1.841 | 1.078E-21 | 1.608 | 1.235E-08 |
| ENSG00000168539 | CHRM1 | mRNA | -6.304 | 1.807E-46 | -4.819 | 2.409E-24 |
| ENSG00000168621 | GDNF | mRNA | 1.167 | 1.320E-02 | 2.084 | 4.120E-07 |
| ENSG00000168672 | FAM84B | mRNA | -1.470 | 1.260E-16 | -1.376 | 3.338E-11 |
| ENSG00000168685 | IL7R | mRNA | 1.789 | 1.534E-07 | 1.541 | 2.100E-05 |
| ENSG00000168995 | SIGLEC7 | mRNA | 2.607 | 8.536E-10 | 3.211 | 4.674E-14 |
| ENSG00000169035 | KLK7 | mRNA | 2.166 | 4.445E-03 | 2.845 | 8.277E-05 |
| ENSG00000169083 | AR | mRNA | -1.448 | 7.709E-10 | -1.775 | 1.605E-07 |
| ENSG00000169194 | IL13 | mRNA | 3.097 | 7.443E-04 | 5.409 | 2.444E-10 |
| ENSG00000169224 | GCSAML | mRNA | 3.041 | 3.379E-10 | 3.210 | 9.503E-09 |
| ENSG00000169245 | CXCL10 | mRNA | 2.760 | 1.409E-04 | 4.691 | 2.835E-04 |
| ENSG00000169248 | CXCL11 | mRNA | 2.483 | 3.066E-03 | 3.712 | 5.025E-03 |
| ENSG00000169282 | KCNAB1 | mRNA | -1.221 | 7.143E-04 | -1.802 | 2.718E-05 |
| ENSG00000169347 | GP2 | mRNA | -4.855 | 1.499E-41 | -3.614 | 1.349E-17 |
| ENSG00000169385 | RNASE2 | mRNA | 4.394 | 1.730E-12 | 5.565 | 1.834E-06 |
| ENSG00000169397 | RNASE3 | mRNA | 2.954 | 5.613E-04 | 3.224 | 4.241E-03 |
| ENSG00000169403 | PTAFR | mRNA | 1.873 | 1.059E-13 | 2.241 | 5.543E-16 |
| ENSG00000169442 | CD52 | mRNA | 2.524 | 1.143E-09 | 2.333 | 3.600E-05 |
| ENSG00000169469 | SPRR1B | mRNA | 5.719 | 1.444E-09 | 6.470 | 8.589E-10 |
| ENSG00000169474 | SPRR1A | mRNA | 5.945 | 1.342E-11 | 7.058 | 3.062E-09 |
| ENSG00000169507 | SLC38A11 | mRNA | 1.623 | 2.934E-05 | 1.273 | 1.476E-03 |
| ENSG00000169508 | GPR183 | mRNA | 1.905 | 1.534E-05 | 1.564 | 1.811E-03 |
| ENSG00000169562 | GJB1 | mRNA | -2.402 | 6.471E-12 | -1.857 | 4.267E-09 |
| ENSG00000169594 | BNC1 | mRNA | 1.812 | 1.881E-06 | 2.037 | 1.923E-05 |
| ENSG00000169604 | ANTXR1 | mRNA | 1.150 | 5.275E-09 | 1.346 | 2.049E-06 |
| ENSG00000169607 | CKAP2L | mRNA | 3.703 | 4.003E-15 | 2.804 | 3.733E-05 |
| ENSG00000169679 | BUB1 | mRNA | 1.600 | 1.271E-05 | 1.298 | 1.116E-03 |
| ENSG00000169684 | CHRNA5 | mRNA | 1.914 | 4.375E-08 | 1.207 | 3.837E-03 |
| ENSG00000169704 | GP9 | mRNA | -1.756 | 2.370E-03 | -2.286 | 2.301E-03 |
| ENSG00000169738 | DCXR | mRNA | -2.214 | 4.646E-22 | -1.266 | 2.609E-05 |
| ENSG00000169750 | RAC3 | mRNA | -2.087 | 2.156E-12 | -1.125 | 1.089E-03 |
| ENSG00000169783 | LINGO1 | mRNA | -1.553 | 3.333E-06 | -1.180 | 1.274E-03 |
| ENSG00000169826 | CSGALNACT2 | mRNA | 1.261 | 1.340E-07 | 1.215 | 5.016E-05 |
| ENSG00000169855 | ROBO1 | mRNA | 1.031 | 6.257E-07 | 1.368 | 1.059E-06 |
| ENSG00000169896 | ITGAM | mRNA | 1.936 | 3.540E-10 | 3.343 | 6.964E-26 |
| ENSG00000169946 | ZFPM2 | mRNA | -1.185 | 2.675E-05 | -1.602 | 5.575E-07 |
| ENSG00000170006 | TMEM154 | mRNA | 1.611 | 1.137E-12 | 1.575 | 1.175E-06 |
| ENSG00000170290 | SLN | mRNA | 4.691 | 2.691E-06 | 5.546 | 7.590E-09 |
| ENSG00000170312 | CDK1 | mRNA | 3.125 | 3.174E-17 | 2.107 | 1.095E-04 |
| ENSG00000170323 | FABP4 | mRNA | -2.142 | 5.627E-05 | -2.135 | 1.543E-05 |
| ENSG00000170324 | FRMPD2 | mRNA | -1.198 | 7.407E-03 | -1.326 | 3.353E-03 |
| ENSG00000170374 | SP7 | mRNA | -5.890 | 2.316E-11 | -3.999 | 5.540E-06 |
| ENSG00000170381 | SEMA3E | mRNA | -2.212 | 1.182E-06 | -3.318 | 1.674E-15 |
| ENSG00000170382 | LRRN2 | mRNA | -3.848 | 1.322E-17 | -2.546 | 1.364E-08 |
| ENSG00000170396 | ZNF804A | mRNA | 1.486 | 1.775E-04 | 1.158 | 6.694E-03 |
| ENSG00000170454 | KRT75 | mRNA | 2.550 | 7.458E-03 | 2.772 | 5.040E-03 |
| ENSG00000170549 | IRX1 | mRNA | -3.664 | 2.153E-22 | -2.790 | 7.565E-11 |
| ENSG00000170561 | IRX2 | mRNA | -2.915 | 6.087E-18 | -2.494 | 4.620E-10 |
| ENSG00000170571 | EMB | mRNA | 1.846 | 1.491E-14 | 1.239 | 2.744E-04 |
| ENSG00000170579 | DLGAP1 | mRNA | -1.203 | 1.386E-03 | -1.733 | 5.149E-05 |
| ENSG00000170775 | GPR37 | mRNA | -1.514 | 8.537E-06 | -2.029 | 9.654E-08 |
| ENSG00000170858 | LILRP2 | mRNA | 2.397 | 1.798E-04 | 2.139 | 3.925E-03 |
| ENSG00000170891 | CYTL1 | mRNA | 1.668 | 5.502E-04 | 2.018 | 6.113E-05 |
| ENSG00000170909 | OSCAR | mRNA | 1.421 | 7.129E-04 | 2.816 | 7.006E-10 |
| ENSG00000170956 | CEACAM3 | mRNA | 1.888 | 5.518E-08 | 1.265 | 5.240E-04 |
| ENSG00000171017 | LRRC8E | mRNA | -1.358 | 6.076E-07 | -1.037 | 1.584E-03 |
| ENSG00000171049 | FPR2 | mRNA | 1.917 | 1.240E-04 | 1.954 | 9.789E-04 |
| ENSG00000171051 | FPR1 | mRNA | 3.472 | 9.553E-20 | 3.361 | 8.587E-10 |
| ENSG00000171101 | SIGLEC17P | mRNA | 1.521 | 1.378E-03 | 1.740 | 1.142E-04 |
| ENSG00000171119 | NRTN | mRNA | -2.897 | 3.346E-23 | -1.565 | 6.982E-05 |
| ENSG00000171130 | ATP6V0E2 | mRNA | -2.613 | 5.099E-30 | -2.053 | 3.395E-13 |
| ENSG00000171195 | MUC7 | mRNA | -7.849 | 1.036E-38 | -4.152 | 5.742E-06 |
| ENSG00000171201 | SMR3B | mRNA | -4.590 | 6.272E-15 | -2.187 | 8.548E-03 |
| ENSG00000171224 | C10orf35 | mRNA | -1.008 | 4.407E-04 | -1.249 | 4.904E-05 |
| ENSG00000171234 | UGT2B7 | mRNA | -1.764 | 5.241E-04 | -1.581 | 1.681E-03 |
| ENSG00000171243 | SOSTDC1 | mRNA | -1.866 | 1.399E-07 | -2.343 | 1.186E-07 |
| ENSG00000171302 | CANT1 | mRNA | -1.600 | 3.312E-12 | -1.089 | 3.105E-06 |
| ENSG00000171310 | CHST11 | mRNA | 1.029 | 1.307E-05 | 1.337 | 2.004E-07 |
| ENSG00000171320 | ESCO2 | mRNA | 1.588 | 8.854E-06 | 1.121 | 1.846E-03 |
| ENSG00000171401 | KRT13 | mRNA | 4.146 | 1.018E-08 | 4.804 | 9.238E-08 |
| ENSG00000171476 | HOPX | mRNA | 1.182 | 6.129E-05 | 1.265 | 5.860E-04 |
| ENSG00000171488 | LRRC8C | mRNA | 1.572 | 6.150E-07 | 1.197 | 2.522E-03 |
| ENSG00000171631 | P2RY6 | mRNA | 1.460 | 1.646E-07 | 1.010 | 3.437E-04 |
| ENSG00000171657 | GPR82 | mRNA | 2.361 | 9.824E-09 | 2.097 | 4.183E-04 |
| ENSG00000171658 | RP11-443P15.2 | mRNA | 1.116 | 1.410E-02 | 1.595 | 2.785E-03 |
| ENSG00000171659 | GPR34 | mRNA | 2.993 | 8.824E-09 | 2.494 | 2.340E-04 |
| ENSG00000171714 | ANO5 | mRNA | -3.628 | 4.570E-17 | -3.209 | 1.004E-10 |
| ENSG00000171766 | GATM | mRNA | -2.363 | 1.962E-11 | -1.620 | 5.681E-05 |
| ENSG00000171819 | ANGPTL7 | mRNA | -4.170 | 1.676E-08 | -6.351 | 1.059E-15 |
| ENSG00000171848 | RRM2 | mRNA | 2.914 | 5.340E-11 | 2.324 | 3.880E-07 |
| ENSG00000171860 | C3AR1 | mRNA | 2.413 | 7.605E-15 | 2.794 | 1.349E-14 |
| ENSG00000171864 | PRND | mRNA | 2.654 | 3.716E-04 | 2.935 | 3.856E-04 |
| ENSG00000171951 | SCG2 | mRNA | 4.693 | 8.449E-38 | 3.553 | 1.482E-13 |
| ENSG00000172156 | CCL11 | mRNA | 4.416 | 4.033E-16 | 3.329 | 3.621E-06 |
| ENSG00000172201 | ID4 | mRNA | -2.531 | 8.339E-26 | -2.119 | 1.042E-10 |
| ENSG00000172243 | CLEC7A | mRNA | 2.322 | 2.385E-14 | 1.857 | 8.749E-07 |
| ENSG00000172322 | CLEC12A | mRNA | 3.114 | 3.136E-14 | 3.526 | 4.556E-10 |
| ENSG00000172458 | IL17D | mRNA | -1.982 | 6.186E-07 | -1.459 | 3.383E-04 |
| ENSG00000172548 | NIPAL4 | mRNA | 1.980 | 1.281E-04 | 2.052 | 2.133E-03 |
| ENSG00000172554 | SNTG2 | mRNA | 1.009 | 4.655E-04 | 1.122 | 8.351E-04 |
| ENSG00000172575 | RASGRP1 | mRNA | 2.113 | 1.412E-12 | 1.396 | 2.405E-05 |
| ENSG00000172602 | RND1 | mRNA | -1.536 | 3.607E-03 | -1.219 | 5.512E-03 |
| ENSG00000172752 | COL6A5 | mRNA | 5.418 | 3.179E-06 | 6.535 | 3.882E-12 |
| ENSG00000172824 | CES4A | mRNA | -1.439 | 7.209E-05 | -1.043 | 4.488E-03 |
| ENSG00000172828 | CES3 | mRNA | -2.817 | 1.516E-26 | -2.401 | 6.528E-14 |
| ENSG00000173198 | CYSLTR1 | mRNA | 1.504 | 1.394E-09 | 1.263 | 2.745E-04 |
| ENSG00000173369 | C1QB | mRNA | 1.858 | 3.565E-09 | 2.676 | 1.990E-13 |
| ENSG00000173372 | C1QA | mRNA | 1.398 | 5.969E-06 | 2.085 | 5.542E-11 |
| ENSG00000173391 | OLR1 | mRNA | 2.069 | 1.582E-04 | 3.197 | 5.831E-07 |
| ENSG00000173535 | TNFRSF10C | mRNA | 2.017 | 1.540E-12 | 1.809 | 1.522E-08 |
| ENSG00000173559 | NABP1 | mRNA | 1.829 | 8.467E-13 | 1.570 | 3.048E-07 |
| ENSG00000173578 | XCR1 | mRNA | 2.294 | 8.387E-08 | 1.656 | 8.972E-05 |
| ENSG00000173599 | PC | mRNA | -1.837 | 2.422E-15 | -1.330 | 2.310E-07 |
| ENSG00000173626 | TRAPPC3L | mRNA | 4.055 | 7.328E-18 | 3.895 | 2.104E-08 |
| ENSG00000173698 | GPR64 | mRNA | -1.771 | 4.215E-12 | -1.380 | 2.870E-06 |
| ENSG00000173714 | WFIKKN2 | mRNA | -2.965 | 9.809E-12 | -1.418 | 2.708E-03 |
| ENSG00000173852 | DPY19L1 | mRNA | 1.688 | 6.638E-13 | 1.087 | 5.983E-04 |
| ENSG00000174080 | CTSF | mRNA | -2.380 | 6.051E-27 | -1.212 | 1.041E-04 |
| ENSG00000174083 | PIK3R6 | mRNA | 1.529 | 3.903E-06 | 2.646 | 2.321E-14 |
| ENSG00000174136 | RGMB | mRNA | -1.142 | 1.035E-10 | -1.150 | 7.378E-08 |
| ENSG00000174276 | ZNHIT2 | mRNA | -1.543 | 3.718E-05 | -1.069 | 6.723E-03 |
| ENSG00000174371 | EXO1 | mRNA | 2.755 | 2.451E-09 | 1.998 | 8.089E-04 |
| ENSG00000174469 | CNTNAP2 | mRNA | 1.588 | 3.536E-04 | 2.380 | 1.609E-05 |
| ENSG00000174485 | DENND4A | mRNA | 1.147 | 2.269E-05 | 1.132 | 1.917E-03 |
| ENSG00000174564 | IL20RB | mRNA | 1.254 | 7.392E-04 | 1.070 | 2.308E-03 |
| ENSG00000174567 | GOLT1A | mRNA | -1.436 | 1.842E-05 | -1.863 | 9.223E-08 |
| ENSG00000174600 | CMKLR1 | mRNA | 1.108 | 2.524E-04 | 1.471 | 5.250E-07 |
| ENSG00000174607 | UGT8 | mRNA | -1.166 | 2.045E-03 | -2.008 | 6.996E-06 |
| ENSG00000174611 | KY | mRNA | -2.448 | 9.718E-11 | -2.059 | 3.416E-08 |
| ENSG00000174721 | FGFBP3 | mRNA | -2.061 | 1.039E-08 | -1.693 | 1.638E-04 |
| ENSG00000174799 | CEP135 | mRNA | 1.601 | 4.087E-11 | 1.033 | 1.351E-03 |
| ENSG00000174837 | EMR1 | mRNA | 4.634 | 8.000E-14 | 5.675 | 1.442E-21 |
| ENSG00000174899 | C3orf55 | mRNA | -1.612 | 1.088E-02 | -1.731 | 8.186E-03 |
| ENSG00000174938 | SEZ6L2 | mRNA | -1.679 | 1.713E-15 | -1.577 | 3.763E-11 |
| ENSG00000174945 | AMZ1 | mRNA | 1.777 | 4.880E-04 | 2.822 | 2.244E-06 |
| ENSG00000174946 | GPR171 | mRNA | 2.312 | 5.960E-07 | 1.772 | 1.054E-03 |
| ENSG00000175063 | UBE2C | mRNA | 2.738 | 3.025E-10 | 2.344 | 3.197E-06 |
| ENSG00000175084 | DES | mRNA | -4.625 | 9.739E-21 | -4.846 | 5.684E-15 |
| ENSG00000175121 | WFDC5 | mRNA | 2.325 | 4.120E-04 | 2.528 | 9.424E-05 |
| ENSG00000175161 | CADM2 | mRNA | -3.024 | 6.927E-05 | -4.161 | 7.251E-08 |
| ENSG00000175264 | CHST1 | mRNA | -2.962 | 2.995E-17 | -1.522 | 3.900E-05 |
| ENSG00000175294 | CATSPER1 | mRNA | 2.413 | 3.973E-04 | 1.839 | 3.571E-03 |
| ENSG00000175471 | MCTP1 | mRNA | 1.298 | 6.557E-08 | 1.138 | 7.288E-04 |
| ENSG00000175489 | LRRC25 | mRNA | 1.453 | 1.450E-05 | 2.240 | 2.272E-10 |
| ENSG00000175556 | LONRF3 | mRNA | -2.401 | 1.133E-32 | -2.158 | 3.226E-18 |
| ENSG00000175785 | PRIMA1 | mRNA | -2.024 | 1.485E-08 | -1.765 | 2.067E-05 |
| ENSG00000175793 | SFN | mRNA | 1.061 | 6.012E-03 | 1.238 | 1.265E-03 |
| ENSG00000175857 | GAPT | mRNA | 4.084 | 5.430E-12 | 4.440 | 3.566E-08 |
| ENSG00000175868 | CALCB | mRNA | -2.180 | 8.734E-06 | -1.800 | 4.453E-03 |
| ENSG00000176020 | AMIGO3 | mRNA | -1.126 | 3.019E-05 | -1.147 | 2.219E-04 |
| ENSG00000176046 | NUPR1 | mRNA | -1.569 | 2.126E-10 | -1.136 | 4.793E-04 |
| ENSG00000176092 | AIM1L | mRNA | 1.078 | 6.814E-03 | 1.822 | 1.420E-03 |
| ENSG00000176153 | GPX2 | mRNA | 2.231 | 5.081E-10 | 2.009 | 4.816E-06 |
| ENSG00000176381 | PRR18 | mRNA | -1.907 | 4.068E-03 | -1.977 | 2.394E-03 |
| ENSG00000176390 | CRLF3 | mRNA | 1.420 | 1.359E-09 | 1.188 | 1.245E-04 |
| ENSG00000176399 | DMRTA1 | mRNA | -2.017 | 7.822E-16 | -1.763 | 1.352E-09 |
| ENSG00000176402 | GJC3 | mRNA | -5.484 | 1.580E-16 | -4.963 | 5.397E-12 |
| ENSG00000176490 | DIRAS1 | mRNA | -3.511 | 4.628E-19 | -3.402 | 2.571E-14 |
| ENSG00000176533 | GNG7 | mRNA | -2.199 | 6.822E-24 | -1.342 | 3.216E-05 |
| ENSG00000176595 | KBTBD11 | mRNA | -2.368 | 2.528E-22 | -1.726 | 3.473E-08 |
| ENSG00000176659 | C20orf197 | mRNA | 1.819 | 1.012E-07 | 2.338 | 2.747E-14 |
| ENSG00000176678 | FOXL1 | mRNA | -2.384 | 7.159E-15 | -2.154 | 1.379E-05 |
| ENSG00000176692 | FOXC2 | mRNA | -3.036 | 6.153E-11 | -2.265 | 2.592E-05 |
| ENSG00000176697 | BDNF | mRNA | 1.569 | 5.209E-03 | 1.932 | 1.533E-06 |
| ENSG00000176842 | IRX5 | mRNA | -2.392 | 6.519E-10 | -1.962 | 1.449E-06 |
| ENSG00000176884 | GRIN1 | mRNA | -4.803 | 7.413E-59 | -2.891 | 4.610E-10 |
| ENSG00000176890 | TYMS | mRNA | 2.216 | 1.685E-07 | 1.773 | 7.478E-06 |
| ENSG00000176907 | C8orf4 | mRNA | 1.388 | 8.327E-08 | 1.144 | 1.751E-04 |
| ENSG00000176909 | MAMSTR | mRNA | -1.168 | 1.018E-05 | -1.053 | 3.602E-03 |
| ENSG00000176920 | FUT2 | mRNA | -1.065 | 1.007E-06 | -1.593 | 3.560E-06 |
| ENSG00000176971 | FIBIN | mRNA | -1.629 | 2.003E-05 | -1.891 | 1.351E-05 |
| ENSG00000177300 | CLDN22 | mRNA | -6.526 | 6.154E-26 | -6.010 | 4.915E-18 |
| ENSG00000177359 | RP11-551L14.1 | mRNA | 1.128 | 8.734E-03 | 1.423 | 3.334E-03 |
| ENSG00000177409 | SAMD9L | mRNA | 2.001 | 2.729E-07 | 1.726 | 3.597E-03 |
| ENSG00000177508 | IRX3 | mRNA | -1.552 | 8.369E-12 | -1.071 | 1.016E-04 |
| ENSG00000177575 | CD163 | mRNA | 2.400 | 1.023E-11 | 3.881 | 8.976E-18 |
| ENSG00000177602 | GSG2 | mRNA | 1.657 | 3.015E-04 | 1.378 | 1.640E-03 |
| ENSG00000177627 | C12orf54 | mRNA | 2.154 | 6.785E-04 | 2.025 | 4.255E-04 |
| ENSG00000177685 | EFCAB4A | mRNA | -3.534 | 1.927E-42 | -2.540 | 8.795E-11 |
| ENSG00000178031 | ADAMTSL1 | mRNA | -1.719 | 1.719E-07 | -1.189 | 9.111E-05 |
| ENSG00000178233 | TMEM151B | mRNA | -1.441 | 4.186E-03 | -1.599 | 5.871E-03 |
| ENSG00000178401 | DNAJC22 | mRNA | -1.681 | 1.718E-09 | -1.185 | 3.105E-04 |
| ENSG00000178473 | UCN3 | mRNA | -2.010 | 2.577E-04 | -2.446 | 1.213E-04 |
| ENSG00000178531 | CTXN1 | mRNA | -2.164 | 1.548E-03 | -1.989 | 3.967E-03 |
| ENSG00000178562 | CD28 | mRNA | 2.367 | 1.120E-10 | 2.032 | 1.076E-08 |
| ENSG00000178585 | CTNNBIP1 | mRNA | -1.794 | 3.913E-22 | -1.478 | 1.430E-08 |
| ENSG00000178773 | CPNE7 | mRNA | -1.163 | 5.523E-03 | -1.436 | 1.034E-03 |
| ENSG00000178789 | CD300LB | mRNA | 2.565 | 9.424E-09 | 3.409 | 2.402E-13 |
| ENSG00000178821 | TMEM52 | mRNA | -1.978 | 1.907E-08 | -1.682 | 1.113E-05 |
| ENSG00000178947 | LINC00086 | mRNA | -2.038 | 8.279E-13 | -1.193 | 1.197E-04 |
| ENSG00000178999 | AURKB | mRNA | 1.961 | 1.266E-05 | 1.869 | 2.413E-05 |
| ENSG00000179066 | AC020907.1 | mRNA | -2.696 | 2.854E-11 | -3.438 | 1.524E-13 |
| ENSG00000179097 | HTR1F | mRNA | 2.330 | 1.646E-07 | 1.855 | 1.264E-03 |
| ENSG00000179165 | PXT1 | mRNA | 3.254 | 4.764E-04 | 2.953 | 2.935E-03 |
| ENSG00000179178 | TMEM125 | mRNA | -1.700 | 4.230E-07 | -1.319 | 4.627E-05 |
| ENSG00000179331 | RAB39A | mRNA | 2.591 | 2.853E-10 | 1.910 | 1.082E-04 |
| ENSG00000179603 | GRM8 | mRNA | 2.527 | 1.593E-06 | 3.252 | 6.195E-13 |
| ENSG00000179636 | TPPP2 | mRNA | -3.181 | 6.595E-06 | -2.607 | 1.161E-04 |
| ENSG00000179639 | FCER1A | mRNA | 1.727 | 4.743E-06 | 1.454 | 2.038E-03 |
| ENSG00000179673 | RPRML | mRNA | -3.976 | 8.466E-08 | -2.778 | 2.197E-03 |
| ENSG00000179840 | C1orf200 | mRNA | 2.868 | 1.188E-03 | 3.619 | 1.913E-05 |
| ENSG00000179862 | CITED4 | mRNA | -2.940 | 3.651E-23 | -1.789 | 6.472E-06 |
| ENSG00000179913 | B3GNT3 | mRNA | -1.008 | 1.029E-05 | -1.538 | 2.633E-09 |
| ENSG00000179914 | ITLN1 | mRNA | -5.793 | 8.786E-09 | -2.318 | 2.147E-03 |
| ENSG00000179915 | NRXN1 | mRNA | -4.757 | 2.635E-24 | -5.236 | 1.502E-14 |
| ENSG00000179934 | CCR8 | mRNA | 3.020 | 6.765E-08 | 2.105 | 6.836E-04 |
| ENSG00000180061 | TMEM150B | mRNA | 1.741 | 7.071E-04 | 2.950 | 5.108E-09 |
| ENSG00000180316 | PNPLA1 | mRNA | 1.632 | 1.503E-04 | 2.528 | 2.000E-11 |
| ENSG00000180340 | FZD2 | mRNA | 1.273 | 7.655E-06 | 1.642 | 1.834E-07 |
| ENSG00000180347 | CCDC129 | mRNA | -5.255 | 1.028E-24 | -3.418 | 7.222E-11 |
| ENSG00000180353 | HCLS1 | mRNA | 1.040 | 1.982E-07 | 1.170 | 7.143E-07 |
| ENSG00000180535 | BHLHA15 | mRNA | -3.572 | 5.896E-28 | -2.035 | 4.248E-06 |
| ENSG00000180537 | RNF182 | mRNA | -2.078 | 1.961E-06 | -1.917 | 3.887E-05 |
| ENSG00000180660 | MAB21L1 | mRNA | 1.528 | 2.585E-05 | 1.163 | 9.596E-04 |
| ENSG00000180730 | SHISA2 | mRNA | -1.115 | 2.096E-02 | -1.396 | 4.124E-03 |
| ENSG00000180861 | C12orf36 | mRNA | 4.645 | 1.493E-12 | 4.188 | 1.524E-09 |
| ENSG00000180871 | CXCR2 | mRNA | 3.598 | 5.660E-20 | 2.970 | 2.534E-10 |
| ENSG00000180914 | OXTR | mRNA | 1.071 | 1.401E-02 | 2.448 | 5.653E-07 |
| ENSG00000181092 | ADIPOQ | mRNA | -3.957 | 9.440E-04 | -3.009 | 6.297E-03 |
| ENSG00000181104 | F2R | mRNA | 1.126 | 2.086E-07 | 1.028 | 2.605E-04 |
| ENSG00000181126 | HLA-V | mRNA | 2.059 | 2.634E-04 | 1.809 | 2.647E-03 |
| ENSG00000181234 | TMEM132C | mRNA | -3.182 | 2.883E-07 | -3.352 | 3.902E-06 |
| ENSG00000181374 | CCL13 | mRNA | 3.893 | 9.024E-06 | 5.212 | 8.656E-10 |
| ENSG00000181408 | UTS2R | mRNA | -1.085 | 1.695E-03 | 1.602 | 4.262E-03 |
| ENSG00000181495 | AC026703.1 | mRNA | 1.976 | 6.206E-04 | 1.569 | 4.374E-03 |
| ENSG00000181617 | FDCSP | mRNA | -3.260 | 3.449E-06 | -3.318 | 8.698E-06 |
| ENSG00000181631 | P2RY13 | mRNA | 2.551 | 2.909E-09 | 2.076 | 2.127E-04 |
| ENSG00000181690 | PLAG1 | mRNA | 1.384 | 7.913E-06 | 1.192 | 2.386E-03 |
| ENSG00000181751 | C5orf30 | mRNA | 1.341 | 3.318E-07 | 1.183 | 1.652E-04 |
| ENSG00000181804 | SLC9A9 | mRNA | 1.198 | 7.521E-07 | 1.020 | 7.920E-04 |
| ENSG00000181847 | TIGIT | mRNA | 2.037 | 3.879E-08 | 1.441 | 1.291E-04 |
| ENSG00000181963 | OR52K2 | mRNA | 2.425 | 2.396E-08 | 2.547 | 6.648E-07 |
| ENSG00000181982 | CCDC149 | mRNA | -1.245 | 4.633E-10 | -1.164 | 2.762E-07 |
| ENSG00000182010 | RTKN2 | mRNA | 2.738 | 8.221E-19 | 1.976 | 4.362E-06 |
| ENSG00000182103 | FAM181B | mRNA | -2.273 | 1.842E-10 | -1.951 | 4.172E-06 |
| ENSG00000182195 | LDOC1 | mRNA | -1.549 | 7.796E-12 | -1.317 | 6.302E-07 |
| ENSG00000182253 | SYNM | mRNA | -2.044 | 1.416E-18 | -2.010 | 1.001E-13 |
| ENSG00000182255 | KCNA4 | mRNA | -2.447 | 9.254E-04 | -3.821 | 2.868E-05 |
| ENSG00000182261 | NLRP10 | mRNA | 2.592 | 3.275E-04 | 4.221 | 2.055E-13 |
| ENSG00000182287 | AP1S2 | mRNA | 1.513 | 7.510E-06 | 1.243 | 5.600E-03 |
| ENSG00000182368 | FAM27A | mRNA | -1.633 | 5.008E-05 | -1.199 | 6.238E-03 |
| ENSG00000182379 | NXPH4 | mRNA | 2.442 | 3.162E-07 | 2.943 | 2.400E-10 |
| ENSG00000182472 | CAPN12 | mRNA | -2.204 | 9.616E-14 | -1.520 | 1.401E-05 |
| ENSG00000182481 | KPNA2 | mRNA | 1.591 | 2.272E-13 | 1.255 | 5.153E-05 |
| ENSG00000182487 | NCF1B | mRNA | 1.549 | 3.107E-06 | 2.648 | 1.045E-11 |
| ENSG00000182566 | CLEC4G | mRNA | 3.367 | 2.244E-05 | 6.130 | 3.261E-15 |
| ENSG00000182578 | CSF1R | mRNA | 1.327 | 2.156E-07 | 1.583 | 1.656E-10 |
| ENSG00000182580 | EPHB3 | mRNA | -2.383 | 6.006E-28 | -1.810 | 2.921E-10 |
| ENSG00000182601 | HS3ST4 | mRNA | 2.308 | 6.787E-09 | 2.208 | 5.958E-11 |
| ENSG00000182611 | HIST1H2AJ | mRNA | 2.521 | 8.909E-09 | 2.329 | 1.124E-07 |
| ENSG00000182621 | PLCB1 | mRNA | -1.034 | 2.972E-05 | -1.327 | 1.175E-05 |
| ENSG00000182732 | RGS6 | mRNA | -1.282 | 2.844E-05 | -1.472 | 6.383E-04 |
| ENSG00000182752 | PAPPA | mRNA | 2.120 | 2.442E-16 | 2.536 | 3.537E-18 |
| ENSG00000182851 | GPIHBP1 | mRNA | -2.646 | 3.816E-11 | -1.567 | 1.667E-04 |
| ENSG00000182885 | GPR97 | mRNA | 2.353 | 1.061E-08 | 3.484 | 9.349E-16 |
| ENSG00000182901 | RGS7 | mRNA | 2.181 | 8.405E-06 | 2.732 | 9.245E-08 |
| ENSG00000182938 | OTOP3 | mRNA | 3.865 | 1.307E-06 | 3.746 | 1.210E-05 |
| ENSG00000182963 | GJC1 | mRNA | 1.013 | 7.334E-05 | 1.108 | 3.520E-05 |
| ENSG00000182985 | CADM1 | mRNA | -1.579 | 3.405E-07 | -1.422 | 8.185E-05 |
| ENSG00000183010 | PYCR1 | mRNA | -3.121 | 2.898E-21 | -1.959 | 1.485E-08 |
| ENSG00000183019 | C19orf59 | mRNA | 3.603 | 2.703E-07 | 2.988 | 1.147E-06 |
| ENSG00000183048 | SLC25A10 | mRNA | -1.642 | 1.426E-12 | -1.324 | 7.045E-06 |
| ENSG00000183077 | AFMID | mRNA | -1.663 | 1.355E-14 | -1.113 | 1.044E-04 |
| ENSG00000183090 | FREM3 | mRNA | -5.589 | 1.819E-21 | -5.038 | 1.691E-26 |
| ENSG00000183111 | ARHGEF37 | mRNA | -2.071 | 7.047E-27 | -1.543 | 1.835E-10 |
| ENSG00000183134 | PTGDR2 | mRNA | 1.673 | 1.231E-02 | 3.264 | 5.221E-13 |
| ENSG00000183166 | CALN1 | mRNA | -3.188 | 2.252E-07 | -2.836 | 3.427E-04 |
| ENSG00000183250 | C21orf67 | mRNA | -1.450 | 2.341E-08 | -1.216 | 1.371E-04 |
| ENSG00000183287 | CCBE1 | mRNA | -2.880 | 1.110E-09 | -2.957 | 2.021E-10 |
| ENSG00000183317 | EPHA10 | mRNA | -2.903 | 6.304E-22 | -1.932 | 2.714E-05 |
| ENSG00000183395 | PMCH | mRNA | 3.429 | 5.333E-04 | 4.393 | 1.891E-07 |
| ENSG00000183454 | GRIN2A | mRNA | -1.188 | 1.063E-02 | -2.454 | 5.087E-06 |
| ENSG00000183476 | SH2D7 | mRNA | 2.863 | 1.223E-02 | 5.651 | 2.768E-14 |
| ENSG00000183625 | CCR3 | mRNA | 5.217 | 1.380E-10 | 6.124 | 1.485E-16 |
| ENSG00000183671 | GPR1 | mRNA | 2.798 | 4.054E-08 | 2.511 | 6.121E-04 |
| ENSG00000183715 | OPCML | mRNA | -2.636 | 8.993E-09 | -3.481 | 1.315E-15 |
| ENSG00000183742 | MACC1 | mRNA | 2.021 | 3.410E-15 | 1.050 | 4.862E-04 |
| ENSG00000183748 | MRC1L1 | mRNA | 2.099 | 4.909E-09 | 3.239 | 1.596E-19 |
| ENSG00000183778 | B3GALT5 | mRNA | -1.047 | 1.433E-02 | -2.704 | 6.140E-11 |
| ENSG00000183780 | SLC35F3 | mRNA | -1.200 | 4.426E-03 | -1.174 | 6.501E-03 |
| ENSG00000183798 | EMILIN3 | mRNA | -2.712 | 2.914E-11 | -1.760 | 8.385E-06 |
| ENSG00000183813 | CCR4 | mRNA | 1.698 | 5.268E-06 | 1.765 | 3.558E-06 |
| ENSG00000183844 | FAM3B | mRNA | -1.645 | 2.506E-06 | -2.235 | 3.319E-06 |
| ENSG00000183856 | IQGAP3 | mRNA | 2.065 | 7.361E-07 | 1.650 | 2.776E-04 |
| ENSG00000183888 | C1orf64 | mRNA | -4.050 | 5.841E-14 | -2.155 | 2.333E-05 |
| ENSG00000183960 | KCNH8 | mRNA | -2.529 | 5.075E-10 | -2.820 | 1.200E-09 |
| ENSG00000184012 | TMPRSS2 | mRNA | -1.486 | 2.193E-11 | -1.141 | 2.120E-07 |
| ENSG00000184144 | CNTN2 | mRNA | -5.397 | 1.730E-28 | -5.681 | 1.477E-22 |
| ENSG00000184221 | OLIG1 | mRNA | 2.599 | 2.893E-03 | 3.935 | 4.245E-09 |
| ENSG00000184258 | CDR1 | mRNA | -3.590 | 1.372E-13 | -3.132 | 3.271E-08 |
| ENSG00000184313 | MROH7 | mRNA | -2.041 | 1.741E-13 | -1.347 | 3.417E-04 |
| ENSG00000184357 | HIST1H1B | mRNA | 2.001 | 7.860E-09 | 1.716 | 1.465E-06 |
| ENSG00000184454 | NCMAP | mRNA | -1.555 | 1.383E-05 | -1.764 | 1.892E-06 |
| ENSG00000184530 | C6orf58 | mRNA | -7.392 | 1.971E-32 | -5.702 | 2.445E-12 |
| ENSG00000184599 | FAM19A3 | mRNA | -2.149 | 1.427E-03 | -2.001 | 2.166E-03 |
| ENSG00000184601 | C14orf180 | mRNA | -2.661 | 8.911E-08 | -1.548 | 4.035E-03 |
| ENSG00000184613 | NELL2 | mRNA | -1.727 | 2.111E-06 | -2.387 | 6.397E-08 |
| ENSG00000184661 | CDCA2 | mRNA | 2.712 | 4.212E-10 | 2.118 | 1.310E-05 |
| ENSG00000184672 | RALYL | mRNA | -4.829 | 8.338E-09 | -4.698 | 4.586E-08 |
| ENSG00000184709 | LRRC26 | mRNA | -6.792 | 8.042E-71 | -4.470 | 1.295E-17 |
| ENSG00000184825 | HIST1H2AH | mRNA | 2.049 | 4.823E-08 | 1.539 | 7.078E-05 |
| ENSG00000184838 | PRR16 | mRNA | 3.630 | 1.033E-12 | 3.481 | 6.287E-14 |
| ENSG00000184905 | TCEAL2 | mRNA | -2.288 | 3.185E-11 | -1.627 | 6.743E-05 |
| ENSG00000185022 | MAFF | mRNA | -2.682 | 2.016E-06 | -1.561 | 5.306E-03 |
| ENSG00000185046 | ANKS1B | mRNA | -1.322 | 1.780E-07 | -1.467 | 1.255E-06 |
| ENSG00000185130 | HIST1H2BL | mRNA | 3.228 | 4.277E-12 | 2.235 | 3.445E-05 |
| ENSG00000185133 | INPP5J | mRNA | -1.810 | 9.559E-09 | -1.767 | 2.528E-06 |
| ENSG00000185156 | MFSD6L | mRNA | -1.569 | 1.276E-07 | -1.447 | 1.591E-05 |
| ENSG00000185201 | IFITM2 | mRNA | 2.302 | 5.717E-21 | 2.055 | 1.130E-08 |
| ENSG00000185271 | KLHL33 | mRNA | -1.718 | 4.891E-07 | -1.358 | 2.290E-03 |
| ENSG00000185274 | WBSCR17 | mRNA | -3.490 | 1.832E-12 | -1.719 | 3.492E-04 |
| ENSG00000185290 | NUPR1L | mRNA | -1.458 | 3.608E-03 | -1.526 | 4.213E-03 |
| ENSG00000185442 | FAM174B | mRNA | -2.363 | 2.787E-27 | -1.777 | 3.305E-14 |
| ENSG00000185480 | PARPBP | mRNA | 2.473 | 4.471E-10 | 1.639 | 1.509E-03 |
| ENSG00000185559 | DLK1 | mRNA | -6.307 | 1.411E-16 | -3.258 | 7.870E-04 |
| ENSG00000185624 | P4HB | mRNA | -1.661 | 1.852E-15 | -1.235 | 2.312E-07 |
| ENSG00000185633 | NDUFA4L2 | mRNA | 1.258 | 3.531E-05 | 1.817 | 5.501E-10 |
| ENSG00000185737 | NRG3 | mRNA | -5.071 | 4.556E-34 | -4.231 | 1.658E-17 |
| ENSG00000185758 | CLDN24 | mRNA | -5.978 | 1.876E-10 | -5.323 | 2.077E-09 |
| ENSG00000185811 | IKZF1 | mRNA | 1.525 | 8.872E-10 | 1.546 | 7.411E-08 |
| ENSG00000185862 | EVI2B | mRNA | 2.790 | 9.304E-07 | 2.306 | 1.006E-03 |
| ENSG00000185863 | TMEM210 | mRNA | -4.210 | 1.083E-11 | -3.087 | 1.265E-06 |
| ENSG00000185885 | IFITM1 | mRNA | 1.819 | 8.160E-14 | 1.702 | 7.549E-05 |
| ENSG00000185897 | FFAR3 | mRNA | 1.840 | 1.278E-03 | 5.331 | 1.308E-13 |
| ENSG00000185924 | RTN4RL1 | mRNA | -2.565 | 2.160E-19 | -1.854 | 1.619E-08 |
| ENSG00000185947 | ZNF267 | mRNA | 1.752 | 6.409E-13 | 1.218 | 9.132E-04 |
| ENSG00000186049 | KRT73 | mRNA | 3.092 | 1.320E-03 | 3.462 | 3.598E-04 |
| ENSG00000186074 | CD300LF | mRNA | 2.665 | 1.428E-10 | 3.220 | 4.402E-11 |
| ENSG00000186185 | KIF18B | mRNA | 1.359 | 3.843E-03 | 1.527 | 2.700E-03 |
| ENSG00000186190 | BPIFB3 | mRNA | -6.409 | 4.950E-17 | -4.935 | 2.420E-13 |
| ENSG00000186205 | 43525.000 | mRNA | -1.820 | 2.168E-15 | -1.909 | 7.187E-13 |
| ENSG00000186281 | GPAT2 | mRNA | -3.427 | 1.636E-23 | -1.810 | 6.129E-06 |
| ENSG00000186340 | THBS2 | mRNA | 1.167 | 3.576E-04 | 2.194 | 8.663E-09 |
| ENSG00000186395 | KRT10 | mRNA | 2.120 | 2.382E-06 | 1.566 | 6.113E-03 |
| ENSG00000186407 | CD300E | mRNA | 3.608 | 1.700E-10 | 2.237 | 7.483E-05 |
| ENSG00000186431 | FCAR | mRNA | 2.367 | 3.487E-05 | 1.914 | 6.001E-04 |
| ENSG00000186439 | TRDN | mRNA | -4.585 | 1.053E-06 | -3.955 | 1.328E-05 |
| ENSG00000186469 | GNG2 | mRNA | 1.653 | 4.520E-07 | 1.430 | 5.747E-04 |
| ENSG00000186493 | C5orf38 | mRNA | -3.586 | 3.079E-17 | -1.965 | 7.730E-04 |
| ENSG00000186642 | PDE2A | mRNA | -2.231 | 4.105E-15 | -1.290 | 8.043E-04 |
| ENSG00000186684 | CYP27C1 | mRNA | 1.807 | 1.317E-03 | 1.644 | 2.839E-03 |
| ENSG00000186714 | CCDC73 | mRNA | 4.480 | 4.021E-12 | 2.924 | 3.369E-03 |
| ENSG00000186732 | MPPED1 | mRNA | -1.547 | 7.170E-04 | -1.198 | 6.216E-03 |
| ENSG00000186806 | VSIG10L | mRNA | -4.123 | 5.438E-31 | -3.088 | 3.256E-15 |
| ENSG00000186807 | ANXA8L2 | mRNA | 1.580 | 3.039E-05 | 1.940 | 6.638E-07 |
| ENSG00000186818 | LILRB4 | mRNA | 2.780 | 6.498E-14 | 2.810 | 2.460E-14 |
| ENSG00000186868 | MAPT | mRNA | -1.465 | 5.732E-08 | -1.001 | 1.577E-03 |
| ENSG00000186871 | ERCC6L | mRNA | 3.236 | 1.128E-12 | 2.245 | 2.227E-04 |
| ENSG00000186907 | RTN4RL2 | mRNA | -2.431 | 2.269E-12 | -1.710 | 5.409E-06 |
| ENSG00000187037 | GPR141 | mRNA | 2.981 | 1.255E-12 | 2.479 | 2.539E-05 |
| ENSG00000187068 | C3orf70 | mRNA | -1.053 | 9.896E-04 | -1.226 | 1.083E-03 |
| ENSG00000187116 | LILRA5 | mRNA | 2.079 | 1.423E-05 | 1.873 | 1.656E-03 |
| ENSG00000187140 | FOXD3 | mRNA | -5.987 | 2.674E-17 | -6.377 | 4.697E-17 |
| ENSG00000187323 | DCC | mRNA | 2.363 | 1.470E-06 | 2.415 | 3.327E-05 |
| ENSG00000187474 | FPR3 | mRNA | 3.016 | 4.554E-21 | 2.300 | 2.700E-11 |
| ENSG00000187527 | ATP13A5 | mRNA | 2.374 | 5.327E-05 | 2.725 | 2.307E-08 |
| ENSG00000187566 | NHLRC1 | mRNA | -1.258 | 2.168E-05 | -1.143 | 7.796E-04 |
| ENSG00000187595 | ZNF385C | mRNA | -2.152 | 6.909E-12 | -1.869 | 1.405E-06 |
| ENSG00000187699 | C2orf88 | mRNA | -1.582 | 3.282E-05 | -1.784 | 1.564E-04 |
| ENSG00000187758 | ADH1A | mRNA | 2.340 | 9.404E-04 | 2.171 | 8.331E-03 |
| ENSG00000187773 | FAM69C | mRNA | -4.837 | 4.613E-09 | -3.775 | 1.020E-06 |
| ENSG00000187840 | EIF4EBP1 | mRNA | -1.601 | 5.144E-15 | -1.133 | 2.338E-06 |
| ENSG00000187848 | P2RX2 | mRNA | -2.903 | 1.363E-03 | -2.443 | 5.137E-03 |
| ENSG00000187867 | PALM3 | mRNA | -2.314 | 5.025E-09 | -1.178 | 8.597E-03 |
| ENSG00000187908 | DMBT1 | mRNA | -6.820 | 5.676E-36 | -4.980 | 3.300E-10 |
| ENSG00000187912 | CLEC17A | mRNA | 1.504 | 3.396E-04 | 1.408 | 1.138E-03 |
| ENSG00000187950 | OVCH1 | mRNA | 1.386 | 1.217E-02 | 2.033 | 2.775E-04 |
| ENSG00000187955 | COL14A1 | mRNA | -1.325 | 5.143E-10 | -1.222 | 1.076E-05 |
| ENSG00000187990 | HIST1H2BG | mRNA | 2.163 | 6.331E-09 | 1.403 | 1.532E-03 |
| ENSG00000188013 | MEIS3P2 | mRNA | -1.332 | 2.195E-04 | -1.369 | 8.838E-04 |
| ENSG00000188060 | RAB42 | mRNA | 1.235 | 1.044E-03 | 1.452 | 5.193E-05 |
| ENSG00000188257 | PLA2G2A | mRNA | -5.347 | 3.112E-12 | -6.401 | 3.274E-11 |
| ENSG00000188269 | OR7A5 | mRNA | 1.378 | 1.575E-02 | 2.815 | 1.029E-08 |
| ENSG00000188277 | C15orf62 | mRNA | -2.044 | 1.939E-07 | -1.858 | 3.802E-05 |
| ENSG00000188373 | C10orf99 | mRNA | 3.404 | 1.701E-03 | 4.189 | 5.550E-04 |
| ENSG00000188386 | PPP3R2 | mRNA | -4.674 | 5.619E-08 | -4.543 | 2.536E-07 |
| ENSG00000188396 | TCTEX1D4 | mRNA | -2.080 | 1.167E-03 | -1.819 | 3.695E-03 |
| ENSG00000188404 | SELL | mRNA | 1.583 | 4.328E-08 | 1.433 | 2.733E-04 |
| ENSG00000188487 | INSC | mRNA | -4.503 | 1.559E-17 | -3.451 | 3.063E-10 |
| ENSG00000188505 | NCCRP1 | mRNA | 2.820 | 3.915E-07 | 2.671 | 6.707E-05 |
| ENSG00000188517 | COL25A1 | mRNA | -1.731 | 7.405E-07 | -1.941 | 1.118E-05 |
| ENSG00000188649 | CC2D2B | mRNA | 1.717 | 5.034E-04 | 1.583 | 1.878E-03 |
| ENSG00000188672 | RHCE | mRNA | -2.402 | 1.601E-05 | -1.570 | 4.270E-03 |
| ENSG00000188676 | IDO2 | mRNA | 2.525 | 3.184E-05 | 2.491 | 1.863E-05 |
| ENSG00000188729 | OSTN | mRNA | -4.358 | 4.364E-06 | -3.327 | 1.985E-04 |
| ENSG00000188761 | BCL2L15 | mRNA | 1.961 | 3.990E-12 | 1.408 | 6.868E-06 |
| ENSG00000188766 | SPRED3 | mRNA | 1.381 | 7.940E-03 | 1.888 | 1.629E-07 |
| ENSG00000188783 | PRELP | mRNA | -1.429 | 1.039E-04 | -1.457 | 2.241E-04 |
| ENSG00000188833 | ENTPD8 | mRNA | -2.989 | 1.010E-07 | -2.712 | 4.860E-08 |
| ENSG00000188993 | LRRC66 | mRNA | -2.165 | 3.985E-03 | -2.688 | 1.960E-04 |
| ENSG00000189001 | SBSN | mRNA | 4.507 | 7.953E-05 | 7.449 | 3.644E-07 |
| ENSG00000189056 | RELN | mRNA | -2.325 | 4.065E-07 | -1.920 | 5.123E-04 |
| ENSG00000189057 | FAM111B | mRNA | 3.492 | 1.189E-13 | 2.335 | 7.446E-05 |
| ENSG00000189068 | VSTM1 | mRNA | 5.043 | 6.939E-08 | 6.592 | 1.280E-20 |
| ENSG00000189127 | ANKRD34B | mRNA | 4.879 | 1.437E-06 | 2.863 | 4.286E-03 |
| ENSG00000189292 | FAM150B | mRNA | -2.375 | 6.012E-07 | -2.277 | 2.006E-05 |
| ENSG00000189377 | CXCL17 | mRNA | -1.143 | 8.990E-06 | -1.585 | 1.860E-06 |
| ENSG00000189423 | USP32P3 | mRNA | 1.692 | 1.083E-04 | 1.282 | 4.913E-03 |
| ENSG00000189430 | NCR1 | mRNA | 2.164 | 9.650E-08 | 1.857 | 6.500E-06 |
| ENSG00000196083 | IL1RAP | mRNA | 1.672 | 5.811E-14 | 1.473 | 1.653E-05 |
| ENSG00000196091 | MYBPC1 | mRNA | -5.906 | 8.650E-20 | -3.238 | 3.283E-04 |
| ENSG00000196187 | TMEM63A | mRNA | -1.876 | 2.420E-21 | -1.470 | 1.209E-08 |
| ENSG00000196196 | HRCT1 | mRNA | -2.088 | 7.316E-07 | -1.435 | 5.266E-03 |
| ENSG00000196209 | SIRPB2 | mRNA | 2.133 | 8.336E-06 | 3.254 | 1.722E-10 |
| ENSG00000196226 | HIST1H2BB | mRNA | 3.799 | 3.029E-11 | 2.923 | 5.671E-05 |
| ENSG00000196331 | HIST1H2BO | mRNA | 4.236 | 1.224E-16 | 3.280 | 6.973E-10 |
| ENSG00000196344 | ADH7 | mRNA | 1.538 | 1.738E-05 | 1.177 | 3.085E-03 |
| ENSG00000196374 | HIST1H2BM | mRNA | 4.135 | 2.068E-15 | 3.327 | 2.646E-08 |
| ENSG00000196376 | SLC35F1 | mRNA | -2.097 | 2.755E-07 | -1.778 | 1.510E-05 |
| ENSG00000196408 | NOXO1 | mRNA | -3.330 | 1.113E-13 | -2.441 | 8.289E-06 |
| ENSG00000196482 | ESRRG | mRNA | -1.496 | 7.030E-05 | -1.885 | 7.006E-06 |
| ENSG00000196532 | HIST1H3C | mRNA | 2.791 | 1.033E-08 | 2.567 | 7.063E-09 |
| ENSG00000196533 | C1orf186 | mRNA | 1.059 | 4.214E-03 | 1.058 | 7.217E-03 |
| ENSG00000196611 | MMP1 | mRNA | 4.523 | 5.408E-11 | 3.220 | 1.218E-05 |
| ENSG00000196639 | HRH1 | mRNA | 1.093 | 1.863E-03 | 2.262 | 3.435E-11 |
| ENSG00000196735 | HLA-DQA1 | mRNA | 1.469 | 5.030E-07 | 1.028 | 4.645E-04 |
| ENSG00000196743 | GM2A | mRNA | 1.385 | 2.875E-13 | 1.141 | 5.859E-05 |
| ENSG00000196747 | HIST1H2AI | mRNA | 2.154 | 1.104E-09 | 1.914 | 2.289E-07 |
| ENSG00000196787 | HIST1H2AG | mRNA | 2.263 | 1.011E-11 | 1.901 | 1.129E-08 |
| ENSG00000196834 | POTEI | mRNA | -2.272 | 7.253E-05 | -2.493 | 5.381E-05 |
| ENSG00000196866 | HIST1H2AD | mRNA | 2.412 | 1.217E-03 | 3.086 | 1.006E-03 |
| ENSG00000196872 | KIAA1211L | mRNA | -1.696 | 3.047E-05 | -1.362 | 8.486E-04 |
| ENSG00000196890 | HIST3H2BB | mRNA | 2.995 | 9.783E-11 | 1.740 | 3.118E-04 |
| ENSG00000197153 | HIST1H3J | mRNA | 3.385 | 3.444E-10 | 2.709 | 2.071E-07 |
| ENSG00000197238 | HIST1H4J | mRNA | 2.858 | 1.883E-15 | 1.981 | 8.453E-06 |
| ENSG00000197253 | TPSB2 | mRNA | 1.177 | 8.047E-03 | 1.682 | 9.302E-05 |
| ENSG00000197261 | C6orf141 | mRNA | -1.767 | 1.387E-08 | -1.567 | 3.907E-06 |
| ENSG00000197262 | CCL4L2 | mRNA | 2.591 | 2.621E-05 | 3.748 | 1.644E-07 |
| ENSG00000197272 | IL27 | mRNA | 3.146 | 3.877E-04 | 3.851 | 2.698E-03 |
| ENSG00000197299 | BLM | mRNA | 2.195 | 8.288E-10 | 1.733 | 1.205E-04 |
| ENSG00000197361 | FBXL22 | mRNA | -2.091 | 1.958E-09 | -1.858 | 9.276E-06 |
| ENSG00000197409 | HIST1H3D | mRNA | 1.465 | 9.067E-08 | 1.169 | 5.505E-05 |
| ENSG00000197459 | HIST1H2BH | mRNA | 3.392 | 2.381E-14 | 2.643 | 7.558E-07 |
| ENSG00000197506 | SLC28A3 | mRNA | -1.342 | 2.031E-05 | -1.794 | 2.568E-07 |
| ENSG00000197629 | MPEG1 | mRNA | 2.091 | 1.446E-16 | 1.267 | 1.039E-06 |
| ENSG00000197632 | SERPINB2 | mRNA | 3.814 | 2.367E-07 | 5.103 | 6.528E-14 |
| ENSG00000197641 | SERPINB13 | mRNA | 3.380 | 2.213E-16 | 2.780 | 1.237E-08 |
| ENSG00000197697 | HIST1H2BE | mRNA | 3.023 | 1.770E-12 | 2.075 | 6.102E-05 |
| ENSG00000197704 | FRMPD2P1 | mRNA | -1.746 | 9.779E-06 | -1.415 | 1.821E-03 |
| ENSG00000197705 | KLHL14 | mRNA | -1.385 | 3.666E-07 | -1.638 | 1.026E-06 |
| ENSG00000197723 | HSPB9 | mRNA | -3.760 | 1.687E-10 | -2.873 | 3.770E-07 |
| ENSG00000197747 | S100A10 | mRNA | 1.846 | 5.199E-13 | 1.546 | 5.178E-06 |
| ENSG00000197769 | MAP1LC3C | mRNA | 1.416 | 2.711E-03 | 1.631 | 4.929E-04 |
| ENSG00000197846 | HIST1H2BF | mRNA | 3.395 | 7.276E-14 | 2.505 | 2.019E-06 |
| ENSG00000197870 | PRB3 | mRNA | -10.298 | 5.964E-19 | -9.594 | 1.450E-16 |
| ENSG00000197903 | HIST1H2BK | mRNA | 2.095 | 7.336E-15 | 1.496 | 5.973E-06 |
| ENSG00000197914 | HIST1H4K | mRNA | 2.734 | 3.903E-07 | 1.682 | 7.524E-03 |
| ENSG00000198074 | AKR1B10 | mRNA | 2.097 | 3.855E-06 | 2.199 | 2.844E-04 |
| ENSG00000198125 | MB | mRNA | -1.810 | 5.611E-06 | -1.906 | 4.103E-10 |
| ENSG00000198171 | DDRGK1 | mRNA | -1.425 | 5.473E-08 | -1.008 | 1.133E-03 |
| ENSG00000198183 | BPIFA1 | mRNA | -3.929 | 7.684E-13 | -5.289 | 3.181E-18 |
| ENSG00000198216 | CACNA1E | mRNA | -3.513 | 9.355E-13 | -3.755 | 1.030E-11 |
| ENSG00000198223 | CSF2RA | mRNA | 1.229 | 5.705E-06 | 1.708 | 5.854E-08 |
| ENSG00000198327 | HIST1H4F | mRNA | 2.059 | 3.829E-09 | 1.287 | 2.276E-03 |
| ENSG00000198339 | HIST1H4I | mRNA | 2.344 | 4.149E-08 | 1.606 | 3.740E-05 |
| ENSG00000198354 | DCAF12L2 | mRNA | -3.127 | 8.970E-10 | -2.841 | 3.043E-07 |
| ENSG00000198374 | HIST1H2AL | mRNA | 2.398 | 2.614E-08 | 1.440 | 2.219E-04 |
| ENSG00000198400 | NTRK1 | mRNA | 1.634 | 1.500E-03 | 2.966 | 6.114E-10 |
| ENSG00000198478 | SH3BGRL2 | mRNA | -1.607 | 1.392E-06 | -2.096 | 9.100E-09 |
| ENSG00000198515 | CNGA1 | mRNA | -2.119 | 6.425E-05 | -2.034 | 2.261E-04 |
| ENSG00000198535 | C2CD4A | mRNA | -1.524 | 3.598E-03 | -1.444 | 7.290E-03 |
| ENSG00000198558 | HIST1H4L | mRNA | 3.638 | 2.190E-12 | 2.683 | 3.819E-05 |
| ENSG00000198574 | SH2D1B | mRNA | 2.929 | 4.402E-12 | 2.378 | 7.503E-06 |
| ENSG00000198626 | RYR2 | mRNA | -1.588 | 5.450E-09 | -2.028 | 1.345E-08 |
| ENSG00000198643 | FAM3D | mRNA | -2.961 | 4.306E-29 | -3.194 | 7.087E-19 |
| ENSG00000198648 | STK39 | mRNA | -1.115 | 2.236E-06 | -1.435 | 2.043E-07 |
| ENSG00000198734 | F5 | mRNA | -3.641 | 5.010E-18 | -3.616 | 1.783E-12 |
| ENSG00000198739 | LRRTM3 | mRNA | -3.635 | 3.184E-10 | -2.298 | 7.608E-04 |
| ENSG00000198743 | SLC5A3 | mRNA | 2.574 | 3.403E-05 | 2.338 | 4.991E-04 |
| ENSG00000198753 | PLXNB3 | mRNA | -2.528 | 1.671E-27 | -1.601 | 5.858E-07 |
| ENSG00000198756 | COLGALT2 | mRNA | -1.995 | 1.446E-10 | -2.297 | 6.986E-10 |
| ENSG00000198759 | EGFL6 | mRNA | 1.621 | 7.219E-04 | 1.252 | 7.321E-03 |
| ENSG00000198771 | RCSD1 | mRNA | 1.241 | 5.066E-08 | 1.420 | 6.523E-10 |
| ENSG00000198774 | RASSF9 | mRNA | 1.268 | 1.725E-09 | 1.188 | 8.425E-05 |
| ENSG00000198785 | GRIN3A | mRNA | -2.777 | 1.394E-07 | -3.926 | 1.467E-09 |
| ENSG00000198796 | ALPK2 | mRNA | 4.622 | 1.030E-16 | 4.827 | 1.117E-18 |
| ENSG00000198797 | BRINP2 | mRNA | -1.856 | 3.197E-03 | -2.834 | 5.024E-05 |
| ENSG00000198821 | CD247 | mRNA | 1.343 | 1.266E-05 | 1.236 | 4.341E-05 |
| ENSG00000198826 | ARHGAP11A | mRNA | 2.087 | 1.844E-12 | 1.582 | 2.823E-05 |
| ENSG00000198829 | SUCNR1 | mRNA | 2.755 | 6.480E-05 | 3.474 | 3.048E-07 |
| ENSG00000198838 | RYR3 | mRNA | -2.532 | 1.478E-09 | -2.697 | 2.826E-08 |
| ENSG00000198848 | CES1 | mRNA | -2.026 | 1.812E-05 | -1.282 | 3.705E-03 |
| ENSG00000198901 | PRC1 | mRNA | 1.382 | 5.156E-05 | 1.067 | 5.308E-03 |
| ENSG00000198910 | L1CAM | mRNA | -1.987 | 2.806E-09 | -1.799 | 3.736E-06 |
| ENSG00000198944 | SOWAHA | mRNA | -2.087 | 1.197E-05 | -1.572 | 6.418E-03 |
| ENSG00000203710 | CR1 | mRNA | 3.659 | 2.115E-35 | 4.126 | 5.694E-35 |
| ENSG00000203722 | RAET1G | mRNA | 1.468 | 2.391E-04 | 1.638 | 1.758E-03 |
| ENSG00000203727 | SAMD5 | mRNA | 1.877 | 4.986E-14 | 2.112 | 5.953E-10 |
| ENSG00000203747 | FCGR3A | mRNA | 2.968 | 3.106E-11 | 2.811 | 4.918E-08 |
| ENSG00000203785 | SPRR2E | mRNA | 5.088 | 6.825E-08 | 5.678 | 8.431E-05 |
| ENSG00000203805 | PPAPDC1A | mRNA | 2.963 | 4.743E-18 | 3.569 | 1.014E-14 |
| ENSG00000203811 | HIST2H3C | mRNA | 2.342 | 1.775E-07 | 1.977 | 1.169E-05 |
| ENSG00000203813 | HIST1H3H | mRNA | 2.103 | 1.265E-09 | 1.405 | 1.430E-04 |
| ENSG00000203852 | HIST2H3A | mRNA | 2.342 | 1.774E-07 | 1.977 | 1.169E-05 |
| ENSG00000203930 | LINC00632 | mRNA | -3.705 | 3.221E-15 | -3.228 | 1.236E-09 |
| ENSG00000204020 | LIPN | mRNA | 4.411 | 4.728E-09 | 6.150 | 4.872E-12 |
| ENSG00000204065 | TCEAL5 | mRNA | -2.251 | 1.833E-03 | -3.790 | 3.453E-05 |
| ENSG00000204071 | TCEAL6 | mRNA | -3.671 | 2.519E-07 | -2.353 | 1.204E-03 |
| ENSG00000204128 | C2orf72 | mRNA | -1.992 | 2.304E-04 | -3.608 | 1.036E-11 |
| ENSG00000204161 | C10orf128 | mRNA | 1.050 | 7.217E-05 | 1.930 | 2.776E-10 |
| ENSG00000204175 | GPRIN2 | mRNA | -1.855 | 4.558E-08 | -1.202 | 7.414E-04 |
| ENSG00000204219 | TCEA3 | mRNA | -1.940 | 5.005E-16 | -1.500 | 5.652E-08 |
| ENSG00000204248 | COL11A2 | mRNA | -2.365 | 2.789E-09 | -1.174 | 6.141E-03 |
| ENSG00000204335 | SP5 | mRNA | -3.558 | 5.723E-14 | -2.647 | 1.502E-07 |
| ENSG00000204361 | NXPE2 | mRNA | -4.088 | 8.656E-16 | -4.152 | 1.119E-12 |
| ENSG00000204381 | LAYN | mRNA | 1.056 | 1.647E-06 | 1.031 | 8.930E-04 |
| ENSG00000204472 | AIF1 | mRNA | 2.353 | 1.465E-14 | 2.153 | 2.774E-09 |
| ENSG00000204482 | LST1 | mRNA | 2.735 | 3.952E-15 | 3.007 | 4.873E-12 |
| ENSG00000204516 | MICB | mRNA | 1.665 | 1.428E-06 | 1.327 | 2.856E-04 |
| ENSG00000204544 | MUC21 | mRNA | 2.010 | 1.059E-03 | 2.824 | 9.879E-05 |
| ENSG00000204577 | LILRB3 | mRNA | 1.712 | 2.440E-05 | 1.745 | 6.834E-06 |
| ENSG00000204922 | C11orf83 | mRNA | -1.695 | 5.788E-09 | -1.084 | 1.611E-03 |
| ENSG00000204941 | PSG5 | mRNA | 3.387 | 3.106E-04 | 4.911 | 1.086E-05 |
| ENSG00000204983 | PRSS1 | mRNA | -4.057 | 1.821E-10 | -4.238 | 7.988E-08 |
| ENSG00000205020 | CCL4L1 | mRNA | 1.833 | 6.053E-05 | 2.824 | 1.447E-07 |
| ENSG00000205021 | CCL3L1 | mRNA | 2.388 | 5.982E-04 | 3.002 | 9.679E-05 |
| ENSG00000205060 | SLC35B4 | mRNA | 1.558 | 6.894E-12 | 1.120 | 3.250E-04 |
| ENSG00000205076 | LGALS7 | mRNA | 2.672 | 3.169E-05 | 3.198 | 1.147E-06 |
| ENSG00000205403 | CFI | mRNA | 2.288 | 1.605E-27 | 2.312 | 1.807E-13 |
| ENSG00000205413 | SAMD9 | mRNA | 2.426 | 6.485E-12 | 2.034 | 2.995E-04 |
| ENSG00000205420 | KRT6A | mRNA | 2.325 | 4.263E-03 | 2.823 | 1.141E-03 |
| ENSG00000205476 | CCDC85C | mRNA | -1.650 | 6.907E-14 | -1.052 | 2.897E-04 |
| ENSG00000205542 | TMSB4X | mRNA | 1.554 | 8.573E-13 | 1.191 | 1.021E-04 |
| ENSG00000205592 | MUC19 | mRNA | -2.757 | 8.219E-05 | -4.443 | 4.584E-08 |
| ENSG00000205649 | HTN3 | mRNA | -6.524 | 9.745E-14 | -4.084 | 1.526E-08 |
| ENSG00000205710 | C17orf107 | mRNA | -2.409 | 9.004E-16 | -1.154 | 5.319E-04 |
| ENSG00000205835 | GMNC | mRNA | 3.696 | 6.873E-08 | 1.786 | 4.991E-03 |
| ENSG00000205923 | CEMP1 | mRNA | -1.668 | 1.405E-06 | -1.160 | 2.864E-03 |
| ENSG00000205927 | OLIG2 | mRNA | 6.544 | 2.689E-07 | 7.831 | 3.485E-18 |
| ENSG00000206013 | IFITM5 | mRNA | -6.343 | 4.600E-05 | -4.700 | 3.405E-03 |
| ENSG00000206069 | TMEM211 | mRNA | -2.490 | 5.518E-08 | -1.939 | 4.026E-05 |
| ENSG00000206073 | SERPINB4 | mRNA | 4.063 | 3.997E-36 | 3.064 | 4.963E-10 |
| ENSG00000206075 | SERPINB5 | mRNA | 1.704 | 4.301E-05 | 1.465 | 2.612E-03 |
| ENSG00000206150 | RNASE13 | mRNA | -3.569 | 1.130E-05 | -3.942 | 2.801E-05 |
| ENSG00000206384 | COL6A6 | mRNA | 1.458 | 1.994E-02 | 2.582 | 1.891E-07 |
| ENSG00000206432 | TMEM200C | mRNA | -1.935 | 2.348E-11 | -1.158 | 1.456E-03 |
| ENSG00000206538 | VGLL3 | mRNA | 1.492 | 4.784E-08 | 1.789 | 3.684E-08 |
| ENSG00000206549 | PRSS50 | mRNA | -1.438 | 2.089E-05 | -1.144 | 7.778E-03 |
| ENSG00000206579 | XKR4 | mRNA | -1.135 | 1.051E-02 | -2.835 | 2.036E-08 |
| ENSG00000211640 | IGLV6-57 | mRNA | 2.019 | 1.201E-05 | 1.819 | 1.074E-04 |
| ENSG00000211660 | IGLV2-23 | mRNA | 1.836 | 1.430E-05 | 2.010 | 1.497E-05 |
| ENSG00000211668 | IGLV2-11 | mRNA | 1.212 | 2.595E-04 | 2.054 | 8.098E-05 |
| ENSG00000211673 | IGLV3-1 | mRNA | 1.620 | 9.801E-06 | 2.614 | 5.313E-08 |
| ENSG00000211689 | TRGC1 | mRNA | 1.713 | 2.800E-05 | 1.721 | 1.255E-03 |
| ENSG00000211893 | IGHG2 | mRNA | 2.651 | 1.325E-11 | 3.047 | 3.798E-10 |
| ENSG00000211896 | IGHG1 | mRNA | 1.314 | 3.075E-04 | 2.127 | 3.421E-06 |
| ENSG00000211953 | IGHV3-30 | mRNA | 1.307 | 4.724E-04 | 1.456 | 2.172E-03 |
| ENSG00000211956 | IGHV4-34 | mRNA | 2.358 | 7.088E-06 | 1.865 | 1.485E-03 |
| ENSG00000211962 | IGHV1-46 | mRNA | 1.040 | 1.696E-02 | 1.286 | 7.243E-03 |
| ENSG00000212743 | DKFZP667F0711 | mRNA | 2.251 | 9.897E-04 | 2.211 | 5.513E-03 |
| ENSG00000212993 | POU5F1B | mRNA | -2.029 | 3.190E-12 | -1.418 | 1.108E-04 |
| ENSG00000213186 | TRIM59 | mRNA | 2.050 | 3.311E-12 | 1.339 | 1.335E-03 |
| ENSG00000213563 | C8orf82 | mRNA | -2.347 | 4.244E-18 | -1.052 | 4.059E-03 |
| ENSG00000213578 | CPLX3 | mRNA | -3.079 | 1.245E-04 | -2.954 | 3.158E-04 |
| ENSG00000213972 | RP3-522P13.2 | mRNA | 1.500 | 1.435E-04 | 1.780 | 3.348E-05 |
| ENSG00000214212 | C19orf38 | mRNA | 1.559 | 1.587E-05 | 1.759 | 1.059E-04 |
| ENSG00000214252 | AZGP1P2 | mRNA | -4.715 | 1.238E-24 | -2.742 | 4.024E-08 |
| ENSG00000214274 | ANG | mRNA | -1.674 | 4.696E-08 | -1.658 | 7.524E-07 |
| ENSG00000214313 | AZGP1P1 | mRNA | -6.570 | 1.690E-41 | -4.783 | 4.892E-14 |
| ENSG00000214456 | PLIN5 | mRNA | -3.278 | 9.666E-29 | -2.842 | 5.638E-15 |
| ENSG00000214530 | STARD10 | mRNA | -1.704 | 1.374E-10 | -1.225 | 3.052E-05 |
| ENSG00000214711 | CAPN14 | mRNA | 2.440 | 3.152E-04 | 2.449 | 1.344E-05 |
| ENSG00000214787 | MS4A4E | mRNA | 1.046 | 9.024E-06 | 1.576 | 2.585E-09 |
| ENSG00000215018 | COL28A1 | mRNA | -2.421 | 1.434E-11 | -3.098 | 4.711E-17 |
| ENSG00000215183 | MSMP | mRNA | -2.235 | 7.132E-03 | -2.349 | 5.128E-03 |
| ENSG00000215298 | FP15737 | mRNA | -1.521 | 3.528E-12 | -1.195 | 2.077E-05 |
| ENSG00000215529 | EFCAB8 | mRNA | -1.844 | 3.552E-04 | -1.935 | 7.206E-05 |
| ENSG00000215845 | TSTD1 | mRNA | -1.255 | 2.598E-06 | -1.072 | 4.584E-05 |
| ENSG00000216490 | IFI30 | mRNA | 3.046 | 2.280E-07 | 2.974 | 2.494E-06 |
| ENSG00000217555 | CKLF | mRNA | 2.007 | 1.445E-08 | 1.613 | 6.125E-04 |
| ENSG00000218336 | TENM3 | mRNA | 1.039 | 1.471E-05 | 1.326 | 1.716E-05 |
| ENSG00000221510 | MIR548O | mRNA | 2.299 | 4.490E-04 | 2.878 | 2.330E-04 |
| ENSG00000221852 | KRTAP1-5 | mRNA | 4.237 | 5.040E-06 | 5.142 | 2.474E-10 |
| ENSG00000221857 | CTD-2527I21.4 | mRNA | -2.484 | 3.449E-09 | -2.705 | 3.177E-06 |
| ENSG00000221866 | PLXNA4 | mRNA | -1.323 | 2.380E-07 | -1.430 | 7.627E-06 |
| ENSG00000221953 | C1orf229 | mRNA | -1.313 | 2.219E-04 | -1.260 | 5.063E-04 |
| ENSG00000222033 | LINC01124 | mRNA | -3.495 | 2.192E-15 | -1.914 | 1.248E-05 |
| ENSG00000222040 | ADRA2B | mRNA | -3.670 | 3.649E-18 | -3.073 | 2.766E-08 |
| ENSG00000224383 | C17orf72 | mRNA | -2.119 | 6.638E-04 | -1.841 | 3.531E-03 |
| ENSG00000224957 | AC090044.1 | mRNA | -1.053 | 6.356E-03 | -1.357 | 3.365E-04 |
| ENSG00000225556 | C2CD4D | mRNA | -3.135 | 2.352E-20 | -2.288 | 2.229E-08 |
| ENSG00000226321 | AC104809.3 | mRNA | -2.313 | 2.754E-04 | -2.233 | 3.712E-04 |
| ENSG00000227191 | TRGC2 | mRNA | 1.259 | 2.783E-04 | 1.796 | 1.307E-05 |
| ENSG00000227835 | CARM1P1 | mRNA | -5.884 | 7.128E-15 | -5.759 | 1.928E-12 |
| ENSG00000227921 | AL353791.1 | mRNA | 1.666 | 1.038E-05 | 1.370 | 1.188E-03 |
| ENSG00000228835 | AC012123.1 | mRNA | -2.321 | 5.243E-08 | -2.822 | 5.631E-08 |
| ENSG00000229183 | PGA4 | mRNA | 2.444 | 7.859E-03 | 3.707 | 1.844E-05 |
| ENSG00000229544 | NKX1-2 | mRNA | 1.371 | 5.465E-03 | 2.091 | 4.320E-05 |
| ENSG00000229754 | CXCR2P1 | mRNA | 4.032 | 1.101E-06 | 3.691 | 8.397E-05 |
| ENSG00000229859 | PGA3 | mRNA | 2.351 | 1.903E-02 | 3.952 | 3.861E-04 |
| ENSG00000229961 | RP11-71G12.1 | mRNA | 1.026 | 2.147E-02 | 3.019 | 2.544E-06 |
| ENSG00000230657 | PRB4 | mRNA | -10.113 | 5.260E-16 | -6.700 | 9.847E-10 |
| ENSG00000231389 | HLA-DPA1 | mRNA | 1.585 | 3.066E-09 | 1.090 | 7.094E-04 |
| ENSG00000231486 | AC096579.7 | mRNA | 1.119 | 1.716E-03 | 1.751 | 3.510E-04 |
| ENSG00000231561 | CEACAMP5 | mRNA | -2.509 | 3.180E-09 | -1.327 | 2.891E-03 |
| ENSG00000231584 | FAHD2CP | mRNA | -1.830 | 8.765E-16 | -1.549 | 7.897E-09 |
| ENSG00000232216 | IGHV3-43 | mRNA | 1.450 | 3.251E-03 | 1.496 | 5.569E-03 |
| ENSG00000232706 | NUTM2HP | mRNA | -1.462 | 5.575E-05 | -1.283 | 3.059E-03 |
| ENSG00000232810 | TNF | mRNA | 2.122 | 1.058E-07 | 1.463 | 1.066E-03 |
| ENSG00000233224 | HIST1H2AM | mRNA | 2.335 | 7.150E-12 | 1.694 | 3.443E-06 |
| ENSG00000233256 | RP11-445K13.2 | mRNA | -2.974 | 4.617E-23 | -2.380 | 3.382E-10 |
| ENSG00000233487 | AC011322.1 | mRNA | -2.251 | 1.224E-07 | -1.786 | 1.045E-04 |
| ENSG00000233493 | TMEM238 | mRNA | -2.699 | 2.107E-14 | -1.505 | 8.450E-04 |
| ENSG00000234224 | TMEM229A | mRNA | -8.434 | 2.334E-24 | -6.121 | 5.922E-17 |
| ENSG00000234840 | RP11-399D6.2 | mRNA | -3.597 | 8.955E-10 | -3.639 | 1.505E-08 |
| ENSG00000234906 | APOC2 | mRNA | 1.945 | 2.878E-03 | 1.959 | 6.569E-03 |
| ENSG00000234949 | AC104667.3 | mRNA | -1.408 | 6.886E-03 | -1.670 | 8.106E-03 |
| ENSG00000235169 | SMIM1 | mRNA | -1.787 | 5.259E-14 | -1.065 | 2.821E-04 |
| ENSG00000235491 | AC097499.1 | mRNA | 3.049 | 1.234E-12 | 1.367 | 1.396E-03 |
| ENSG00000235568 | NFAM1 | mRNA | 1.399 | 4.814E-06 | 2.142 | 2.519E-10 |
| ENSG00000236055 | RP11-423O2.2 | mRNA | 1.166 | 5.167E-04 | 1.268 | 1.652E-03 |
| ENSG00000236882 | C5orf27 | mRNA | -2.995 | 3.402E-11 | -3.105 | 3.305E-09 |
| ENSG00000237276 | ANO7P1 | mRNA | -1.261 | 2.500E-05 | -1.018 | 1.389E-03 |
| ENSG00000237649 | KIFC1 | mRNA | 1.386 | 6.377E-04 | 1.385 | 3.882E-04 |
| ENSG00000238098 | ABCA17P | mRNA | -2.784 | 5.102E-07 | -2.426 | 1.815E-05 |
| ENSG00000239998 | LILRA2 | mRNA | 2.062 | 5.934E-08 | 2.164 | 3.280E-06 |
| ENSG00000240040 | AC096579.13 | mRNA | 1.154 | 7.396E-04 | 2.030 | 2.580E-04 |
| ENSG00000240602 | RP11-64D22.2 | mRNA | 2.113 | 1.311E-09 | 1.791 | 3.576E-04 |
| ENSG00000241635 | UGT1A8 | mRNA | 1.685 | 2.200E-09 | 1.506 | 2.179E-04 |
| ENSG00000241717 | VWFP1 | mRNA | -5.347 | 1.248E-08 | -5.182 | 6.902E-08 |
| ENSG00000241794 | SPRR2A | mRNA | 7.265 | 1.061E-11 | 6.354 | 9.725E-09 |
| ENSG00000242252 | BGLAP | mRNA | -3.986 | 1.789E-10 | -2.481 | 1.704E-04 |
| ENSG00000242515 | UGT1A10 | mRNA | 2.045 | 5.552E-07 | 1.167 | 4.879E-03 |
| ENSG00000242550 | SERPINB10 | mRNA | 3.829 | 4.986E-04 | 4.577 | 6.640E-06 |
| ENSG00000242612 | DECR2 | mRNA | -1.895 | 2.723E-15 | -1.104 | 5.562E-04 |
| ENSG00000242770 | RP11-180K7.1 | mRNA | 2.628 | 2.370E-07 | 2.947 | 1.882E-07 |
| ENSG00000242952 | RP11-187O7.1 | mRNA | 1.875 | 8.024E-03 | 2.725 | 1.098E-07 |
| ENSG00000243137 | PSG4 | mRNA | 4.077 | 6.991E-03 | 5.010 | 1.586E-03 |
| ENSG00000243509 | TNFRSF6B | mRNA | 1.809 | 5.381E-05 | 1.964 | 6.024E-05 |
| ENSG00000243927 | MRPS6 | mRNA | 2.053 | 1.792E-15 | 1.902 | 2.602E-08 |
| ENSG00000244122 | UGT1A7 | mRNA | 2.171 | 5.379E-08 | 1.982 | 1.982E-05 |
| ENSG00000244474 | UGT1A4 | mRNA | 1.271 | 7.652E-03 | 1.741 | 6.281E-04 |
| ENSG00000244476 | ERVFRD-1 | mRNA | 3.114 | 1.124E-07 | 2.590 | 1.618E-03 |
| ENSG00000244482 | LILRA6 | mRNA | 3.305 | 2.682E-11 | 3.250 | 7.797E-09 |
| ENSG00000244682 | FCGR2C | mRNA | 1.606 | 9.322E-07 | 2.082 | 3.195E-10 |
| ENSG00000244694 | PTCHD4 | mRNA | 2.158 | 4.030E-11 | 2.337 | 2.390E-10 |
| ENSG00000248477 | RP11-848G14.2 | mRNA | 1.800 | 8.776E-13 | 1.516 | 8.670E-09 |
| ENSG00000248727 | CTC-236F12.4 | mRNA | -1.220 | 4.168E-05 | -1.071 | 5.126E-04 |
| ENSG00000249034 | AC005609.1 | mRNA | -2.191 | 5.507E-03 | -2.015 | 7.023E-03 |
| ENSG00000249437 | NAIP | mRNA | 1.591 | 2.288E-12 | 1.339 | 7.489E-07 |
| ENSG00000249631 | RP11-281P23.2 | mRNA | 1.780 | 3.050E-07 | 1.601 | 5.594E-06 |
| ENSG00000249763 | RP11-618I10.1 | mRNA | 2.990 | 3.833E-03 | 3.665 | 1.966E-04 |
| ENSG00000250026 | RP11-646E20.6 | mRNA | 3.030 | 4.179E-04 | 3.169 | 1.980E-03 |
| ENSG00000250423 | KIAA1210 | mRNA | -1.909 | 6.709E-05 | -1.995 | 7.924E-05 |
| ENSG00000250565 | ATP6V1E2 | mRNA | -1.159 | 1.694E-06 | -1.003 | 3.246E-04 |
| ENSG00000251158 | RP11-98J23.1 | mRNA | 1.705 | 3.472E-05 | 1.797 | 2.817E-05 |
| ENSG00000251655 | PRB1 | mRNA | -4.157 | 5.821E-13 | -2.225 | 7.151E-04 |
| ENSG00000251691 | RP11-618I10.4 | mRNA | 1.192 | 1.158E-02 | 1.212 | 4.586E-03 |
| ENSG00000253193 | FCGR1C | mRNA | 3.761 | 8.191E-08 | 2.762 | 4.239E-03 |
| ENSG00000254415 | SIGLEC14 | mRNA | 2.501 | 3.157E-03 | 3.057 | 2.755E-05 |
| ENSG00000254521 | SIGLEC12 | mRNA | 3.027 | 1.927E-06 | 2.396 | 4.551E-04 |
| ENSG00000254636 | ARMS2 | mRNA | -2.504 | 1.703E-12 | -1.550 | 2.206E-05 |
| ENSG00000254681 | PKD1P5 | mRNA | -1.694 | 3.121E-07 | -1.053 | 5.785E-03 |
| ENSG00000254967 | RP11-680F20.6 | mRNA | -2.192 | 7.493E-04 | -2.938 | 9.477E-05 |
| ENSG00000255154 | RPP14 | mRNA | 4.046 | 1.821E-05 | 3.176 | 7.446E-03 |
| ENSG00000255346 | NOX5 | mRNA | -2.249 | 1.569E-07 | -2.146 | 1.519E-07 |
| ENSG00000255769 | RP11-152F13.3 | mRNA | -2.094 | 3.304E-09 | -1.549 | 2.894E-04 |
| ENSG00000256018 | HIST1H3G | mRNA | 2.482 | 1.254E-06 | 2.401 | 1.778E-06 |
| ENSG00000256316 | HIST1H3F | mRNA | 2.439 | 9.479E-09 | 1.820 | 2.039E-05 |
| ENSG00000256391 | SDIM1 | mRNA | 3.342 | 3.495E-06 | 3.295 | 1.854E-05 |
| ENSG00000256515 | CCL3L3 | mRNA | 1.902 | 1.361E-04 | 2.942 | 8.860E-07 |
| ENSG00000256660 | CLEC12B | mRNA | 2.641 | 2.681E-08 | 2.301 | 1.600E-04 |
| ENSG00000256812 | CAPNS2 | mRNA | 1.023 | 1.430E-02 | 1.365 | 5.004E-04 |
| ENSG00000257017 | HP | mRNA | -6.025 | 3.357E-31 | -5.198 | 5.344E-15 |
| ENSG00000257108 | NHLRC4 | mRNA | -2.119 | 1.449E-04 | -1.803 | 1.632E-03 |
| ENSG00000257335 | MGAM | mRNA | 2.156 | 4.800E-09 | 1.437 | 1.236E-04 |
| ENSG00000257743 | RP11-1220K2.2 | mRNA | -4.178 | 4.769E-14 | -2.835 | 7.421E-06 |
| ENSG00000258072 | RP11-554D14.2 | mRNA | 2.100 | 1.210E-05 | 1.859 | 2.527E-03 |
| ENSG00000258474 | RP11-187E13.1 | mRNA | -1.140 | 2.453E-02 | -1.863 | 2.633E-03 |
| ENSG00000258572 | RP11-1070N10.3 | mRNA | 1.825 | 1.540E-03 | 2.443 | 2.928E-06 |
| ENSG00000258708 | SLC25A21-AS1 | mRNA | -1.367 | 1.024E-04 | -1.526 | 1.348E-04 |
| ENSG00000258818 | RNASE4 | mRNA | -1.369 | 1.385E-06 | -1.393 | 1.226E-06 |
| ENSG00000258867 | LINC01146 | mRNA | 3.119 | 1.589E-15 | 2.979 | 4.144E-09 |
| ENSG00000258947 | TUBB3 | mRNA | 1.778 | 4.411E-05 | 1.928 | 8.680E-06 |
| ENSG00000259171 | AL163636.6 | mRNA | -1.931 | 4.648E-09 | -1.636 | 1.486E-05 |
| ENSG00000259472 | RP13-996F3.3 | mRNA | -2.163 | 4.407E-09 | -1.582 | 2.931E-04 |
| ENSG00000260908 | CTB-134H23.3 | mRNA | -2.388 | 9.107E-06 | -1.583 | 6.510E-03 |
| ENSG00000261161 | RP11-58A18.1 | mRNA | -3.852 | 8.688E-13 | -2.846 | 7.626E-06 |
| ENSG00000261427 | CTD-2349B8.1 | mRNA | 1.586 | 1.199E-11 | 1.027 | 3.405E-03 |
| ENSG00000261553 | RP11-29G8.3 | mRNA | 2.485 | 4.626E-03 | 2.917 | 2.733E-04 |
| ENSG00000261606 | RP11-414J4.2 | mRNA | -4.966 | 1.406E-09 | -4.327 | 3.584E-08 |
| ENSG00000261857 | MIA | mRNA | -2.381 | 3.530E-09 | -1.632 | 1.240E-03 |
| ENSG00000262660 | SLC25A10 | mRNA | -2.123 | 9.490E-08 | -1.719 | 4.611E-04 |
| ENSG00000263846 | CIAPIN1P | mRNA | -1.884 | 5.747E-03 | -3.429 | 1.836E-05 |
| ENSG00000264527 | WI2-1959D15.1 | mRNA | -2.548 | 5.893E-05 | -1.880 | 3.026E-03 |
| ENSG00000266964 | FXYD1 | mRNA | -1.933 | 3.629E-07 | -1.594 | 6.628E-05 |
| ENSG00000267097 | RP11-309E23.2 | mRNA | 1.017 | 8.241E-05 | 1.269 | 3.062E-05 |
| ENSG00000267259 | CTD-2008P7.9 | mRNA | -2.483 | 3.316E-09 | -2.693 | 7.944E-09 |
| ENSG00000267795 | SMIM22 | mRNA | -1.191 | 1.929E-03 | -1.439 | 1.845E-05 |
| ENSG00000268194 | LINC01101 | mRNA | -3.972 | 1.633E-10 | -4.439 | 8.122E-13 |
| ENSG00000268403 | AC132192.1 | mRNA | -2.188 | 3.883E-03 | -1.992 | 8.626E-03 |
| ENSG00000268500 | SIGLEC5 | mRNA | 2.077 | 4.752E-05 | 1.848 | 1.102E-03 |
| ENSG00000268628 | AL121761.2 | mRNA | 1.468 | 2.583E-03 | 2.714 | 1.479E-08 |
| ENSG00000268758 | EMR4P | mRNA | 1.467 | 6.793E-04 | 2.551 | 1.871E-09 |
| ENSG00000268849 | SIGLEC22P | mRNA | 2.041 | 5.786E-05 | 2.126 | 4.130E-04 |
| ENSG00000269035 | CTD-2521M24.10 | mRNA | -3.436 | 1.214E-06 | -2.424 | 7.737E-04 |
| ENSG00000269071 | AC138517.1 | mRNA | -3.532 | 4.492E-11 | -2.962 | 2.859E-08 |
| ENSG00000269113 | TRABD2B | mRNA | -1.126 | 2.611E-04 | -1.024 | 1.876E-03 |
| ENSG00000270087 | RP11-399K21.11 | mRNA | -1.492 | 5.050E-08 | -1.034 | 8.630E-03 |
| ENSG00000270386 | UGT2A1 | mRNA | 1.449 | 2.133E-06 | 1.701 | 4.977E-06 |
| ENSG00000270547 | RP11-536O18.2 | mRNA | 3.071 | 1.356E-08 | 3.850 | 1.568E-10 |
| ENSG00000270757 | HSPE1-MOB4 | mRNA | 1.355 | 3.755E-05 | 1.217 | 2.788E-03 |
| ENSG00000271225 | RP11-460N11.3 | mRNA | -1.237 | 1.466E-03 | -1.213 | 2.887E-03 |
| ENSG00000271271 | UGT2A2 | mRNA | 1.187 | 1.472E-03 | 1.587 | 1.482E-03 |
| ENSG00000271605 | MILR1 | mRNA | 1.217 | 1.512E-04 | 1.124 | 2.066E-03 |
| ENSG00000271723 | MROH7-TTC4 | mRNA | -1.326 | 2.332E-09 | -1.034 | 3.131E-05 |
| ENSG00000272908 | RP11-121A8.1 | mRNA | 1.921 | 2.785E-09 | 1.319 | 1.887E-03 |
| ENSG00000272962 | SLC5A3 | mRNA | 2.765 | 4.343E-07 | 3.025 | 5.875E-06 |
| ENSG00000273259 | RP11-986E7.7 | mRNA | -4.336 | 7.053E-13 | -2.737 | 8.618E-06 |
| XLOC_008308 | XLOC_008308 | mRNA | 2.609 | 2.678E-03 | 5.303 | 7.499E-13 |
| XLOC_019295 | XLOC_019295 | mRNA | 1.116 | 3.900E-03 | 1.910 | 4.395E-07 |
| XLOC_090569 | XLOC_090569 | mRNA | -3.160 | 1.030E-09 | -2.046 | 6.042E-04 |
| XLOC_095236 | XLOC_095236 | mRNA | 3.522 | 2.863E-08 | 2.971 | 2.041E-04 |
| XLOC_095324 | XLOC_095324 | mRNA | -3.953 | 5.888E-07 | -4.211 | 3.935E-07 |
| XLOC_095369 | XLOC_095369 | mRNA | 1.178 | 1.665E-04 | 1.052 | 1.022E-03 |
| XLOC_095409 | XLOC_095409 | mRNA | 1.725 | 3.703E-05 | 1.026 | 7.565E-03 |
| XLOC_095439 | XLOC_095439 | mRNA | 2.628 | 1.380E-08 | 1.999 | 2.666E-05 |
| XLOC_095443 | XLOC_095443 | mRNA | 1.282 | 6.742E-03 | 1.572 | 8.915E-04 |
| XLOC_095468 | XLOC_095468 | mRNA | -1.715 | 3.336E-09 | -1.006 | 2.298E-05 |
| XLOC_095508 | XLOC_095508 | mRNA | 2.525 | 1.284E-07 | 2.305 | 6.816E-04 |
| XLOC_095517 | XLOC_095517 | mRNA | 2.968 | 8.664E-08 | 2.216 | 6.143E-03 |
| XLOC_095567 | XLOC_095567 | mRNA | 1.616 | 9.368E-06 | 1.792 | 6.851E-06 |
| ENST00000309874 | RP11-23P13.6-001 | lncRNA | -2.526 | 3.954E-09 | -2.066 | 2.105E-04 |
| ENST00000331944 | LINC00152-003 | lncRNA | 2.078 | 2.172E-07 | 1.967 | 1.026E-05 |
| ENST00000342963 | AC093620.5-001 | lncRNA | -3.218 | 7.588E-11 | -2.309 | 3.932E-05 |
| ENST00000369936 | KIAA1324-001 | lncRNA | -2.991 | 2.980E-13 | -3.000 | 6.137E-14 |
| ENST00000371162 | MIR4435-1HG-021 | lncRNA | 2.884 | 9.246E-13 | 3.054 | 1.643E-11 |
| ENST00000409054 | LINC00152-005 | lncRNA | 2.339 | 8.261E-07 | 2.392 | 4.453E-06 |
| ENST00000409139 | LINC00152-007 | lncRNA | 2.584 | 1.029E-05 | 2.462 | 2.346E-04 |
| ENST00000409569 | MIR4435-1HG-001 | lncRNA | 1.875 | 3.662E-07 | 1.633 | 1.699E-04 |
| ENST00000416624 | CD69-002 | lncRNA | 3.337 | 1.926E-05 | 3.403 | 8.812E-05 |
| ENST00000417917 | RP4-758J18.10-002 | lncRNA | -1.906 | 5.365E-06 | -1.844 | 2.660E-04 |
| ENST00000420431 | CTD-2020K17.4-003 | lncRNA | -2.478 | 1.419E-08 | -2.351 | 8.808E-07 |
| ENST00000423255 | RP11-154D6.1-006 | lncRNA | -2.991 | 1.564E-04 | -3.058 | 4.349E-04 |
| ENST00000429156 | MIR205HG-002 | lncRNA | 3.028 | 2.586E-10 | 1.947 | 3.438E-04 |
| ENST00000432473 | Z83851.1-003 | lncRNA | -1.768 | 1.227E-05 | -2.005 | 1.393E-05 |
| ENST00000432601 | LINC00969-010 | lncRNA | -2.141 | 8.878E-04 | -2.276 | 5.567E-04 |
| ENST00000439362 | MIR4435-1HG-004 | lncRNA | 3.235 | 3.925E-08 | 3.310 | 5.784E-05 |
| ENST00000441379 | ITGB2-AS1-001 | lncRNA | 1.298 | 3.668E-04 | 1.579 | 2.710E-04 |
| ENST00000444125 | RP11-65J3.1-003 | lncRNA | 3.309 | 2.145E-07 | 3.001 | 1.784E-05 |
| ENST00000452402 | RP11-250B2.3-001 | lncRNA | -1.687 | 1.097E-03 | -2.526 | 2.638E-05 |
| ENST00000456944 | RP11-82L18.2-002 | lncRNA | -2.735 | 4.703E-07 | -2.362 | 3.843E-05 |
| ENST00000458624 | AC007009.1-001 | lncRNA | -2.987 | 1.547E-06 | -3.233 | 7.832E-07 |
| ENST00000459788 | HDLBP-046 | lncRNA | -3.046 | 2.701E-09 | -1.721 | 4.183E-04 |
| ENST00000460209 | IGFBP3-017 | lncRNA | 2.138 | 5.353E-05 | 2.010 | 4.179E-04 |
| ENST00000460477 | IGFBP3-016 | lncRNA | 3.385 | 1.309E-12 | 2.769 | 2.564E-06 |
| ENST00000461325 | STAB1-003 | lncRNA | 1.413 | 2.898E-03 | 2.197 | 1.515E-05 |
| ENST00000461686 | CBS-005 | lncRNA | -2.402 | 2.063E-05 | -2.721 | 5.320E-05 |
| ENST00000462363 | PTPRC-007 | lncRNA | 2.786 | 1.128E-07 | 2.299 | 5.434E-05 |
| ENST00000462667 | LTF-009 | lncRNA | -3.473 | 8.789E-06 | -3.298 | 2.877E-04 |
| ENST00000463990 | IL1RL1-006 | lncRNA | 2.385 | 4.812E-04 | 4.014 | 1.687E-06 |
| ENST00000467401 | WNK2-008 | lncRNA | -1.980 | 4.607E-08 | -1.873 | 1.297E-06 |
| ENST00000467654 | FCGR2A-007 | lncRNA | 2.570 | 1.717E-08 | 2.622 | 6.858E-06 |
| ENST00000469532 | RAC2-006 | lncRNA | 2.012 | 7.628E-08 | 1.850 | 1.573E-04 |
| ENST00000469630 | TBXAS1-015 | lncRNA | 2.259 | 1.566E-07 | 2.378 | 2.605E-07 |
| ENST00000473079 | EPHB3-003 | lncRNA | -2.062 | 4.118E-08 | -2.092 | 5.541E-06 |
| ENST00000473291 | HHLA1-002 | lncRNA | 2.341 | 1.028E-07 | 2.426 | 2.581E-05 |
| ENST00000474788 | PLEK-002 | lncRNA | 1.646 | 2.273E-04 | 2.797 | 1.013E-06 |
| ENST00000475389 | LAIR1-007 | lncRNA | 1.673 | 5.850E-04 | 1.813 | 1.189E-04 |
| ENST00000476245 | ALDH1L1-011 | lncRNA | -2.649 | 6.233E-13 | -2.103 | 4.027E-04 |
| ENST00000477251 | AZGP1-004 | lncRNA | -4.865 | 5.254E-14 | -3.970 | 8.694E-08 |
| ENST00000480308 | FCGR2B-003 | lncRNA | 1.846 | 6.923E-07 | 2.059 | 2.029E-06 |
| ENST00000482720 | XBP1-005 | lncRNA | -2.101 | 5.662E-08 | -1.739 | 1.701E-05 |
| ENST00000483977 | MRPS6-002 | lncRNA | 2.221 | 4.309E-07 | 2.007 | 1.850E-04 |
| ENST00000484724 | ALDH1L1-010 | lncRNA | -2.724 | 2.796E-09 | -2.349 | 4.406E-05 |
| ENST00000485778 | FCGR2B-004 | lncRNA | 2.333 | 5.481E-06 | 2.361 | 4.884E-05 |
| ENST00000485887 | ADCY3-008 | lncRNA | 1.481 | 6.945E-06 | 1.830 | 3.866E-06 |
| ENST00000486394 | SLC5A1-003 | lncRNA | -4.777 | 2.352E-09 | -3.845 | 9.132E-07 |
| ENST00000487866 | ECHDC2-008 | lncRNA | -2.402 | 1.583E-06 | -1.696 | 6.205E-04 |
| ENST00000489551 | CSF3R-008 | lncRNA | 2.298 | 6.866E-05 | 2.407 | 3.856E-06 |
| ENST00000490302 | PKDCC-004 | lncRNA | -1.994 | 1.103E-03 | -2.230 | 1.482E-04 |
| ENST00000490800 | CLDN1-004 | lncRNA | 1.897 | 2.134E-05 | 2.493 | 7.833E-07 |
| ENST00000490939 | CMAHP-008 | lncRNA | -1.381 | 3.215E-04 | -1.650 | 4.175E-05 |
| ENST00000492037 | PPP1R1B-006 | lncRNA | -6.660 | 6.763E-21 | -5.000 | 1.742E-12 |
| ENST00000492560 | TBXAS1-012 | lncRNA | 1.921 | 2.683E-03 | 2.343 | 5.884E-05 |
| ENST00000492788 | CCL3L1-002 | lncRNA | 2.851 | 1.828E-03 | 3.254 | 5.962E-04 |
| ENST00000492816 | FN1-007 | lncRNA | 1.531 | 4.408E-04 | 2.553 | 1.006E-06 |
| ENST00000492861 | PKDCC-002 | lncRNA | -3.377 | 5.125E-07 | -3.661 | 1.144E-05 |
| ENST00000495765 | AZGP1-002 | lncRNA | -5.084 | 1.312E-09 | -5.362 | 7.698E-09 |
| ENST00000496542 | FN1-032 | lncRNA | 1.924 | 1.397E-03 | 2.925 | 9.551E-05 |
| ENST00000498496 | SERPINB4-003 | lncRNA | 4.507 | 2.640E-14 | 3.257 | 1.941E-05 |
| ENST00000504853 | CFI-002 | lncRNA | 2.105 | 3.572E-10 | 1.949 | 3.802E-06 |
| ENST00000506576 | STATH-008 | lncRNA | -6.600 | 1.226E-12 | -5.240 | 4.051E-05 |
| ENST00000506834 | HK3-002 | lncRNA | 2.180 | 1.408E-04 | 4.209 | 3.534E-11 |
| ENST00000507211 | STATH-004 | lncRNA | -7.618 | 3.133E-14 | -6.142 | 3.927E-07 |
| ENST00000507962 | STATH-006 | lncRNA | -6.502 | 3.528E-10 | -5.415 | 6.080E-06 |
| ENST00000510010 | STATH-005 | lncRNA | -6.592 | 2.934E-14 | -5.744 | 2.679E-07 |
| ENST00000514421 | CCL28-004 | lncRNA | -3.259 | 1.010E-04 | -3.627 | 6.623E-05 |
| ENST00000515433 | TGFBI-009 | lncRNA | 1.630 | 1.819E-05 | 2.151 | 4.703E-06 |
| ENST00000515734 | PART1-001 | lncRNA | -3.144 | 1.416E-08 | -3.324 | 5.476E-08 |
| ENST00000518033 | ITGB2-019 | lncRNA | 2.297 | 2.819E-03 | 3.629 | 3.357E-08 |
| ENST00000519594 | LCP2-010 | lncRNA | 1.320 | 6.730E-04 | 1.671 | 1.256E-04 |
| ENST00000519700 | RP11-180K7.1-003 | lncRNA | 2.069 | 5.948E-03 | 2.638 | 3.683E-04 |
| ENST00000521431 | PSEN2-011 | lncRNA | -1.247 | 7.720E-04 | -1.551 | 2.823E-04 |
| ENST00000522760 | LCP2-009 | lncRNA | 2.496 | 2.366E-07 | 2.804 | 1.477E-06 |
| ENST00000522902 | HAVCR2-002 | lncRNA | 2.397 | 2.725E-03 | 2.810 | 9.504E-06 |
| ENST00000523224 | SLA-008 | lncRNA | 1.469 | 7.488E-04 | 1.589 | 3.753E-04 |
| ENST00000525348 | CD44-017 | lncRNA | 2.533 | 1.966E-05 | 2.532 | 5.188E-05 |
| ENST00000525954 | FDFT1-010 | lncRNA | -1.511 | 1.096E-05 | -1.360 | 2.599E-04 |
| ENST00000526531 | EFCAB4A-013 | lncRNA | -2.764 | 2.600E-09 | -2.764 | 3.892E-04 |
| ENST00000527763 | EFCAB4A-004 | lncRNA | -3.289 | 2.749E-19 | -2.608 | 2.473E-08 |
| ENST00000528587 | MS4A7-006 | lncRNA | 2.962 | 1.326E-06 | 2.941 | 4.760E-05 |
| ENST00000528694 | EFCAB4A-005 | lncRNA | -3.315 | 4.372E-15 | -2.614 | 1.330E-07 |
| ENST00000528808 | MS4A7-003 | lncRNA | 2.142 | 1.037E-04 | 2.458 | 6.246E-05 |
| ENST00000529924 | IL10RA-006 | lncRNA | 1.925 | 2.021E-05 | 1.951 | 1.597E-05 |
| ENST00000530244 | CREB3L1-002 | lncRNA | -1.915 | 9.024E-06 | -1.566 | 6.021E-04 |
| ENST00000530336 | PYGL-004 | lncRNA | 2.314 | 1.131E-06 | 2.264 | 1.093E-05 |
| ENST00000530674 | SULF1-007 | lncRNA | 3.090 | 1.392E-08 | 3.123 | 5.426E-06 |
| ENST00000530688 | EFCAB4A-006 | lncRNA | -3.991 | 3.831E-09 | -2.086 | 4.173E-04 |
| ENST00000531141 | CD44-013 | lncRNA | 2.373 | 6.844E-07 | 2.157 | 6.851E-04 |
| ENST00000531914 | MS4A6A-019 | lncRNA | 2.684 | 7.916E-09 | 2.780 | 2.716E-06 |
| ENST00000532061 | RP11-532E4.2-001 | lncRNA | -3.873 | 8.239E-07 | -3.862 | 1.606E-06 |
| ENST00000532395 | HID1-005 | lncRNA | -2.777 | 7.365E-08 | -1.641 | 4.264E-05 |
| ENST00000533138 | FTH1-008 | lncRNA | 1.831 | 1.014E-05 | 1.987 | 4.149E-05 |
| ENST00000533780 | FCGR3B-006 | lncRNA | 3.356 | 5.433E-05 | 3.922 | 4.577E-06 |
| ENST00000533897 | CTSC-010 | lncRNA | 2.778 | 5.588E-08 | 2.595 | 3.697E-05 |
| ENST00000534294 | AMICA1-005 | lncRNA | 2.656 | 1.714E-05 | 2.927 | 3.359E-05 |
| ENST00000537044 | CD163-004 | lncRNA | 3.199 | 2.218E-06 | 3.681 | 1.084E-04 |
| ENST00000539179 | PRR4-007 | lncRNA | -5.948 | 4.936E-13 | -5.488 | 4.309E-12 |
| ENST00000548088 | KRT7-003 | lncRNA | -2.285 | 2.017E-06 | -2.096 | 3.116E-04 |
| ENST00000548900 | RP11-1143G9.4-001 | lncRNA | -4.503 | 2.967E-06 | -3.900 | 2.220E-04 |
| ENST00000549668 | NELL2-014 | lncRNA | -2.723 | 3.740E-03 | -4.350 | 3.393E-06 |
| ENST00000550042 | RP1-34H18.1-001 | lncRNA | -1.642 | 6.232E-04 | -1.938 | 4.287E-04 |
| ENST00000553862 | NDRG2-065 | lncRNA | -2.299 | 1.676E-08 | -2.180 | 2.020E-05 |
| ENST00000553883 | ASB2-004 | lncRNA | 1.782 | 1.061E-03 | 1.946 | 6.343E-04 |
| ENST00000555650 | NDRG2-074 | lncRNA | -2.830 | 6.504E-08 | -2.274 | 1.122E-04 |
| ENST00000556785 | EGLN3-013 | lncRNA | 3.458 | 8.764E-07 | 3.457 | 4.759E-04 |
| ENST00000557416 | NDRG2-073 | lncRNA | -2.365 | 3.273E-04 | -3.007 | 1.975E-05 |
| ENST00000558761 | HDC-002 | lncRNA | 2.483 | 3.202E-06 | 2.775 | 8.277E-09 |
| ENST00000558769 | DNAJC17-008 | lncRNA | -1.740 | 4.509E-04 | -1.723 | 4.738E-04 |
| ENST00000559816 | HDC-005 | lncRNA | 1.978 | 1.243E-05 | 2.067 | 4.989E-05 |
| ENST00000560590 | RP11-307C19.2-002 | lncRNA | -1.423 | 1.040E-03 | -1.947 | 7.727E-05 |
| ENST00000561838 | ITGAM-005 | lncRNA | 2.218 | 2.708E-03 | 2.676 | 3.520E-04 |
| ENST00000562735 | RAB26-002 | lncRNA | -2.355 | 4.609E-12 | -1.817 | 1.716E-05 |
| ENST00000563192 | RP11-304L19.5-001 | lncRNA | -1.303 | 1.463E-03 | -1.763 | 5.238E-05 |
| ENST00000563863 | RP11-161M6.2-003 | lncRNA | -3.959 | 1.879E-18 | -3.458 | 8.893E-08 |
| ENST00000566746 | CES3-006 | lncRNA | -3.119 | 5.246E-08 | -3.685 | 3.934E-08 |
| ENST00000567833 | PRSS8-002 | lncRNA | -2.258 | 4.181E-08 | -1.823 | 3.111E-04 |
| ENST00000568164 | LDHD-004 | lncRNA | -2.675 | 5.161E-07 | -2.707 | 2.631E-05 |
| ENST00000568370 | MT1X-003 | lncRNA | -3.002 | 4.007E-10 | -2.768 | 9.509E-07 |
| ENST00000568930 | ADCY7-011 | lncRNA | 1.351 | 1.571E-03 | 1.747 | 3.169E-04 |
| ENST00000569193 | GPT2-003 | lncRNA | -2.693 | 2.800E-12 | -1.833 | 7.062E-05 |
| ENST00000569739 | NOXO1-007 | lncRNA | -3.847 | 3.946E-12 | -2.782 | 2.547E-06 |
| ENST00000570977 | TOM1L1-017 | lncRNA | -3.757 | 1.080E-09 | -2.472 | 1.430E-04 |
| ENST00000572176 | ATP2A3-011 | lncRNA | -2.387 | 6.172E-07 | -1.720 | 1.464E-04 |
| ENST00000572694 | ATP2A3-014 | lncRNA | -2.833 | 3.311E-12 | -2.021 | 2.267E-05 |
| ENST00000573019 | ZG16B-004 | lncRNA | -4.995 | 1.811E-11 | -5.838 | 2.757E-12 |
| ENST00000574999 | ATP2A3-010 | lncRNA | -3.209 | 1.549E-08 | -2.288 | 5.412E-05 |
| ENST00000576423 | ZG16B-003 | lncRNA | -5.530 | 5.822E-14 | -4.864 | 7.496E-07 |
| ENST00000580601 | SAP30BP-009 | lncRNA | -1.860 | 3.424E-08 | -1.349 | 2.959E-04 |
| ENST00000583810 | PIK3R5-012 | lncRNA | 1.363 | 1.630E-03 | 1.869 | 1.164E-05 |
| ENST00000587694 | RAMP2-AS1-003 | lncRNA | -1.437 | 3.008E-03 | -2.038 | 6.015E-06 |
| ENST00000588439 | TYROBP-004 | lncRNA | 2.301 | 1.291E-04 | 2.254 | 9.048E-05 |
| ENST00000588817 | CCL3L3-002 | lncRNA | 2.326 | 2.834E-04 | 2.408 | 1.481E-04 |
| ENST00000589728 | PLIN5-002 | lncRNA | -3.923 | 1.501E-13 | -2.891 | 3.565E-08 |
| ENST00000590761 | CCL4L2-004 | lncRNA | 2.971 | 2.261E-05 | 3.776 | 1.624E-06 |
| ENST00000592016 | CTD-2008P7.9-001 | lncRNA | -1.584 | 4.658E-03 | -2.618 | 2.561E-06 |
| ENST00000595325 | MYO1F-002 | lncRNA | 3.030 | 4.216E-12 | 2.993 | 2.668E-09 |
| ENST00000595692 | CAMSAP3-003 | lncRNA | -2.943 | 2.438E-11 | -1.926 | 1.687E-05 |
| ENST00000596580 | FARP1-017 | lncRNA | -1.392 | 2.029E-04 | -1.589 | 3.418E-04 |
| ENST00000597184 | KCNN4-012 | lncRNA | -5.294 | 1.236E-17 | -4.928 | 1.427E-12 |
| ENST00000600463 | IFI30-002 | lncRNA | 1.798 | 2.683E-06 | 1.658 | 1.412E-04 |
| ENST00000601549 | KCNN4-011 | lncRNA | -4.283 | 7.208E-09 | -4.041 | 1.285E-07 |
| ENST00000601876 | PLAUR-005 | lncRNA | 2.238 | 1.577E-03 | 3.193 | 8.303E-05 |
| ENST00000603052 | RP11-803D5.4-001 | lncRNA | 1.913 | 7.441E-06 | 1.939 | 4.664E-05 |
| ENST00000603827 | MIR4435-1HG-019 | lncRNA | 1.404 | 4.577E-04 | 1.664 | 6.893E-05 |
| ENST00000605039 | BIN2-003 | lncRNA | 2.159 | 1.579E-05 | 2.183 | 7.643E-05 |
| ENST00000606712 | DSE-006 | lncRNA | 3.217 | 5.620E-09 | 3.005 | 1.644E-04 |
| ENST00000607094 | DSE-004 | lncRNA | 1.918 | 3.550E-07 | 1.683 | 6.137E-04 |
| ENST00000608346 | RP11-299J3.8-004 | lncRNA | -2.235 | 1.050E-06 | -1.917 | 8.048E-05 |
| TCONS_00009832 | FCGR2B-OT1 | lncRNA | 1.996 | 5.491E-08 | 2.307 | 2.901E-08 |
| TCONS_00030554 | SRGN-OT1 | lncRNA | 3.252 | 9.543E-06 | 3.451 | 3.459E-04 |
| TCONS_00033229 | LINC151 | lncRNA | -3.998 | 2.076E-07 | -3.176 | 2.498E-04 |
| TCONS_00033280 | DMBT1-OT1 | lncRNA | -4.929 | 9.667E-11 | -3.767 | 6.573E-06 |
| TCONS_00035869 | C10orf128-OT1 | lncRNA | 1.593 | 1.318E-04 | 1.927 | 1.962E-04 |
| TCONS_00042269 | CREB3L1-OT1 | lncRNA | -2.567 | 4.668E-07 | -2.210 | 4.153E-05 |
| TCONS_00053380 | SHANK2-OT2 | lncRNA | -2.756 | 8.103E-13 | -1.845 | 2.175E-05 |
| TCONS_00104469 | CCL22-OT1 | lncRNA | 2.855 | 1.852E-05 | 2.286 | 6.208E-04 |
| TCONS_00135544 | SERPINB3-OT1 | lncRNA | 4.542 | 8.117E-17 | 3.457 | 9.767E-06 |
| TCONS_00135545 | SERPINB3-OT2 | lncRNA | 3.970 | 3.584E-14 | 2.975 | 3.534E-05 |
| TCONS_00155957 | ARHGAP25-OT1 | lncRNA | 2.434 | 1.651E-05 | 2.507 | 1.859E-04 |
| TCONS_00161244 | C2orf88-OT1 | lncRNA | -3.388 | 6.872E-07 | -3.722 | 3.987E-06 |
| TCONS_00176846 | HCK-OT1 | lncRNA | 2.374 | 2.851E-05 | 2.679 | 4.116E-06 |
| TCONS_00216101 | LINC676 | lncRNA | 2.218 | 4.814E-06 | 2.230 | 4.039E-04 |
| TCONS_00216102 | LINC677 | lncRNA | 2.309 | 4.368E-06 | 2.561 | 1.157E-05 |
| TCONS_00216153 | LINC681 | lncRNA | 2.700 | 4.793E-06 | 2.489 | 3.170E-04 |
| TCONS_00218234 | SLC2A9-OT1 | lncRNA | -1.561 | 4.307E-04 | -2.115 | 4.774E-04 |
| TCONS_00218306 | HS3ST1-OT1 | lncRNA | 1.707 | 1.664E-07 | 1.589 | 1.336E-05 |
| TCONS_00223508 | HPGD-OT1 | lncRNA | 2.581 | 3.944E-04 | 2.561 | 5.427E-04 |
| TCONS_00230357 | FAM196B-AS1 | lncRNA | 2.051 | 2.395E-06 | 1.837 | 3.617E-04 |
| TCONS_00237199 | LCP2-OT1 | lncRNA | 3.284 | 2.912E-08 | 3.703 | 4.460E-07 |
| TCONS_00246524 | FAM65B-OT1 | lncRNA | 2.808 | 3.849E-07 | 2.565 | 4.434E-04 |
| TCONS_00246561 | LINC871 | lncRNA | -3.195 | 3.968E-06 | -3.108 | 4.673E-05 |
| TCONS_00259458 | TBXAS1-OT1 | lncRNA | 1.660 | 2.179E-03 | 2.320 | 4.815E-06 |
| TCONS_00274958 | NKX3-1-OT1 | lncRNA | -5.082 | 1.664E-16 | -4.383 | 2.232E-12 |
| TCONS_00278876 | FAM49B-OT2 | lncRNA | 2.366 | 7.429E-07 | 1.934 | 4.405E-04 |
| TCONS_00282965 | RASEF-AS1 | lncRNA | -1.243 | 6.109E-04 | -1.675 | 4.609E-06 |
| TCONS_00297630 | CDR1-AS1 | lncRNA | -3.419 | 7.544E-09 | -3.213 | 1.466E-06 |

**Table S3.** Top 50 DE-mRNAs of CRSwNP+AS versus CRSwNP-alone

| **Gene identifier** | **Gene name** | **Gene locus** | **CRSwNP+AS (FPKM)** | **CRSwNP-alone (FPKM)** | **Log2(Fold Change)** | ***P* value** | **Adjust *P* value** |
| --- | --- | --- | --- | --- | --- | --- | --- |
| ENSG00000177984 | LCN15 | 9:139654086-139660707 | 44.985 | 0.408 | 6.906 | 2.393E-04 | 9.589E-03 |
| ENSG00000188076 | SCGB1C1 | 11:193080-195940 | 43.621 | 0.244 | 5.134 | 3.332E-08 | 5.440E-05 |
| ENSG00000211891 | IGHE | 14:106064028-106071694 | 60.747 | 1.859 | 5.127 | 4.482E-10 | 2.195E-06 |
| ENSG00000177272 | KCNA3 | 1:111214310-111217655 | 3.156 | 0.131 | 4.814 | 2.246E-04 | 9.382E-03 |
| ENSG00000019169 | MARCO | 2:119699742-119752268 | 29.627 | 1.492 | 4.451 | 2.656E-09 | 8.675E-06 |
| ENSG00000186191 | BPIFB4 | 20:31641464-31699557 | 111.619 | 7.64 | 3.877 | 1.042E-04 | 6.359E-03 |
| ENSG00000185897 | FFAR3 | 19:35849036-35864275 | 5.673 | 0.538 | 3.465 | 1.004E-08 | 2.186E-05 |
| ENSG00000179914 | ITLN1 | 1:160846329-160854960 | 26.375 | 2.499 | 3.462 | 4.183E-05 | 3.692E-03 |
| ENSG00000230657 | PRB4 | 12:11460017-11463369 | 1.649 | 0.17 | 3.408 | 3.239E-06 | 8.033E-04 |
| ENSG00000223609 | HBD | 11:5246694-5256600 | 1.318 | 0.199 | 2.947 | 1.271E-05 | 1.872E-03 |
| ENSG00000015413 | DPEP1 | 16:89679716-89705204 | 4.246 | 0.659 | 2.766 | 2.242E-06 | 6.447E-04 |
| ENSG00000182566 | CLEC4G | 19:7793843-7799013 | 11.485 | 1.874 | 2.750 | 3.092E-05 | 3.217E-03 |
| ENSG00000167419 | LPO | 17:56295909-56360231 | 1.771 | 0.263 | 2.644 | 9.399E-07 | 4.294E-04 |
| ENSG00000170373 | CST1 | 20:23666277-23807368 | 1290.06 | 221.309 | 2.644 | 4.669E-03 | 4.577E-02 |
| ENSG00000145850 | TIMD4 | 5:156342159-156390266 | 2.278 | 0.351 | 2.615 | 9.645E-05 | 6.057E-03 |
| ENSG00000147206 | NXF3 | X:102330528-102348157 | 1.428 | 0.309 | 2.592 | 6.251E-06 | 1.331E-03 |
| ENSG00000179593 | ALOX15B | 17:7942335-7952585 | 6.339 | 0.988 | 2.583 | 6.831E-05 | 4.903E-03 |
| ENSG00000078795 | PKD2L2 | 5:137223657-137278436 | 1.941 | 0.351 | 2.535 | 1.165E-05 | 1.812E-03 |
| ENSG00000124143 | ARHGAP40 | 20:37230433-37280133 | 1.369 | 0.315 | 2.524 | 1.760E-05 | 2.268E-03 |
| ENSG00000109906 | ZBTB16 | 11:113930288-114230324 | 13.392 | 1.828 | 2.524 | 3.721E-07 | 2.917E-04 |
| ENSG00000176387 | HSD11B2 | 16:67464384-67471456 | 12.626 | 2.734 | 2.487 | 5.120E-05 | 4.198E-03 |
| ENSG00000167614 | TTYH1 | 19:54926393-54948080 | 4.912 | 1.085 | 2.466 | 3.571E-05 | 3.436E-03 |
| ENSG00000211898 | IGHD | 14:106303099-106347208 | 66.324 | 12.731 | 2.411 | 2.142E-03 | 3.002E-02 |
| ENSG00000130037 | KCNA5 | 12:5153085-5155949 | 1.363 | 0.282 | 2.399 | 4.928E-08 | 6.897E-05 |
| ENSG00000132915 | PDE6A | 5:149237510-149324356 | 4.039 | 0.907 | 2.373 | 1.702E-06 | 5.874E-04 |
| ENSG00000187922 | LCN10 | 9:139632619-139637808 | 1.895 | 0.436 | 2.358 | 1.034E-05 | 1.688E-03 |
| ENSG00000169194 | IL13 | 5:131991955-131996802 | 3.125 | 0.662 | 2.319 | 3.341E-03 | 3.816E-02 |
| ENSG00000184709 | LRRC26 | 9:140063210-140064503 | 28.318 | 6.045 | 2.312 | 1.259E-07 | 1.298E-04 |
| ENSG00000160678 | S100A1 | 1:153600402-153604513 | 5.478 | 1.137 | 2.265 | 2.716E-05 | 2.990E-03 |
| ENSG00000184451 | CCR10 | 17:40830907-40835935 | 3.211 | 0.746 | 2.251 | 1.130E-05 | 1.771E-03 |
| ENSG00000090920 | FCGBP | 19:40353867-40562128 | 114.312 | 24.098 | 2.249 | 2.738E-05 | 2.997E-03 |
| ENSG00000260001 | TGFBR3L | 19:7981030-7983982 | 0.788 | 0.189 | 2.186 | 8.060E-04 | 1.822E-02 |
| ENSG00000160883 | HK3 | 5:176299475-176326713 | 22.09 | 4.776 | 2.074 | 2.270E-06 | 6.447E-04 |
| ENSG00000110347 | MMP12 | 11:102733467-102745764 | 1.202 | 5.293 | -2.108 | 3.111E-03 | 3.677E-02 |
| ENSG00000151790 | TDO2 | 4:156775890-156841558 | 1.101 | 4.875 | -2.133 | 1.507E-04 | 7.591E-03 |
| ENSG00000204616 | TRIM31 | 6:30069091-30080883 | 0.237 | 1.444 | -2.175 | 2.298E-03 | 3.116E-02 |
| ENSG00000111700 | SLCO1B3 | 12:20960775-21392180 | 0.73 | 3.009 | -2.268 | 1.708E-06 | 5.874E-04 |
| ENSG00000171711 | DEFB4A | 8:7752151-7754233 | 0.376 | 2.165 | -2.290 | 1.996E-03 | 2.908E-02 |
| ENSG00000198758 | EPS8L3 | 1:110292402-110306854 | 0.24 | 1.119 | -2.365 | 1.482E-04 | 7.568E-03 |
| ENSG00000154165 | GPR15 | 3:98250743-98251960 | 0.374 | 1.889 | -2.440 | 1.632E-03 | 2.639E-02 |
| ENSG00000124875 | CXCL6 | 4:74702214-74714781 | 2.622 | 12.327 | -2.561 | 3.346E-07 | 2.851E-04 |
| ENSG00000156234 | CXCL13 | 4:78432907-78532988 | 0.588 | 3.121 | -2.593 | 1.527E-03 | 2.538E-02 |
| ENSG00000173702 | MUC13 | 3:124624275-124672663 | 1.245 | 8.559 | -2.680 | 7.040E-06 | 1.363E-03 |
| ENSG00000267596 | CCL15 | 17:34323476-34329084 | 0.198 | 1.24 | -2.703 | 4.973E-04 | 1.431E-02 |
| ENSG00000177519 | RPRM | 2:154333852-154335322 | 0.156 | 1.084 | -2.711 | 1.450E-04 | 7.517E-03 |
| ENSG00000007306 | CEACAM7 | 19:42177235-42210895 | 0.218 | 1.457 | -2.733 | 4.105E-05 | 3.690E-03 |
| ENSG00000259120 | SMIM6 | 17:73642323-73644057 | 0.253 | 2.308 | -3.026 | 2.627E-04 | 1.000E-02 |
| ENSG00000163735 | CXCL5 | 4:74861205-74864632 | 0.245 | 2.106 | -3.091 | 2.230E-07 | 2.081E-04 |
| ENSG00000168412 | MTNR1A | 4:187448106-187476721 | 0.202 | 1.596 | -3.177 | 9.333E-07 | 4.294E-04 |
| ENSG00000205362 | MT1A | 16:56672578-56673999 | 0.707 | 11.336 | -3.743 | 2.607E-04 | 9.998E-03 |

**Table S4.** Expression of key cytokines and their receptors in nasal tissues from control subjects and CRSwNP-alone and CRSwNP+AS patients

| **Gene name** | **Control** | **CRSwNP-alone** | **CRSwNP+AS** | ***P* value CRSwNP-alone vs. Control** | ***P* value CRSwNP+AS vs. Control** | ***P* value CRSwNP+AS vs. CRSwNP-alone** |
| --- | --- | --- | --- | --- | --- | --- |
| **IFNG** | 0.15±0.20 | 1.59±1.42 | 0.81±1.75 | 0.000 | 0.028 | n.s. |
| **IFNGR1** | 12.20±8.30 | 41.77±6.50 | 27.22±14.36 | 0.000 | 0.008 | 0.050 |
| **IFNGR2** | 22.96±4.79 | 32.23±3.53 | 32.35±5.93 | n.s. | n.s. | n.s. |
| **IL5** | 0.00±0.00 | 0.13±0.20 | 0.58±0.54 | 0.003 | 0.000 | 0.008 |
| **IL5RA** | 1.66±1.18 | 5.13±2.74 | 6.56±4.19 | 0.012 | 0.004 | n.s. |
| **IL5RB** | 4.24±1.26 | 15.75±4.72 | 20.09±8.8 | 0.000 | 0.000 | n.s. |
| **IL4** | 0.02±0.06 | 0.04±0.08 | 0.41±0.48 | n.s. | 0.001 | 0.001 |
| **IL4R** | 19.79±10.88 | 30.58±8.39 | 40.49±11.52 | n.s. | 0.002 | n.s. |
| **IL13** | 0.05±0.06 | 0.66±0.84 | 3.13±2.55 | 0.001 | 0.000 | 0.003 |
| **IL13RA1** | 9.41±4.48 | 23.98±3.98 | 17.31±6.54 | 0.000 | 0.015 | n.s. |
| **IL13RA2** | 0.31±0.31 | 0.38±0.43 | 0.74±0.70 | n.s. | n.s. | n.s. |
| **IL17A** | 0.01±0.01 | 0.16±0.18 | 0.01±0.03 | 0.000 | n.s. | 0.001 |
| **IL17RA** | 7.23±1.85 | 8.93±2.18 | 12.18±4.00 | n.s. | 0.035 | 0.005 |
| **IL17RC** | 31.21±7.36 | 11.27±1.57 | 17.44±8.02 | 0.000 | 0.000 | 0.005 |
| **TNF** | 0.47±0.24 | 2.51±1.35 | 1.47±0.69 | 0.000 | 0.001 | n.s. |
| **TNFRSF1A** | 37.10±9.3.00 | 46.17±9.89 | 55.63±12.65 | n.s. | n.s. | 0.026 |
| **TNFRSF1B** | 10.60±3.61 | 17.73±7.69 | 18.97±7.06 | n.s. | 0.017 | n.s. |
| **IL1B** | 0.98±0.76 | 10.54±10.69 | 5.44±5.41 | 0.000 | 0.000 | n.s. |
| **IL1R1** | 18.89±7.82 | 36.35±13.01 | 30.43±8.51 | 0.029 | n.s. | n.s. |
| **IL1R2** | 1.39±0.76 | 4.80±6.45 | 7.17±6.84 | n.s. | 0.000 | 0.029 |
| **IL6** | 9.43±11.47 | 7.00±5.54 | 14.55±12.03 | n.s. | n.s. | n.s. |
| **IL6R** | 19.03±15.11 | 20.40±4.96 | 32.23±13.84 | n.s. | 0.003 | 0.023 |

**Table S5.** Top 50 DE-lncRNAs of CRSwNP+AS versus CRSwNP-alone

| **Transcript identifier** | **Transcript name** | **Transcript locus** | **CRSwNP+AS (FPKM)** | **CRSwNP-alone (FPKM)** | **Log2(Fold Change)** | ***P* value** | **Adjust *P* value** |
| --- | --- | --- | --- | --- | --- | --- | --- |
| TCONS_00196862 | ALCAM-OT1 | 3:105073425-105146755 | 77.069 | 0.313 | 7.707 | 1.003E-05 | 6.629E-03 |
| TCONS_00012537 | PPP2R5A-OT1 | 1:212458673-212499402 | 17.031 | 0.229 | 6.144 | 7.410E-05 | 1.931E-02 |
| ENST00000563285 | AMFR-012 | 16:56396828-56401515 | 15.732 | 0.215 | 6.075 | 3.540E-05 | 1.285E-02 |
| TCONS_00081540 | PPM1A-OT3 | 14:60715946-60725700 | 13.007 | 0.291 | 5.220 | 3.182E-05 | 1.177E-02 |
| ENST00000503833 | CTSZ-005 | 20:57571642-57582285 | 10.552 | 0.225 | 5.128 | 2.491E-05 | 1.030E-02 |
| ENST00000473573 | COL1A2-007 | 7:94043532-94051352 | 53.705 | 1.279 | 5.062 | 6.097E-07 | 1.246E-03 |
| ENST00000534246 | EIF4G2-016 | 11:10828621-10830466 | 11.511 | 0.466 | 4.480 | 8.378E-05 | 2.085E-02 |
| ENST00000555171 | DHRS7-007 | 14:60622550-60632136 | 11.137 | 0.424 | 4.446 | 4.679E-05 | 1.479E-02 |
| ENST00000592662 | FARSA-008 | 19:13039212-13044512 | 4.569 | 0.246 | 3.995 | 3.993E-05 | 1.410E-02 |
| ENST00000549587 | LMBR1L-017 | 12:49498277-49499015 | 3.572 | 0.18 | 3.947 | 5.039E-06 | 4.701E-03 |
| TCONS_00124905 | MYO1D-OT1 | 17:31113816-31204317 | 7.153 | 0.406 | 3.856 | 1.125E-04 | 2.436E-02 |
| ENST00000566317 | ZC3H18-011 | 16:88695056-88697832 | 3.429 | 0.264 | 3.655 | 2.140E-06 | 3.077E-03 |
| ENST00000494979 | MARCO-004 | 2:119750593-119752234 | 6.205 | 0.404 | 3.643 | 1.831E-06 | 2.734E-03 |
| ENST00000464864 | C7-004 | 5:40976765-40981868 | 7.057 | 0.474 | 3.599 | 8.086E-05 | 2.042E-02 |
| TCONS_00285089 | SET-OT1 | 9:131451302-131458679 | 8.004 | 0.629 | 3.317 | 1.135E-04 | 2.436E-02 |
| ENST00000591302 | MIDN-003 | 19:1251260-1254236 | 2.331 | 0.216 | 3.272 | 1.043E-04 | 2.376E-02 |
| ENST00000482303 | EIF4G1-033 | 3:184045074-184045703 | 5.776 | 0.586 | 3.192 | 1.041E-05 | 6.629E-03 |
| ENST00000512532 | CCNG1-011 | 5:162866197-162868293 | 6.314 | 0.621 | 3.175 | 1.018E-04 | 2.376E-02 |
| ENST00000515528 | HEXB-005 | 5:73981031-73985674 | 8.834 | 0.888 | 3.170 | 2.248E-05 | 9.920E-03 |
| ENST00000585284 | PER1-019 | 17:8044551-8045788 | 12.833 | 1.084 | 3.141 | 1.306E-04 | 2.653E-02 |
| ENST00000501038 | PLXND1-003 | 3:129274057-129275877 | 4.559 | 0.461 | 3.076 | 6.954E-05 | 1.875E-02 |
| ENST00000514666 | HK3-006 | 5:176308303-176308873 | 6.129 | 0.682 | 3.050 | 6.298E-05 | 1.735E-02 |
| ENST00000579052 | MYO15B-022 | 17:73616288-73616952 | 7.619 | 0.789 | 3.021 | 6.283E-06 | 5.422E-03 |
| ENST00000530934 | RAD9A-002 | 11:67160353-67163786 | 5.747 | 0.631 | 3.016 | 5.316E-06 | 4.801E-03 |
| ENST00000469079 | EVA1C-004 | 21:33784314-33840363 | 2.218 | 0.26 | 2.834 | 1.072E-04 | 2.398E-02 |
| ENST00000541602 | ZBTB16-002 | 11:113930447-114060486 | 1.759 | 0.238 | 2.814 | 5.517E-07 | 1.220E-03 |
| ENST00000379050 | TPT1-AS1-020 | 13:45964880-45965683 | 3.965 | 0.453 | 2.803 | 7.720E-05 | 1.999E-02 |
| TCONS_00165313 | ROCK2-OT1 | 2:11389938-11485534 | 1.559 | 0.191 | 2.786 | 4.436E-05 | 1.445E-02 |
| TCONS_00031455 | PTEN-OT1 | 10:89622353-89626806 | 3.529 | 0.451 | 2.731 | 3.002E-05 | 1.143E-02 |
| TCONS_00190781 | LARGE-OT1 | 22:34157547-34316983 | 4.602 | 0.583 | 2.685 | 1.475E-05 | 7.889E-03 |
| ENST00000573311 | SRRM2-023 | 16:2818082-2819548 | 19.079 | 2.668 | 2.674 | 6.219E-05 | 1.725E-02 |
| ENST00000537488 | ATN1-005 | 12:7048270-7051220 | 5.698 | 0.842 | 2.632 | 7.242E-05 | 1.900E-02 |
| ENST00000491311 | CDK11A-019 | 1:1635043-1636041 | 2.362 | 0.31 | 2.620 | 1.305E-04 | 2.653E-02 |
| ENST00000573740 | ALOX15-004 | 17:4542222-4544973 | 83.288 | 11.9 | 2.600 | 7.207E-05 | 1.900E-02 |
| ENST00000569183 | WDR59-021 | 16:74907977-74920133 | 1.072 | 0.173 | 2.499 | 2.145E-05 | 9.576E-03 |
| ENST00000541029 | ATN1-004 | 12:7046861-7048128 | 24.23 | 3.884 | 2.402 | 5.298E-05 | 1.597E-02 |
| ENST00000492479 | PTPRE-011 | 10:129861183-129869105 | 1.453 | 0.232 | 2.387 | 1.094E-04 | 2.398E-02 |
| ENST00000590020 | NEDD4L-026 | 18:55990537-56001252 | 2.424 | 0.402 | 2.383 | 9.493E-05 | 2.262E-02 |
| ENST00000557307 | SYNE2-023 | 14:64679529-64681242 | 8.352 | 1.548 | 2.348 | 1.495E-04 | 2.831E-02 |
| TCONS_00078644 | STK24-OT1 | 13:99171743-99229884 | 11.952 | 2.058 | 2.332 | 1.459E-05 | 7.889E-03 |
| TCONS_00090857 | SPRED1-OT1 | 15:38544266-38575748 | 12.545 | 2.183 | 2.305 | 7.344E-06 | 6.071E-03 |
| ENST00000561949 | MAPK8IP3-014 | 16:1813923-1815054 | 10.45 | 1.953 | 2.292 | 1.912E-05 | 9.314E-03 |
| ENST00000488701 | C1S-009 | 12:7168048-7170112 | 17.463 | 3.289 | 2.216 | 6.763E-05 | 1.849E-02 |
| TCONS_00231342 | ZDHHC11-OT3 | 5:682503-776069 | 1.721 | 0.323 | 2.197 | 1.181E-05 | 7.281E-03 |
| TCONS_00261177 | ZNF12-OT1 | 7:6744863-6746592 | 15.627 | 2.952 | 2.193 | 1.096E-05 | 6.868E-03 |
| ENST00000489523 | AGR2-004 | 7:16839198-16844623 | 10.858 | 49.414 | -2.169 | 5.387E-05 | 1.597E-02 |
| ENST00000488721 | ANKRD36C-002 | 2:96518038-96525677 | 3.481 | 14.628 | -2.379 | 8.908E-06 | 6.622E-03 |
| ENST00000574653 | TOM1L1-019 | 17:53016142-53038774 | 0.156 | 1.01 | -2.711 | 1.312E-04 | 2.653E-02 |
| ENST00000531153 | ANKRD36C-006 | 2:96521756-96573247 | 0.605 | 3.588 | -2.741 | 3.442E-06 | 4.050E-03 |
| ENST00000497797 | CP-007 | 3:148905706-148917608 | 0.415 | 4.26 | -3.322 | 1.946E-05 | 9.314E-03 |
